# Identification of nucleotide patterns enriched in secreted RNAs as putative *cis*-acting elements targeting them to exosome nano-vesicles

**DOI:** 10.1186/1471-2164-12-S3-S18

**Published:** 2011-11-30

**Authors:** Arsen O Batagov, Vladimir A Kuznetsov, Igor V Kurochkin

**Affiliations:** 1 Department of Genome and Gene Expression Data Analysis, Bioinformatics Institute, 30 Biopolis str #07-01, Singapore, 138671

## Abstract

**Background:**

Exosomes are nanoscale membrane vesicles released by most cells. They are postulated to be involved in cell–cell communication and genetic reprogramming of their target cells. In addition to proteins and lipids, they release RNA molecules many of which are not present in the donor cells implying a highly selective mode of their packaging into these vesicles. Sequence motifs targeting RNA to the vesicles are currently unknown.

**Results:**

*Ab initio* approach was applied for computational identification of potential RNA secretory motifs in the primary sequences of exosome-enriched RNAs (eRNAs). Exhaustive motif analysis for the first time revealed unique sequence features of eRNAs. We discovered multiple linear motifs specifically enriched in secreted RNAs. Their potential function as *cis*-acting elements targeting RNAs to exosomes is proposed. The motifs co-localized in the same transcripts suggesting combinatorial organization of these secretory signals. We investigated associations of the discovered motifs with other RNA parameters. Secreted RNAs were found to have almost twice shorter half-life times on average, in comparison with cytoplasmic RNAs, and the occurrence of some eRNA-specific motifs significantly correlated with this eRNA feature. Also, we found that eRNAs are highly enriched in long noncoding RNAs.

**Conclusions:**

Secreted RNAs share specific sequence motifs that may potentially function as *cis*-acting elements targeting RNAs to exosomes. Discovery of these motifs will be useful for our understanding the roles of eRNAs in cell-cell communication and genetic reprogramming of the target cells. It will also facilitate nano-scale vesicle engineering and selective targeting of RNAs of interest to these vesicles for gene therapy purposes.

## Background

Exosomes are small (50-150 nm) membrane vesicles released from various cell types, e.g. from hematopoietic cells (B-cells, T-cells, dendritic cells, mast cells), endothelial, fibroblastic, neuronal and tumor cells [1]. The secretion of exosomes is a conserved process in animal cells that plays an important role in a number of physiological processes including immune surveillance [2], inflammatory response [3] and development [4]. Exosome function depends on the cell type from which they are derived. Besides a constitutive release of exosomes by the cells, their secretion is enhanced upon activation by various stimuli, e.g by changes in intracellular calcium in platelets and mast cells [5] or cell depolarization in neurons [6]. Exosomes contain a spectrum of specific suRNAce molecules that allows their interaction with particular cells in the body. For example, the vesicles shed from neutrophils interact with platelets [7], but those shed from platelets interact with monocytes, but not with neutrophils [8].

While the existence of exosomes has been known for over three decades [9], they have recently attracted a great interest because of their increasingly recognized role in intercellular communication [10]. In addition to proteins, lipids and their bound carbohydrates, exosomes were found to contain mRNA and miRNA [11]. Moreover, in the recipient cells RNA can be translated into protein in the case of mRNAs [11], or repress the expression of other genes in the case of miRNAs [12]. The fact that exosomes contain RNA suggests their important role in the horizontal transfer of genetic information between cells in the body. This has important implications for the processes of development and disease. For example, exosomes released from murine embryonic stem cells induce an epigenetic reprogramming of target cells [13]. Tumor-derived exosomes have been found to contain a subset of mRNAs associated with signaling pathways relevant for tumor cell survival, growth, host tissue invasion, and metastasis [14]. Exosomal miRNA expression profiles have been shown to have signatures related to tumor classification, diagnosis, and disease progression [15]. Thus exosomal RNAs (eRNAs) provide potential new targets for diagnostic and therapeutic applications [16]. Exosomes are also being considered as promising nanoscale machines for the delivery of therapeutic RNAs for the treatment of various conditions ranging from cancer to diabetes [17].

Intriguingly, several studies detected many of the mRNAs and miRNAs exclusively in exosomes suggesting a nonrandom fashion of packaging of the RNAs into these microvesicles. These observations raise a number of mechanistic questions regarding the pathways for targeting of RNA into exosomes. In the case of cell-bound mRNAs, their targeting is a highly selective process contributing to the formation of sub-cellular domains and cell asymmetry [18]. A high-throughput *in situ* hybridization screen in a model organism *Drosophila* revealed that 71% of the transcripts are localized in a large number of different patterns, suggesting that specific mRNA localization is a widespread phenomenon [19].

mRNA localization depends on interactions between *cis*-acting elements in the mRNA sequence referred to as “zipcodes” and trans-acting factors, the RNA-binding proteins. A number of *cis-*acting elements have been identified in localized RNAs [18]. These elements are recognized by the transporting machinery based on sequence, structure, or both, although it is often difficult to probe sequence and structural requirements independently. Perhaps the best studied example of structural localization element in mRNA is that of the *Drosophila bicoid* (*bcd*) mRNA [20]. This element is represented by a helix in which nucleotide identities are not important [20]. In the case of the TLS, an RNA sequence element that mediates the subcellular localization of *K10* and *Orb* transcripts in *Drosophila* oocytes, both a stem–loop secondary structure and specific nucleotide sequences are required for the recognition by trans-acting cellular factors [21]. Each localized mRNA contains one [22] or more [23]* cis*-acting sequence elements and most known localization signals so far found are present in the untranslated regions [18].

Several experimental approaches have been successfully utilized to identify zipcodes - observation of localization of the mRNA molecules following fragment deletions or point mutations in their sequence. Computational prediction of zipcodes proved to be very difficult for several reasons. First, the same RNA may interact with a large number of trans-acting factors, each utilizing particular mode of target recognition [18]. Second, RNA localization signals operate at the level of both primary and secondary structure. In addition to the fact that RNA secondary structure depends on the context, many prediction methods ignore non-canonical base pairings and pseudoknots [24]. Even prediction methods for the sequence-based motifs had very limited success because of the short length of these motifs and their combinatorial organization [18]. It should be noted that all previous attempts to computationally predict mRNA localization signals were based on a priori knowledge of experimentally verified motifs, which they mapped on to novel RNA sequences. However, despite increasing interest in exosome biology in the past years, no experimental studies on elements targeting RNA for secretion have been performed and thus RNA secretory zipcodes remain unknown. Therefore computational prediction of sequence motifs responsible for RNA secretion is a very challenging task. Apart from its academic interest, discovery of these motifs will be useful for engineering and selective targeting of RNAs of interest to exosomes for the gene therapy purposes.

In this work, we have applied *ab initio* approach for computational identification of potential RNA secretory motifs that does not require any prior knowledge of motif structure and is based on the comparison of primary sequence of eRNAs with cell-bound RNAs. We describe for the first time short linear motifs specifically enriched in secreted RNAs and discuss their potential function as *cis*-acting elements targeting RNAs to exosomes.

## Results

At present, only few studies reporting quantitative measurements of RNA secretion exist. The most detailed set, obtained by Skog with colleagues, was used in the present study [25]. In this report, only the fraction of transcripts present exclusively in secreted vesicles was analyzed. We aimed at a more informative analysis whereby transcripts could be classified according to their level of enrichment and other quantitative parameters of eRNAs in comparison with intracellular RNAs. The following parameters were considered: i) transcript length, ii) half-life in host cells, iii) base composition, iv) gene ontology (GO), v) RNA class (messenger or non-protein coding). No significant difference in base composition and GO were found for eRNAs (data not shown). We detected a statistically significant, but small difference for RNA length (Additional File 1).

The eRNAs expression was compared with the expression of cell-bound RNAs and exosomic to cell-bound expression ratio (ECER) was calculated. Exosome-enriched RNAs with expression values 100 and higher were stratified into i) exosome-specific (present in exosome, but not in cell), ii) strongly enriched, with ECER>33, iii) moderately enriched, with 2<ECER<33, iv) weakly enriched, with 1.5<ECER<2.

### The proportion of long non-protein coding RNAs is increased in eRNA fraction

**Figure 1:**
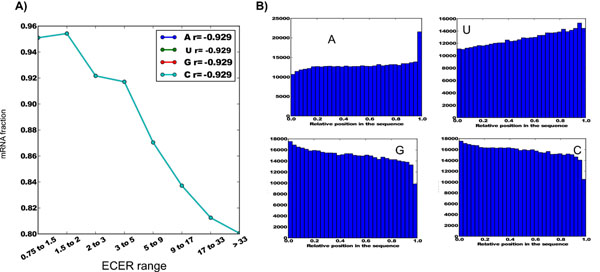
**General characteristics of base composition in eRNAs**. A) Fraction of mRNAs in eRNAs negatively correlates with their secretion without a significant bias in base composition. B) Base distribution along the length of the studied RNAs.

Out of 757 cell-bound RNAs (ECER from 0.75 to 1.5), 720 (95%) were mRNAs and 37 (5%) were non-protein coding transcripts. As the ECER number increased, the fraction of mRNAs decreased from 95 to 80% (Figure 1A), while the complementing fraction of long non-protein coding RNAs increased from 5 to 20% (75/376). Thus, the fraction of non-protein coding RNAs increased 5 times in secreted RNA fraction, in comparison with intracellular. A strong negative correlation (-0.9) was observed between the mRNA content and ECER, while for non-protein coding RNAs this correlation was strongly positive (0.9).

### eRNAs have shorter half-life time than intracellular RNAs

**Figure 2:**
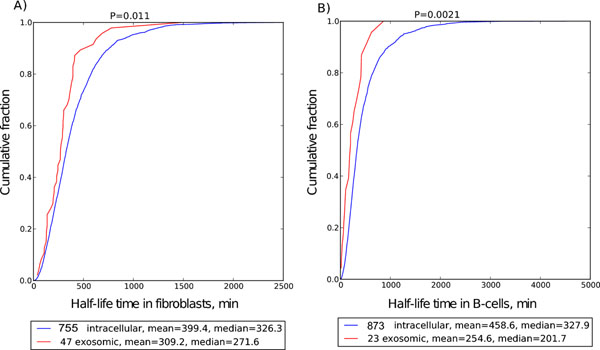
Half-life time distributions of intracellular (ECER from 0.75 to 1.5) and secreted (ECER > 33) RNAs in A) fibroblasts and B) B-cells.

We analyzed previously published transcriptome-scale experimental data on RNA half-lives in fibroblasts and B-cells [26] and found a significant difference in half-life time distribution between eRNAs and intracellular RNAs (Figure 2). The largest difference was observed in B-cells (P=0.0021) with the mean half-life time 1.8-times longer for intracellular RNAs. In fibroblasts, intracellular RNAs half-life was only 1.3-times longer, but still the difference with eRNA fraction was statistically significant (P=0.011).

### Multiple alignments and position-specific scoring do not reveal long or short sequence elements specific to eRNAs

Due to unknown nature of potential *cis*-acting motifs enriched in secreted RNAs, as a starting point of the study, two traditional approaches were considered to identify common *cis*-elements in secreted RNAs - multiple sequence alignment (MSA) and position-specific scoring matrix (PSSM) models. Multiple alignment strategy aims to reveal position-independent regions similar in a large fraction of sequences. The span of such regions can be very long and their expected span can be controlled, as in Clustal [27], or defined by a search heuristic, as in MUSCLE [28]. This approach favoring searches for sparse and/or long regions, is sensitive to over-representation in multiple sequences, but is insensitive to positional context information within the same sequence. This can be compared with PSSM approaches, implemented in tools like BioProspector [29] and MEME [30], which are specified at discriminating over-represented sequence regions based on their positional context. Such tools are used for discovery of short elements in a single or multiple sequence contexts, such as transcription factor binding sites. Thus, both strategies complement each other and could potentially be successful to discover novel signals in eRNAs.

Among the multiple alignment algorithms, Clustal was selected as a method for which the expected scarcity and length of potential eRNA-specific motifs could be controlled by setting score penalties for opening and extending gaps in the alignment. Among PSSM approaches, BioProspector was selected, since, unlike most of PSS algorithms, it features high-order Markov models, which allows one to discover longer and more interspersed sequence motifs [31].

Neither Clustal nor BioProspector could reveal any significant motifs in all ECER ranges, apart from poly(A) sequences (data not shown). Thus the application of both multiple alignment and PSSM strategies was unsuccessful. Therefore, an exhaustive motif search, which is not biased to neither positional, nor multiple sequence context, was applied.

### Exhaustive motif search

Oligonucleotide representation is a specific approach of motif search recently emerging with increasing accessibility of computational power. It is unbiased both in the sense of positional context and multiple sequence sets. For example, it was implemented in RSAT, a popular tool for motif discovery [32]. In the current study, a higher level of flexibility was required in comparison with RSAT, mainly due to the large number of oligonucleotides to be tested as described below and the diversity of statistical parameters to be estimated.

The exhaustive algorithm generates all possible oligonucleotides of a given length and ranks them by their statistical properties in each data set. Varying the oligonucleotide length from 1 to 8 can retrieve a wide range of information about the studied sequences -from the position-dependent biases in base composition to potential motif repeats with hundreds nucleotides repeat period, which can not be captured with other methods. Short element position-specific oligonucleotide biases revealed by this method can potentially be used as starting points for further more detailed study of these features as discriminative characteristics of a sequence set, as it is demonstrated in the present study. This approach, however, has some limitations. For instance, it is not feasible to use for studying degenerate motifs, such as AC[UAC]AA, [UA]AAU, where square brackets surrounding a nucleotide position stand for all possible substitutions at this position with a letter surrounded by the brackets. Evaluation of such cases is possible. However, if we would like to keep our strategy exhaustive, making even a single position degenerate to *m* possible nucleotide types, this approach would result in m^n^ times more combinations to be screened. In addition, this type of motif does not add as much information about sequence specificity as analysis of co-localization of shorter motifs, which are contained within a given long motif. And multiple motif positional co-localization was out of scope of the present study as well.

For each oligonucleotide combination (motif) of length from 1 to 8, we analyzed the frequency of occurrence in a given set of sequences, the fraction of mRNAs (according to Refseq annotation), the skewness of motif location distribution along the length of the motif-containing RNAs and other parameters (see Additional File 2). The motifs were ranked first by skewness and second- by representation in the RNA sequences. Based on cutoff values of these parameters (skewness absolute value greater than 0.6, fraction of RNAs greater than 15%) and enrichment in eRNAs, motif classes were defined as i) associated with eRNAs, ii) associated with intracellular RNAs. An in base pair distribution along the length of the RNAs was observed. Adenine was enriched in the 3’-region representing a significant fraction of polyadenylated RNAs (Figure 1B). G/C-rich sequences were found with higher frequencies in 5’-region. This tendency was observed for both cell-bound and secreted RNAs. No significant enrichment in exosomes and correlation with ECER were observed for any base pair.

### Short eRNA motifs negatively correlate with RNA life time and mRNA fraction

**Figure 3:**
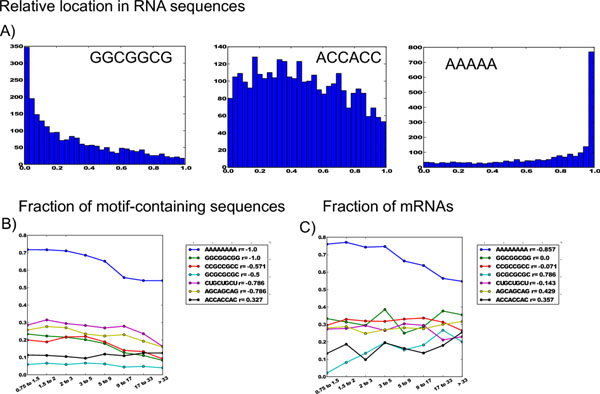
**Statistical properties of short repetitive motifs in secreted RNAs**. A) Examples of unusual distributions of short motifs along the RNA sequence length. B) Short motif presence negatively correlates with RNA secretion. C) Short motifs segregate into positively correlating, negatively correlating and not correlating with mRNA fraction in secreted RNAs.

**Figure 4:**
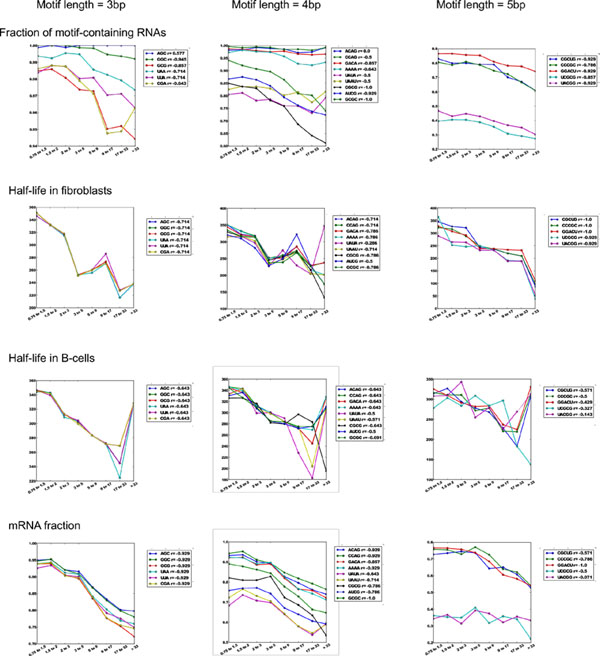
**Correlations between short motif occurrence and RNA parameters**. The separation of the motifs into distinct RNA classes is more recognizible as the length of the motifs is increased. Correlation between secretion of RNAs and their half-lives increases for specific motif combinations starting from 5bp length.

Surprisingly, all 3-5-nt-long motifs correlated negatively with secretion and had a strong negative correlation with RNA half-lives in both fibroblasts and B-cells (Figures 3 and 4). Thus, it can be concluded that the observed short intracellular life time of secreted RNAs does not depend on their base pair composition, but is rather their universal property. Negative correlation of secretion with mRNA fraction was observed in general (see above). Motifs marking RNA polyadenylation correltated negatively as well. The fraction of mRNAs decreased from 95% in non-secreted fraction (ECER from 0.75 to 1.5) to 75% in strongly secreted fraction (ECER > 33) (Figure 3C).

### Repetitive short motifs have a distribution skewed to 5’- and 3’- ends of eRNAs

To assess the impact of short (3-5-nt-long) motif repeats, we also analyzed longer motifs (8-nt-long) containing them as subsequences. Among short motifs (3-5-nt-long), GC-rich repeats (GGC, GCC) were enriched in the 5’-end of eRNAs, while the 3’-end had a large fraction of poly(A) repeats (Figure 3A). At the same time, ACC-containing repeats were enriched in the central part of eRNAs. It is remarkable that 3’-poly(A), along with 5’-GGC repeats, demonstrated a strong negative correlation with secretion. The fraction of poly(A) reduced from 71% in intracellular RNAs (0.75<ECER<1.5) to 56% in eRNAs (ECER>33). Unlike 3’-poly(A), 5’-poly(GGC) content did not correlate with the mRNA fraction.

### Secretion-specific long motifs with high complexity account for 30% of eRNAs

Since a negative correlation between RNA secretion and RNA half-lives time in our data was observed to be a general rule, it could be used as a parameter indicating RNA secretion specificity of the long motifs, rather than their biological function. We used RNA half-life time correlation coefficient, along with the fraction of motif-containing RNAs, as primary parameters for selection of the top secretion-specific motifs. In addition, skewness was used to assess the specificity of the motifs to particular locations within the RNA sequence span, attributing to their relationship to spatial RNA structure.

**Figure 5:**
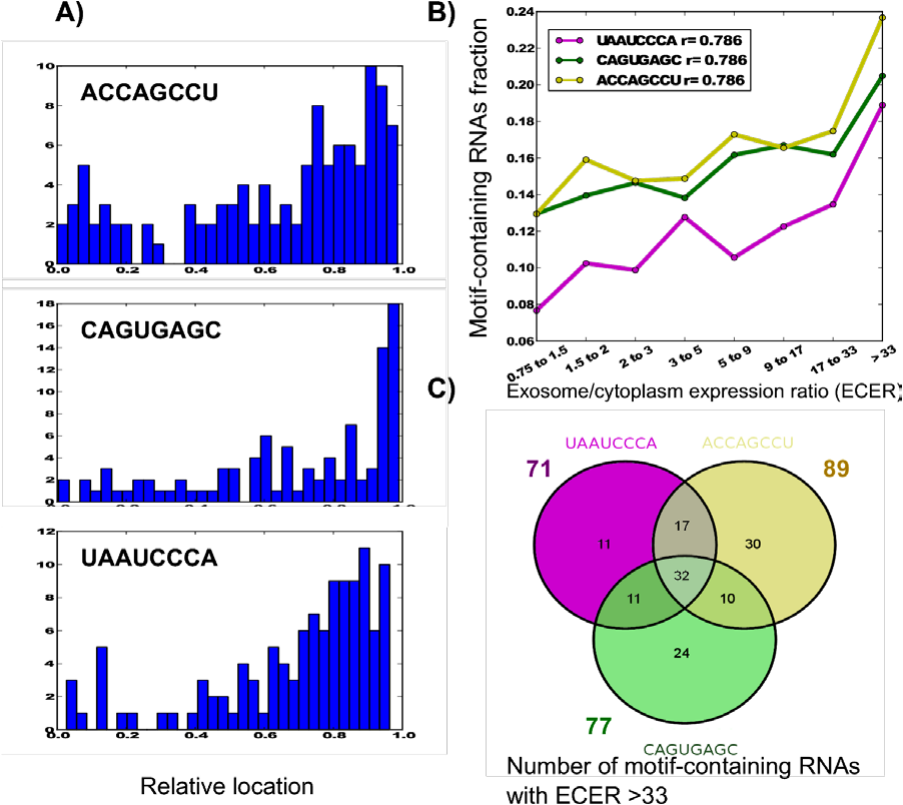
**Motifs in eRNAs selected by a strong positive correlation with exosome secretion in glyoblastoma cells and a significant correlation with RNA half-lives in fibroblasts**. A) Distribution of the 3 motifs along the RNA length. B) Correlation of the occurrence of the 3 motifs in eRNA with ECER. C) The Venn diagram showing the occurrence of the three motifs in the fraction of eRNAs with ECER >33.

**Figure 6:**
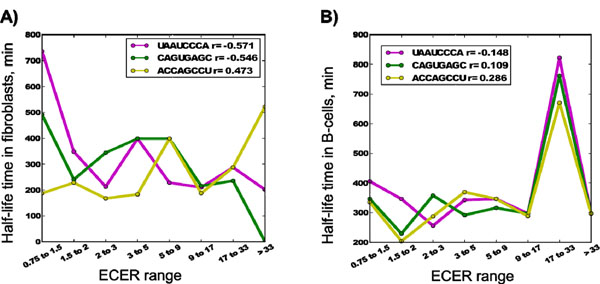
**Independent presence of two of the three secretion-specific motifs (UAAUCCCA and CAGUGACG) in RNAs negatively correlates with their half-life time, while one of them (ACCAGCCU) correlates positively**. Half-lives of RNA for fibroblasts (A) and B-cells (B) were obtained from [26].

We identified 145 8bp-long motifs satisfying the above parameters with both RNA secretion fraction and half life-time correlation coefficient absolute values not less than 0.7 and 0.4, respectively. Among them, only 12 motifs positively correlated with secretion and only 6 positively correlated with the half-life time. The sequence locations of 76 motifs were skewed towards 5’-end of RNAs (skewness > 0.2 at ECER > 33), while only 22 motifs were skewed towards 3’-end. Although the motifs strongly correlated with secretion, the fraction of the RNAs containing them did not exceed 24%, except for AAAAAAAA. This fraction was above 10% for only 62 of them. Thus, most of the motifs could be associated with only a small fraction of eRNAs. Overall, only three motifs (ACCAGCCU, CAGUGAGC and UAAUCCCA) satisfying all four criteria were chosen for further evaluation as potentially specific for RNA secretion (Figure 5). It is remarkable that RNAs containing these motifs show divergent correlation patterns between secretion and half-life time, with two of them (UAAUCCA and CAGUGAGC) correlating negatively and one (ACCAGCCU) positively in fibroblasts (Figure 6A). No significant correlation of these motifs with RNA half-life time was observed in B-cells (Figure 6B).

Although each motif was found only in a small fraction of highly secreted RNAs (ACCAGCCU - 24%, CAGUGAGC - 20%, UAAUCCCA – 19%, ECER > 33), the sets of secreted RNAs containing them together, revealed a remarkably significant overlap (32 RNAs) (Figure 5C and Additional File 3). Thus, the combination of these 3 motifs is preferred in eRNAs over single and double motif co-occurrence. The fraction of eRNAs (ECER > 33) containing, at least, one of these motifs was only 36% (135 RNAs) because of the strong co-occurrence of these motifs within the same transcripts. GO analysis did not reveal any functional group significantly over-represented within this set of eRNAs. Only 67% (91/135) of the RNAs were present in GO databases. These RNAs include both mRNAs and non-protein coding RNAs (Additional File 2). The results suggest that the only observed specificity of this three motif combination is RNA secretion.

**Figure 7:**
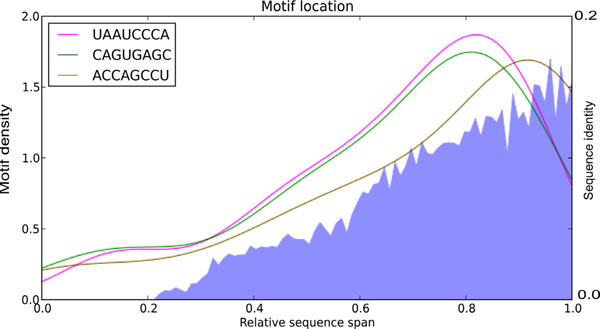
**Mapping of approximated density of eRNA-specific motifs UAAUCCCA, CAGUGAGC, and ACCAGCCU in the selected 32 sequences**. The similarity function of the selected 32 eRNAs (Additional File 6) was calculated as described in Methods. A spatial representation of each motif along the length of sequences containing it was calculated. A relative scale of RNA length was used in this case, with 1 taken as the total RNA length. Mean sequence identity across the eRNAs is shown as a blue filled area.

To test if the presence of discovered motifs in eRNAs was associated with some larger (hence, more general) primary RNA structures, the 32 RNAs containing all 3 motifs were aligned using Clustal. Surprisingly, both mean sequence similarity and the density of motifs localization were increased towards 3'-end of these RNAs (Figure 7). This result demonstrates that the discovered specific sequence motifs mark a larger and sparser structure specific for eRNAs.

### Secondary structure analysis reveals similarities in the folds of secretion-specific motifs within different RNA molecules

**Figure 8:**
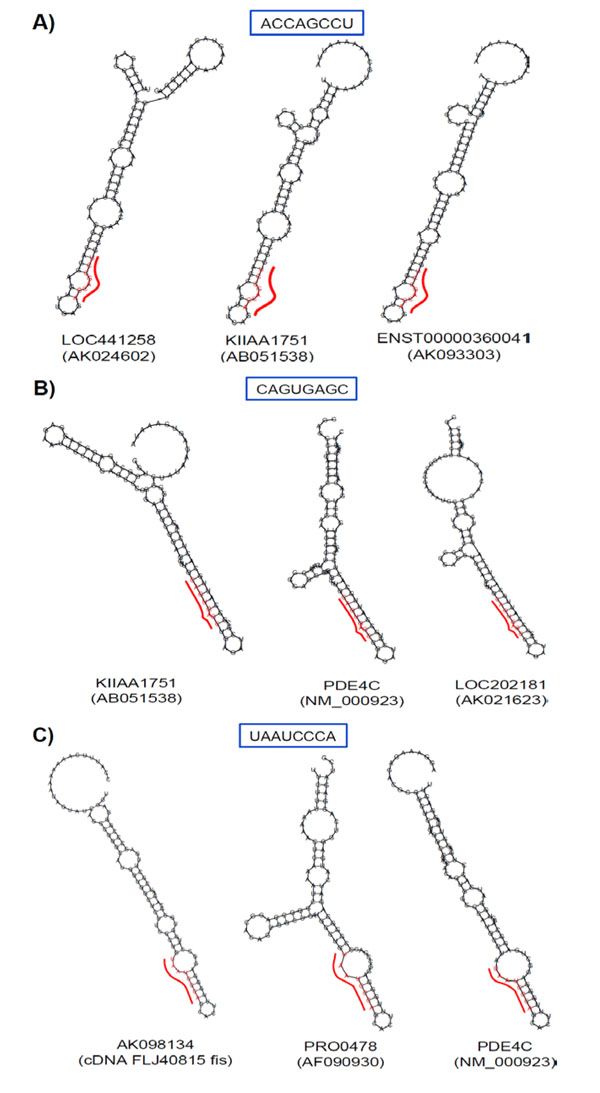
**Predicted RNA secondary structure of the identified motifs**. RNA sequences spanning 100 nucleotides that include 46 nt upstream and 46 nt downstream of the particular 8-nt-long motif were analyzed by RNAfold. The centroid structures predominant for the given motif are shown. The 8-nt-long motif sites are indicated by red bars.

To investigate if the secondary structure of the motifs and their adjacent sequences are conserved in this region as well, a computational analysis of RNA secondary structures was carried out. RNA sequences spanning 100 nucleotides that include a particular 8-nt-long motif were analyzed using the program RNAfold (see Methods for details). Among the RNA folds for all the 8-nt-long motifs found in the 32 eRNAs (see above) (Additional files 3, 4 and 5), we focused on the secondary structures derived from the sequence region 0.7 to 1.0 of full length transcripts (Fig. 7). We ranked all the RNA secondary structures in this region according to their lowest free energy (Additional file 5) and selected for a detailed analysis the most highly ranked and more frequently occurring centroid and minimum free energy (MFE) predictions. Remarkably, each of the 3 identified motifs was predicted to form strikingly similar secondary structure within different RNAs, even when those RNAs had distinct overall structures (Figure 8). The ACCAGCCU motif was found most often as a part of a structure comprising a stacked pair, an internal loop and a helix of three base pairs (Figure 8A). The CAGUGAGC sequence was typically embedded in a highly paired stem interrupted by a bulge loop at position 6 of the motif (Figure 8B). The UAAUCCCA motif was found as a part of an internal loop followed by a 5-base-paired helical region (Figure 8C).

## Discussion

Current study is focused at discovering motifs enriched in eRNAs. To confirm that the described motifs are specific to eRNAs, rather than to their biological functions, general RNA parameters were assessed. Secreted fraction of RNAs is inhomogeneous in all major aspects – amount of RNA, vesicle enrichment ratio, features of primary structure. Since exosome secretion of RNAs diminishes their intracellular concentration, quantitative reverse relationship between intracellular and secreted RNA concentration is naturally expected, however, has never been demonstrated before. It is generally assumed that cellular RNA levels are determined by the interplay of processes for RNA production (transcription) and degradation. Despite the fact that RNA secretion is already accepted phenomenon, the significant implications of this statement are not taken into account by various models analyzing regulation of cellular RNA levels. We report here for the first time that secreted RNAs have almost twice shorter half-life times in average than intracellular RNAs.

We found characteristic sequence features, distinguishing eRNAs from intracellular RNAs: i) mRNA and 3'-polyadenylated RNA fractions are decreased, ii) RNAs with short (3-4-nt-long) repetitive elements in the 5'-end are less frequent, iii) multiple long sequence motifs, specific to eRNAs, are present in up to 25% of eRNAs, iv) the presence of some specific long motifs in eRNAs correlates strongly with their short intracellular life time.

Using exhaustive search, we proved for the first time that there is no single motif specifically associated with the majority of eRNAs. Rather than that, there are combinations of multiple motifs which are specific. We studied in detail one such combination of 3 long motifs specifically located at the 3'-end of eRNAs. We found that this motif combination is a part of a larger region with strong sequence similarity in 32 top secreted eRNAs. Since we observed multiple motif combinations, we hypothesize that this structure may serve as a substrate of several RNA-binding proteins, parts of a large RNA-targeting machinery directing eRNAs to exosomes. Each of the 3 identified in this study motifs was predicted to form very similar secondary structures within different types of RNAs. These secondary structure elements may serve as binding sites for cognate RNA-binding factors.

Interestingly, intracellular mRNAs have been shown to contain more than one linear motif used for subcellular localization. In some cases, these could be clusters of the same motif like the repeated motif UUCAC essential for localization of the Vg1 mRNAs in *Xenopus* oocytes [33] or GCAC motif identified in *Xpat* mRNA, fifteen copies of which are present in a 526-nt window [34]. Single short motifs have typically very weak localization function on their own, while multiple tandem copies confer substantial localization. This organization of RNA localization elements is reminiscent of *cis*-regulatory elements in DNA that are usually composed of clusters of repeated binding sites for transcription factors [35]. Organization of RNA localizing motifs could be highly combinatorial. The presence of multiple motifs may reflect discrete pathways working at sorting RNA to exosomes. Consistent with this notion, motif utilization in localized mRNAs is often separated in time and space. For example, transport of rat protein kinase Mζ mRNA is specified by two *cis*-acting dendritic targeting elements. First element, located at the 5'-UTR, directs somato-dendritic export of the mRNA and second element, in contrast, is located in the 3'-UTR and is required for delivery of the mRNA to distal dendritic segments [36]. Although the intracellular traffic of RNAs prior to entry into exosomes is unknown, it is likely to be a very complex process operating through a consecutive exchange of carrier proteins, each recognizing its cognate RNA motif. Exosomes are generated by multiple different pathways and the picture is complicated by the fact that a single cell may produce a mixed population of exosomes [1].

The motifs identified in this study might be necessary but not sufficient for RNA sorting to exosomes. Recognition of these motifs by various trans-acting factors may depend on the sequence context, spacing, location or tertiary structural features. These can be better understood when data on three-dimensional structure of RNA-transport protein complexes become available. The motifs enriched in secreted RNA appear to be distinct from known RNA elements. This implies that the trans-factors that recognize them may be distinct from known RNA-binding proteins.

Our analysis revealed that long non-protein coding RNAs are enriched in a fraction of eRNAs. Moreover, as the ECER number increased, the proportion of long noncoding RNAs also increased. Long noncoding RNAs are increasingly being recognized as having important role in a number of cellular processes [37]. The emerging evidence indicates that these RNAs may control the epigenetic states of cells by targeting chromatin modification complexes and that their expression is deregulated in cancer and other complex diseases [38]. Thus, similarly to secreted miRNAs, long noncoding RNAs may perform regulatory functions in the target cells.

## Conclusions

Computational discovery of motifs in sequences is a fundamental problem of molecular biology. This study provides the first attempt of bioinformatics analysis of enriched motifs in secreted RNA and discusses their utility as potential *cis*-acting elements targeting them to exosomes. Association of the discovered motifs with other RNA parameters has been revealed. Secreted RNAs were found to have almost twice shorter half-life times on average in comparison with intracellular RNAs. The occurrence of some eRNA-specific motifs significantly correlated with this eRNA feature. Prediction accuracy of RNA exosome-targeting signals will improve as new information is added. The methodology applied in this study will be helpful when new data sets for eRNA from different cell types under various conditions become available. The results of this study facilitate the prioritization of targets for further experimental validation. Finally, understanding mechanisms of RNA targeting to exosomes may give us a way to devise artificial secreted vesicles with the desired set of RNAs that can be transferred to recipient cells to modulate their function.

## Methods

### Data sources

The expression data on intracellular and exosomal RNAs used in the current study was previously published in [25] and is publicly available at Gene Expression Omnibus (GSM339549 and GSM339550). The RNA expression values for 40812 transcripts were measured with Agilent microarrays in duplicates. The data on half-lives of 8342 B-cell RNAs and 8173 fibroblast RNAs were obtained from previously published work [26]. RNA sequences were retrieved from GeneBank and stored by their GI accession IDs.

### Data partitioning by exosomal expression

5723 transcripts with average exosomal expression values above 100 and with concordant expression (exosomal vs. intracelluar fractions) in two technical replicates were chosen for further analysis. The transcripts were separated into 8 categories by the ranges of their ratios of exosomal to intracellular expression values (ECER) averaged across the duplicates. The following ranges of ECER values were chosen: 0.75-1.5, 1.5-2, 2-3, 3-5, 5-9, 9-17, 17-33 and >33. The number of transcripts in each category was from 376 (ECER>33) to 918 (1.5<ECER<2). The sets of the genes for which exosomal expression was different from intracellular expression less than 1.5-times (0.75<ECER<1.5) and larger than 33 times (ECER>33) were considered as reference “cell-enriched”’ and “exosome-enriched” sets, respectively. The partitions above the intracellular reference range were chosen with a simple rule of doubling the ECER range span. This choice of partition boundaries resulted in comparable sample sizes within the partition, while staying scalable in respect to the ECER ranges.

### Motif search

To test the data for a possible presence of long common motifs over-represented in the sequences of eRNAs, Clustal algorithm [27] was used with gap open and gap extend penalty parameters varying from 1 to 10. To find short over-represented motifs which sequences are statistically different (in Markov model) from the surrounding background, Bioprospector software tool [29] was used with the background being calculated from the input sequences and motif length varying from 4 to 10. Exhaustive motif search algorithm included the search of all possible combinations of motifs up to 8-nt-long motif as the first step. To characterize the representation of each motif in a given data set, descriptive statistics, as well as expected enrichment and correlations with ECER were calculated (Additional File 2). In addition, a spatial representation of each motif along the length of sequences containing it was calculated. A relative scale of RNA length was used in this case, with 1 taken as the total RNA length. The above procedure was applied to the exosome-enriched sequences and the sequences from the control set. In each set, the motifs were ranked by their occurrence frequencies. Motifs changing their ranks most significantly were selected as having the biggest enrichment in the eRNA sequences.

### Statistical analysis

The significance of the motifs enrichment in a given set of RNA sequences was assessed relative to the following background distributions: Pm - the observed number of occurrences of the motif in given sequences vs. expected number of occurrences of a random motif with the same base composition in given sequences; Ps - the observed number of given sequences containing, at least, 1 given motif versus the expected number of given sequences containing, at least, 1 random motif with the same base composition. The enrichment P-values were estimated based on Fisher's exact test and were corrected for FDR [39]. Quantitative relationship between the RNA sequences containing a given motif and their ECER values was assessed with Kendall correlation coefficients. Comparisons between cumulative distribution curves (motif-containing RNA sequence fraction, mRNA fraction, and half-life times) were made as follows. For each motif, median values for the mentioned RNA parameters were calculated. Data were generated for all motifs of a given length from the sequences of RNAs belonging to a particular range of ECER values. Comparisons were made between cumulative distributions of motifs with the same length, belonging to different ECER ranges, using Mann-Whitney U- test. The problem of imbalanced statistical design and large data sets was addressed with bootstrap resampling procedure. For each comparison, a sample of either a) 100 values or b) the size of the smallest sample was randomly selected from each of 2 compared distributions. The P-value resulting from this comparison was recorded and the distributions were resampled. Resampling continued until the standard deviation of the resulting P-values did not exceed 10% of the resampled P-value, or until both the resampled standard deviation and the mean P- values decreased to 1.0E-15. Gene ontology analysis was performed with DAVID web tool [40].

### Sequence similarity analysis

To calculate the similarity function of the selected 32 eRNAs, a multiple sequence alignment was performed with Clustal (gap open 1, gap extend 5) as the first step. The resulting alignment was read sequence-wise. At the second step, for each sequence the nucleotide span was separated into 100 bins. For every base pair in every bin (covering a region of several base pairs in a given sequence), the fraction of sequences (out of 32) aligned to it was found. Mean values of the fraction of the aligned sequences were calculated for every bin. At the third step, the bins belonging to different sequences were stacked and for every bin the mean value was calculated again. The resulting function was considered as a sequence identity function defined on a given sequence set.

### RNA secondary structure analysis

RNA sequences spanning 100 nucleotides that include 46 nt upstream and 46 nt downstream of the particular 8-nt-long motif were selected for the secondary structure analysis. The secondary structures were predicted using the program RNAfold from Vienna package v.1.8 [41] with parameters -p and -d2. Among the predicted centroid and MFE predictions, predominant (most frequently occurring) structures were identified by visual inspection.

## Competing interests

The authors declare that they have no competing interests.

## Authors' contributions

AB and IVK conceived and designed the project. AB carried out bioinformatics and computational work. AB and IVK wrote the manuscript. VAK contributed to data analysis and interpretation. All authors read and approved the final manuscript.

## Supplementary Material

Additional file 1 **Figure S1**. Statistical characteristics of eRNA motifs. The number of sequences in the ECER partitions. Click here for file

Additional file 2 **Table S1.** Motifs enriched in eRNAs. Click here for file

Additional file 3 **Figure S2**. Secondary structures for sequence region 0 to 0.3 of full length for the selected 32 eRNAs (see Fig. 7). Click here for file

Additional file 4 **Figure S3.** Secondary structures for sequence region 0.3 to 0.7 of full length for the selected 32 eRNAs (see Fig. 7). Click here for file

Additional file 5 **Figure S4**. Secondary structures for sequence region 0.7 to 1.0 of full length for the selected 32 eRNAs (see Fig. 7). Click here for file

Additional file 6 **Table S2**. 32 eRNAs containing the tripartite motif. Click here for file

Additional file 7 **Table S3**. Full list of eRNAs. Click here for file
