# Supplementary figures and images for: Identification of nucleotide patterns enriched in secreted RNAs as putative cis-acting elements targeting them to exosome nano-vesicles (part 1 of 3)
Source: BMC Genomics. 2011 Nov 30;12(Suppl 3):S18. doi: 10.1186/1471-2164-12-S3-S18 (PMC3333177; doi:10.1186/1471-2164-12-S3-S18)

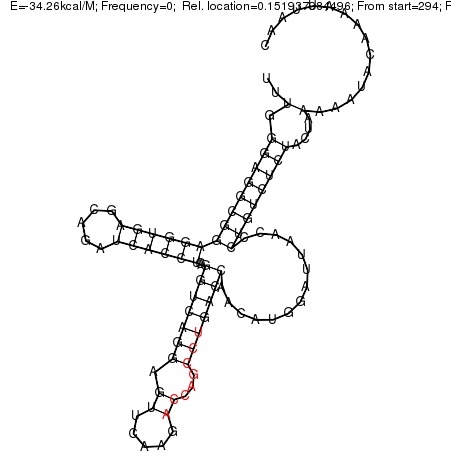

Supplement: Additional file 3 — Figure S2. Secondary structures for sequence region 0 to 0.3 of full length for the selected 32 eRNAs (see Fig. 7). [file 1471-2164-12-S3-S18-S3.zip › Figure S2/Centroid/ACCAGCCU_rank-1_6690226.jpg]

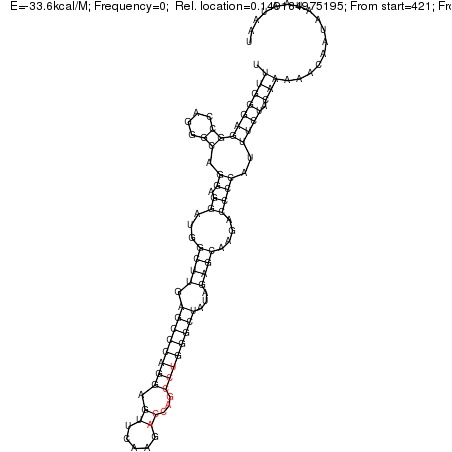

Supplement: Additional file 3 — Figure S2. Secondary structures for sequence region 0 to 0.3 of full length for the selected 32 eRNAs (see Fig. 7). [file 1471-2164-12-S3-S18-S3.zip › Figure S2/Centroid/ACCAGCCU_rank-2_21758081.jpg]

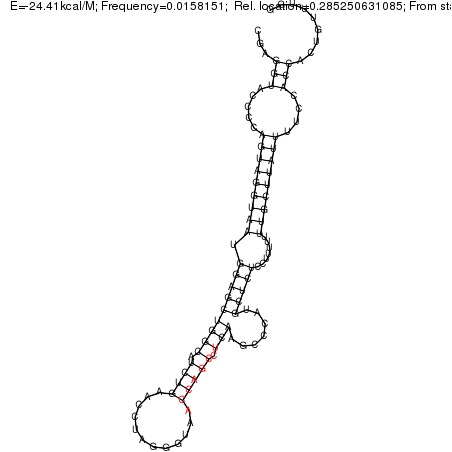

Supplement: Additional file 3 — Figure S2. Secondary structures for sequence region 0 to 0.3 of full length for the selected 32 eRNAs (see Fig. 7). [file 1471-2164-12-S3-S18-S3.zip › Figure S2/Centroid/ACCAGCCU_rank-5_10436764.jpg]

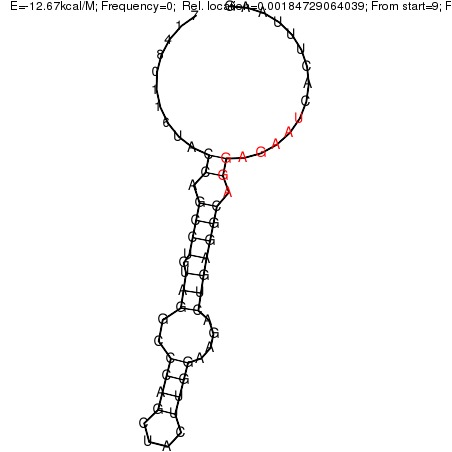

Supplement: Additional file 3 — Figure S2. Secondary structures for sequence region 0 to 0.3 of full length for the selected 32 eRNAs (see Fig. 7). [file 1471-2164-12-S3-S18-S3.zip › Figure S2/Centroid/ACCAGCCU_rank-6_71480116.jpg]

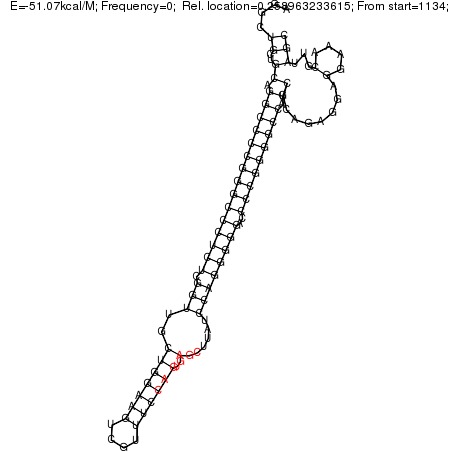

Supplement: Additional file 3 — Figure S2. Secondary structures for sequence region 0 to 0.3 of full length for the selected 32 eRNAs (see Fig. 7). [file 1471-2164-12-S3-S18-S3.zip › Figure S2/Centroid/CAGUGAGC_rank-1_12698046.jpg]

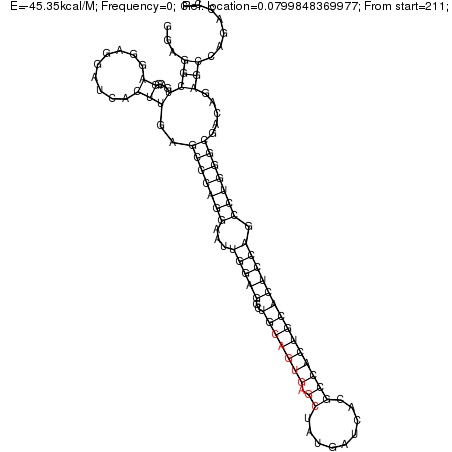

Supplement: Additional file 3 — Figure S2. Secondary structures for sequence region 0 to 0.3 of full length for the selected 32 eRNAs (see Fig. 7). [file 1471-2164-12-S3-S18-S3.zip › Figure S2/Centroid/CAGUGAGC_rank-2_85362714.jpg]

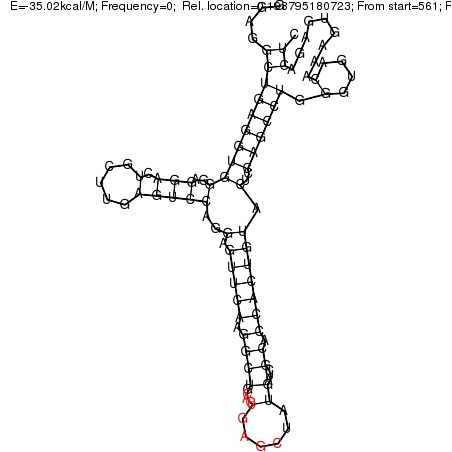

Supplement: Additional file 3 — Figure S2. Secondary structures for sequence region 0 to 0.3 of full length for the selected 32 eRNAs (see Fig. 7). [file 1471-2164-12-S3-S18-S3.zip › Figure S2/Centroid/CAGUGAGC_rank-3_21758081.jpg]

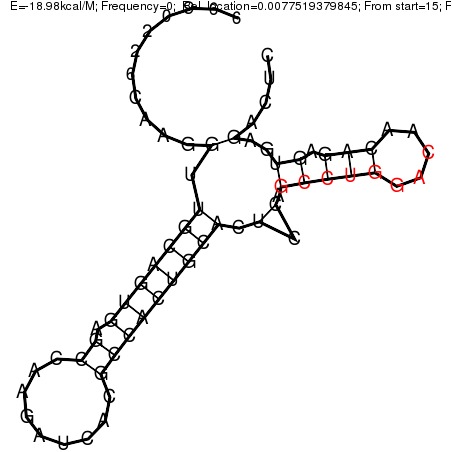

Supplement: Additional file 3 — Figure S2. Secondary structures for sequence region 0 to 0.3 of full length for the selected 32 eRNAs (see Fig. 7). [file 1471-2164-12-S3-S18-S3.zip › Figure S2/Centroid/CAGUGAGC_rank-4_6690226.jpg]

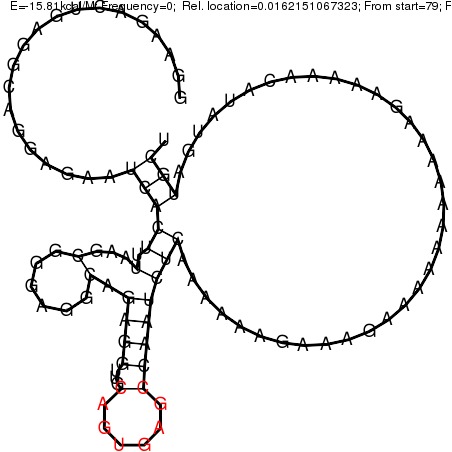

Supplement: Additional file 3 — Figure S2. Secondary structures for sequence region 0 to 0.3 of full length for the selected 32 eRNAs (see Fig. 7). [file 1471-2164-12-S3-S18-S3.zip › Figure S2/Centroid/CAGUGAGC_rank-5_71480116.jpg]

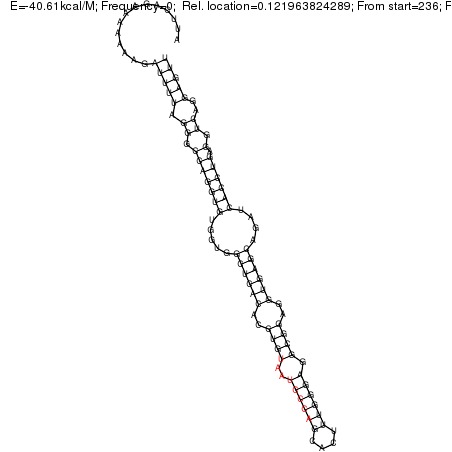

Supplement: Additional file 3 — Figure S2. Secondary structures for sequence region 0 to 0.3 of full length for the selected 32 eRNAs (see Fig. 7). [file 1471-2164-12-S3-S18-S3.zip › Figure S2/Centroid/UAAUCCCA_rank-1_6690226.jpg]

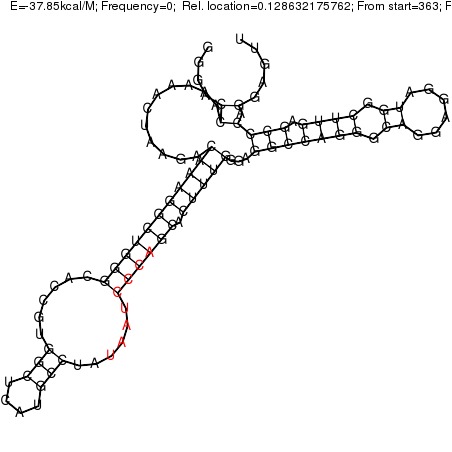

Supplement: Additional file 3 — Figure S2. Secondary structures for sequence region 0 to 0.3 of full length for the selected 32 eRNAs (see Fig. 7). [file 1471-2164-12-S3-S18-S3.zip › Figure S2/Centroid/UAAUCCCA_rank-2_21758081.jpg]

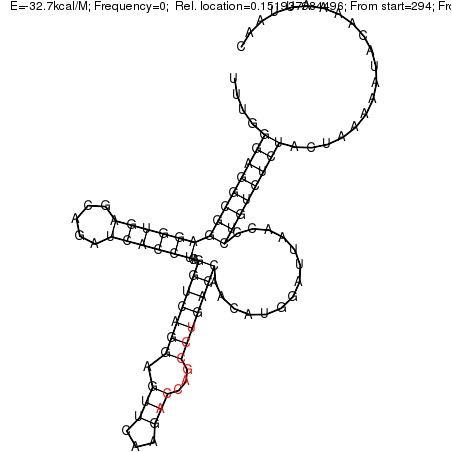

Supplement: Additional file 3 — Figure S2. Secondary structures for sequence region 0 to 0.3 of full length for the selected 32 eRNAs (see Fig. 7). [file 1471-2164-12-S3-S18-S3.zip › Figure S2/MFE/ACCAGCCU_rank-1_6690226.jpg]

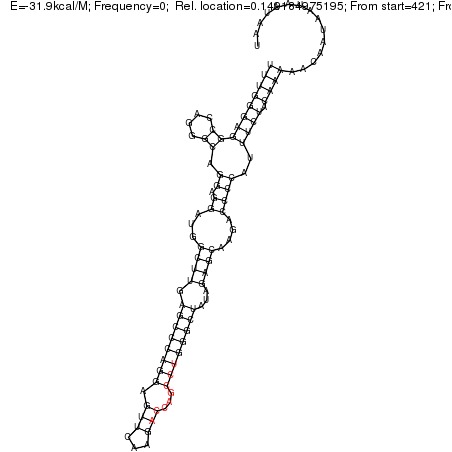

Supplement: Additional file 3 — Figure S2. Secondary structures for sequence region 0 to 0.3 of full length for the selected 32 eRNAs (see Fig. 7). [file 1471-2164-12-S3-S18-S3.zip › Figure S2/MFE/ACCAGCCU_rank-2_21758081.jpg]

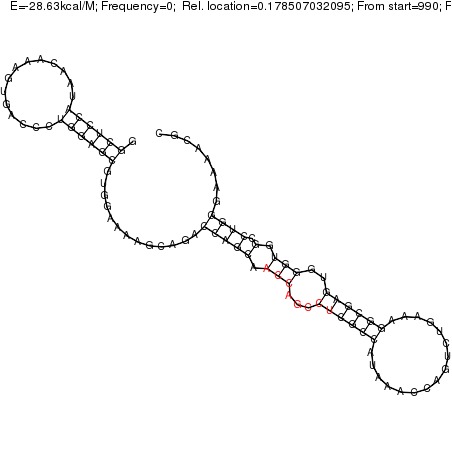

Supplement: Additional file 3 — Figure S2. Secondary structures for sequence region 0 to 0.3 of full length for the selected 32 eRNAs (see Fig. 7). [file 1471-2164-12-S3-S18-S3.zip › Figure S2/MFE/ACCAGCCU_rank-3_10436764.jpg]

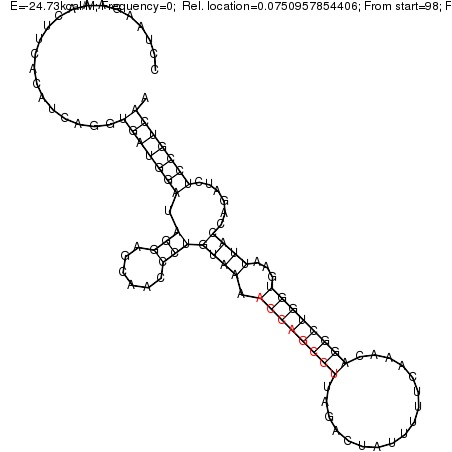

Supplement: Additional file 3 — Figure S2. Secondary structures for sequence region 0 to 0.3 of full length for the selected 32 eRNAs (see Fig. 7). [file 1471-2164-12-S3-S18-S3.zip › Figure S2/MFE/ACCAGCCU_rank-4_117320510.jpg]

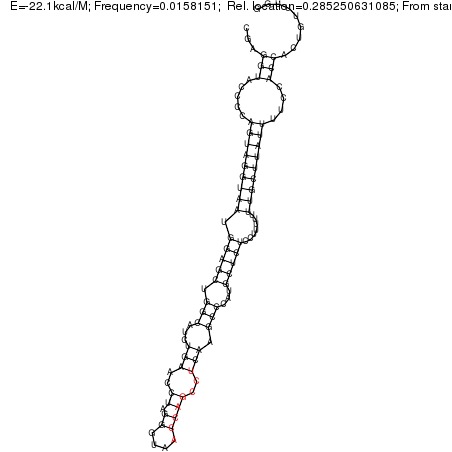

Supplement: Additional file 3 — Figure S2. Secondary structures for sequence region 0 to 0.3 of full length for the selected 32 eRNAs (see Fig. 7). [file 1471-2164-12-S3-S18-S3.zip › Figure S2/MFE/ACCAGCCU_rank-5_10436764.jpg]

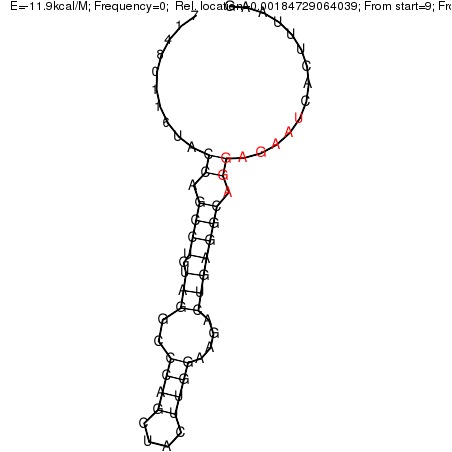

Supplement: Additional file 3 — Figure S2. Secondary structures for sequence region 0 to 0.3 of full length for the selected 32 eRNAs (see Fig. 7). [file 1471-2164-12-S3-S18-S3.zip › Figure S2/MFE/ACCAGCCU_rank-6_71480116.jpg]

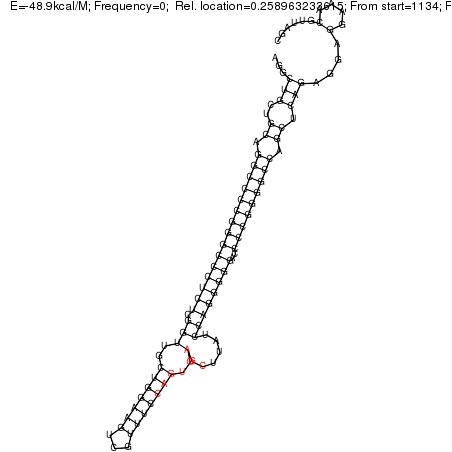

Supplement: Additional file 3 — Figure S2. Secondary structures for sequence region 0 to 0.3 of full length for the selected 32 eRNAs (see Fig. 7). [file 1471-2164-12-S3-S18-S3.zip › Figure S2/MFE/CAGUGAGC_rank-1_12698046.jpg]

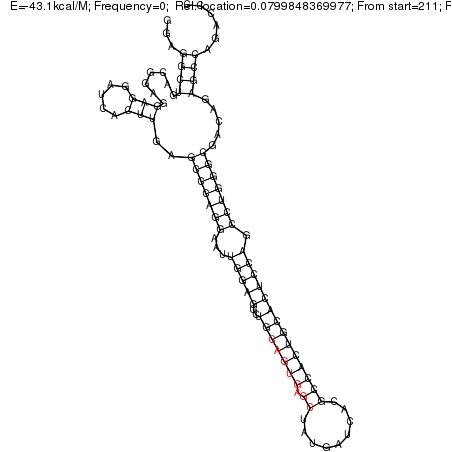

Supplement: Additional file 3 — Figure S2. Secondary structures for sequence region 0 to 0.3 of full length for the selected 32 eRNAs (see Fig. 7). [file 1471-2164-12-S3-S18-S3.zip › Figure S2/MFE/CAGUGAGC_rank-2_85362714.jpg]

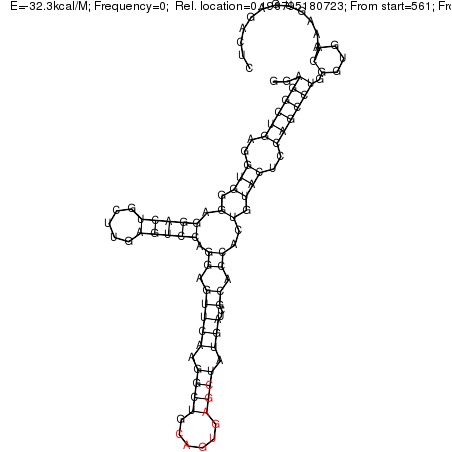

Supplement: Additional file 3 — Figure S2. Secondary structures for sequence region 0 to 0.3 of full length for the selected 32 eRNAs (see Fig. 7). [file 1471-2164-12-S3-S18-S3.zip › Figure S2/MFE/CAGUGAGC_rank-3_21758081.jpg]

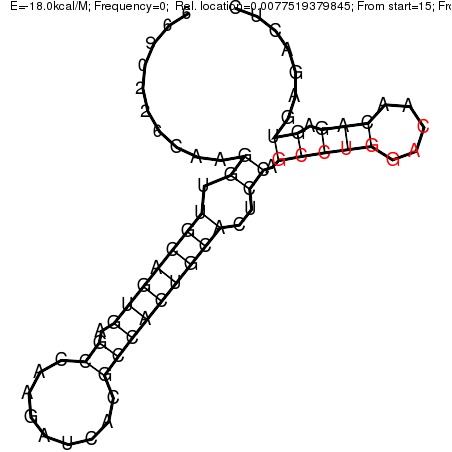

Supplement: Additional file 3 — Figure S2. Secondary structures for sequence region 0 to 0.3 of full length for the selected 32 eRNAs (see Fig. 7). [file 1471-2164-12-S3-S18-S3.zip › Figure S2/MFE/CAGUGAGC_rank-4_6690226.jpg]

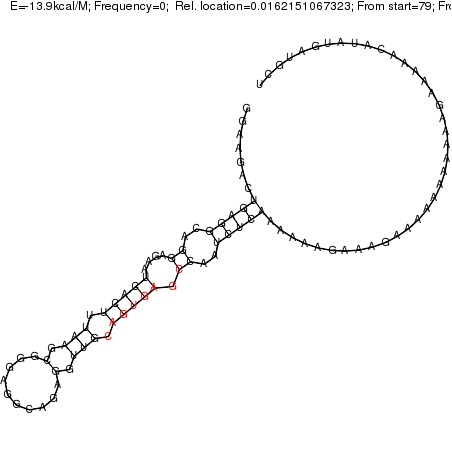

Supplement: Additional file 3 — Figure S2. Secondary structures for sequence region 0 to 0.3 of full length for the selected 32 eRNAs (see Fig. 7). [file 1471-2164-12-S3-S18-S3.zip › Figure S2/MFE/CAGUGAGC_rank-5_71480116.jpg]

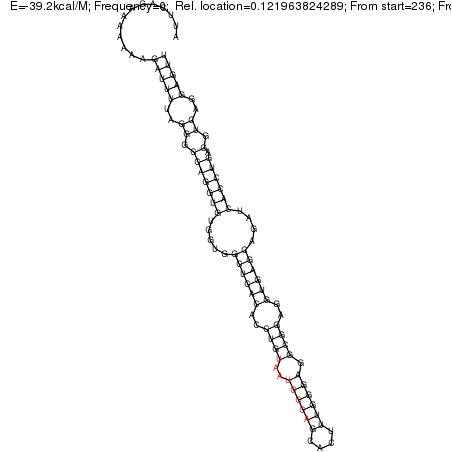

Supplement: Additional file 3 — Figure S2. Secondary structures for sequence region 0 to 0.3 of full length for the selected 32 eRNAs (see Fig. 7). [file 1471-2164-12-S3-S18-S3.zip › Figure S2/MFE/UAAUCCCA_rank-1_6690226.jpg]

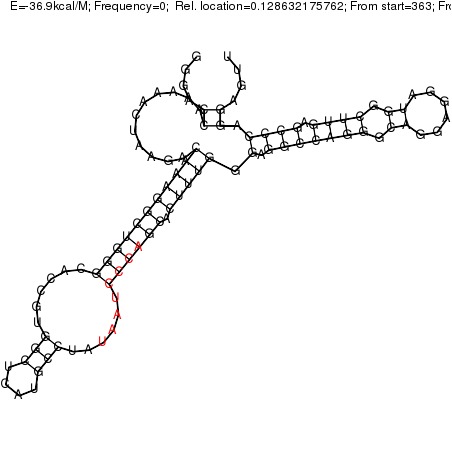

Supplement: Additional file 3 — Figure S2. Secondary structures for sequence region 0 to 0.3 of full length for the selected 32 eRNAs (see Fig. 7). [file 1471-2164-12-S3-S18-S3.zip › Figure S2/MFE/UAAUCCCA_rank-2_21758081.jpg]

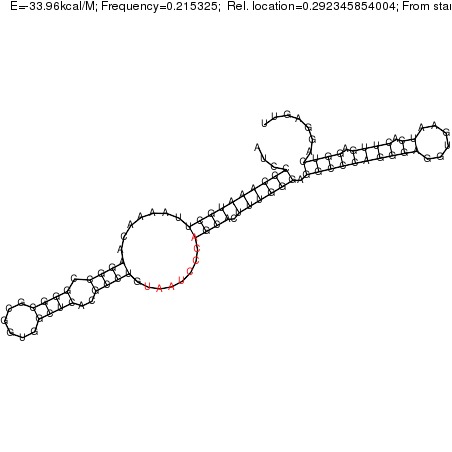

Supplement: Additional file 3 — Figure S2. Secondary structures for sequence region 0 to 0.3 of full length for the selected 32 eRNAs (see Fig. 7). [file 1471-2164-12-S3-S18-S3.zip › Figure S2/MFE/UAAUCCCA_rank-3_21758081.jpg]

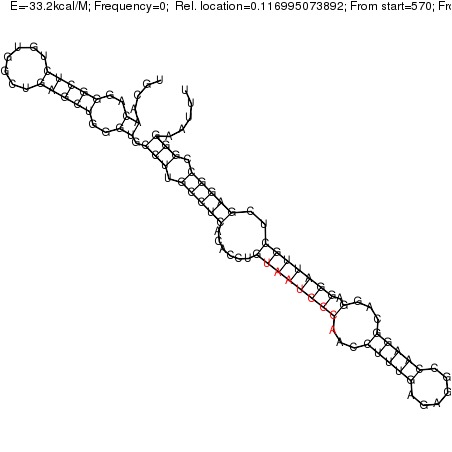

Supplement: Additional file 3 — Figure S2. Secondary structures for sequence region 0 to 0.3 of full length for the selected 32 eRNAs (see Fig. 7). [file 1471-2164-12-S3-S18-S3.zip › Figure S2/MFE/UAAUCCCA_rank-4_71480116.jpg]

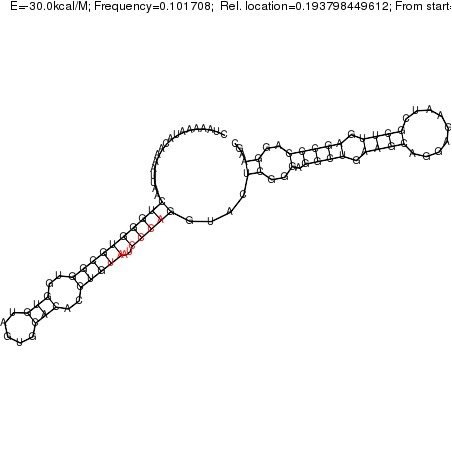

Supplement: Additional file 3 — Figure S2. Secondary structures for sequence region 0 to 0.3 of full length for the selected 32 eRNAs (see Fig. 7). [file 1471-2164-12-S3-S18-S3.zip › Figure S2/MFE/UAAUCCCA_rank-5_6690226.jpg]

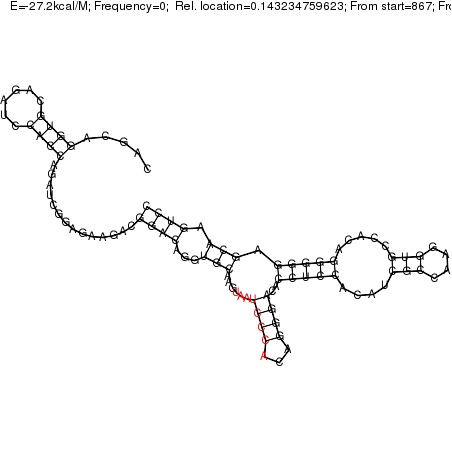

Supplement: Additional file 3 — Figure S2. Secondary structures for sequence region 0 to 0.3 of full length for the selected 32 eRNAs (see Fig. 7). [file 1471-2164-12-S3-S18-S3.zip › Figure S2/MFE/UAAUCCCA_rank-6_10435879.jpg]

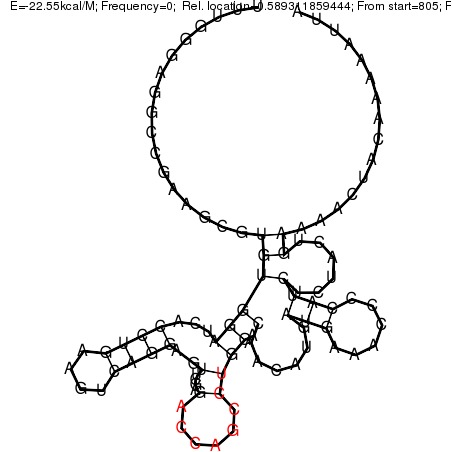

Supplement: Additional file 4 — Figure S3. Secondary structures for sequence region 0.3 to 0.7 of full length for the selected 32 eRNAs (see Fig. 7). [file 1471-2164-12-S3-S18-S4.zip › Figure S3/Centroid/ACCAGCCU_rank-10_10439148.jpg]

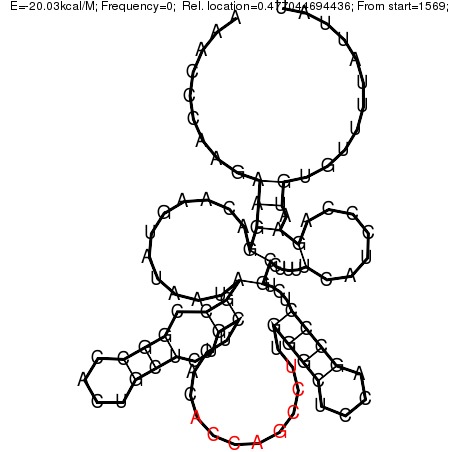

Supplement: Additional file 4 — Figure S3. Secondary structures for sequence region 0.3 to 0.7 of full length for the selected 32 eRNAs (see Fig. 7). [file 1471-2164-12-S3-S18-S4.zip › Figure S3/Centroid/ACCAGCCU_rank-11_92859582.jpg]

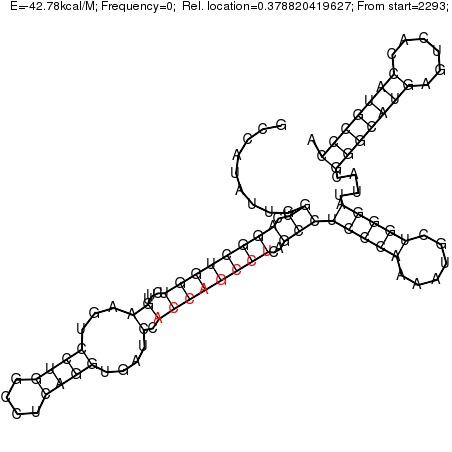

Supplement: Additional file 4 — Figure S3. Secondary structures for sequence region 0.3 to 0.7 of full length for the selected 32 eRNAs (see Fig. 7). [file 1471-2164-12-S3-S18-S4.zip › Figure S3/Centroid/ACCAGCCU_rank-1_10435879.jpg]

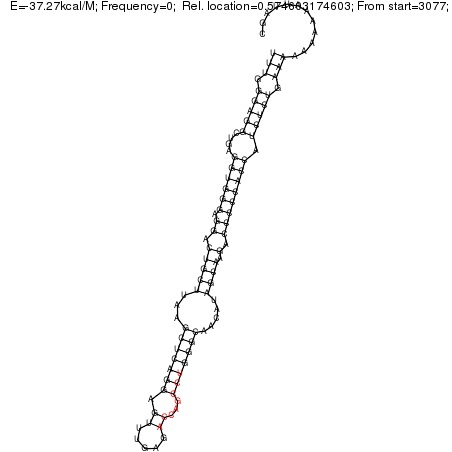

Supplement: Additional file 4 — Figure S3. Secondary structures for sequence region 0.3 to 0.7 of full length for the selected 32 eRNAs (see Fig. 7). [file 1471-2164-12-S3-S18-S4.zip › Figure S3/Centroid/ACCAGCCU_rank-2_149363691.jpg]

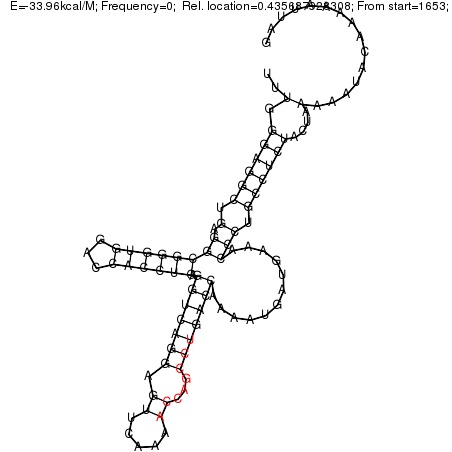

Supplement: Additional file 4 — Figure S3. Secondary structures for sequence region 0.3 to 0.7 of full length for the selected 32 eRNAs (see Fig. 7). [file 1471-2164-12-S3-S18-S4.zip › Figure S3/Centroid/ACCAGCCU_rank-3_153252197.jpg]

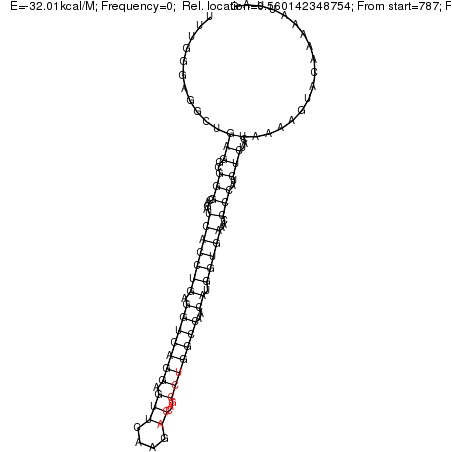

Supplement: Additional file 4 — Figure S3. Secondary structures for sequence region 0.3 to 0.7 of full length for the selected 32 eRNAs (see Fig. 7). [file 1471-2164-12-S3-S18-S4.zip › Figure S3/Centroid/ACCAGCCU_rank-6_10436915.jpg]

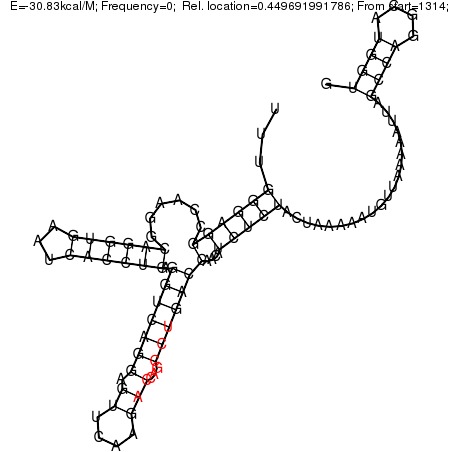

Supplement: Additional file 4 — Figure S3. Secondary structures for sequence region 0.3 to 0.7 of full length for the selected 32 eRNAs (see Fig. 7). [file 1471-2164-12-S3-S18-S4.zip › Figure S3/Centroid/ACCAGCCU_rank-7_34531080.jpg]

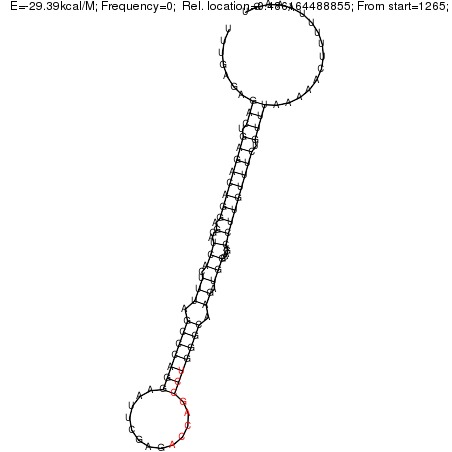

Supplement: Additional file 4 — Figure S3. Secondary structures for sequence region 0.3 to 0.7 of full length for the selected 32 eRNAs (see Fig. 7). [file 1471-2164-12-S3-S18-S4.zip › Figure S3/Centroid/ACCAGCCU_rank-8_16550670.jpg]

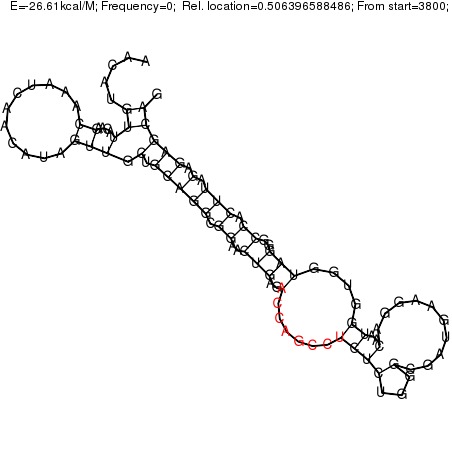

Supplement: Additional file 4 — Figure S3. Secondary structures for sequence region 0.3 to 0.7 of full length for the selected 32 eRNAs (see Fig. 7). [file 1471-2164-12-S3-S18-S4.zip › Figure S3/Centroid/ACCAGCCU_rank-9_156447020.jpg]

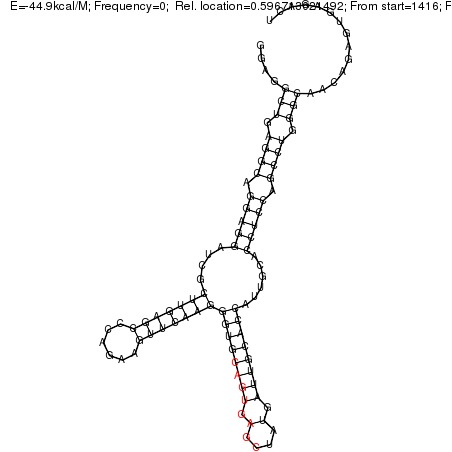

Supplement: Additional file 4 — Figure S3. Secondary structures for sequence region 0.3 to 0.7 of full length for the selected 32 eRNAs (see Fig. 7). [file 1471-2164-12-S3-S18-S4.zip › Figure S3/Centroid/CAGUGAGC_rank-1_10440286.jpg]

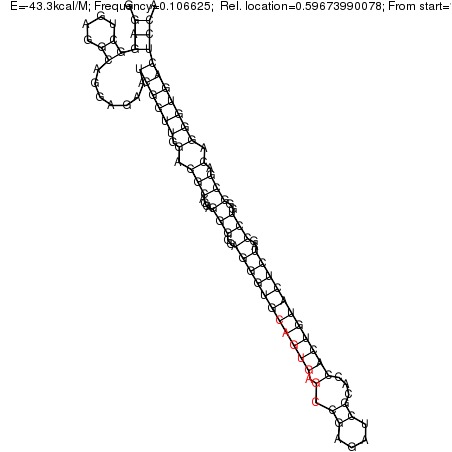

Supplement: Additional file 4 — Figure S3. Secondary structures for sequence region 0.3 to 0.7 of full length for the selected 32 eRNAs (see Fig. 7). [file 1471-2164-12-S3-S18-S4.zip › Figure S3/Centroid/CAGUGAGC_rank-2_21758081.jpg]

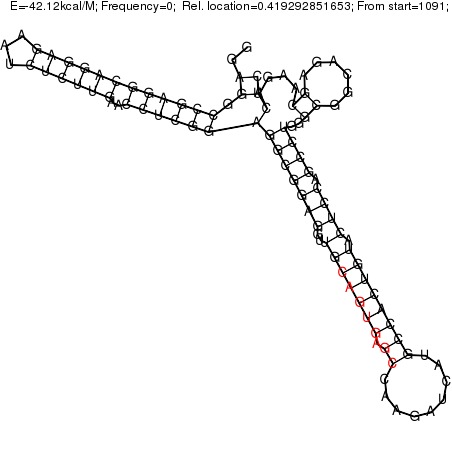

Supplement: Additional file 4 — Figure S3. Secondary structures for sequence region 0.3 to 0.7 of full length for the selected 32 eRNAs (see Fig. 7). [file 1471-2164-12-S3-S18-S4.zip › Figure S3/Centroid/CAGUGAGC_rank-3_16550670.jpg]

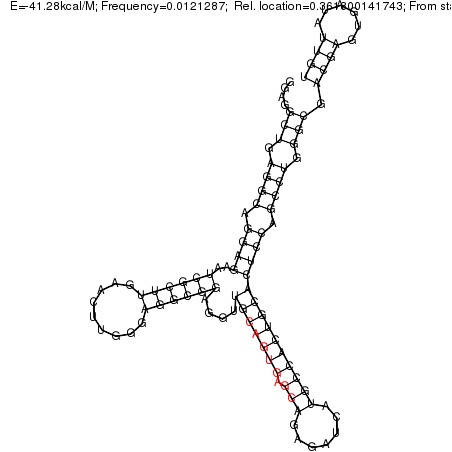

Supplement: Additional file 4 — Figure S3. Secondary structures for sequence region 0.3 to 0.7 of full length for the selected 32 eRNAs (see Fig. 7). [file 1471-2164-12-S3-S18-S4.zip › Figure S3/Centroid/CAGUGAGC_rank-4_21758081.jpg]

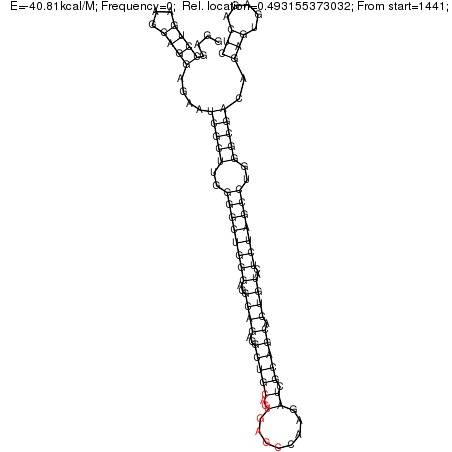

Supplement: Additional file 4 — Figure S3. Secondary structures for sequence region 0.3 to 0.7 of full length for the selected 32 eRNAs (see Fig. 7). [file 1471-2164-12-S3-S18-S4.zip › Figure S3/Centroid/CAGUGAGC_rank-5_34531080.jpg]

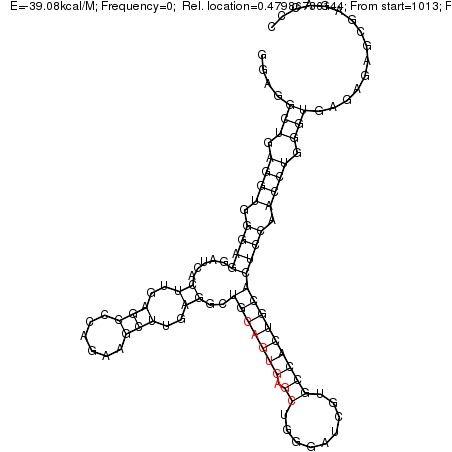

Supplement: Additional file 4 — Figure S3. Secondary structures for sequence region 0.3 to 0.7 of full length for the selected 32 eRNAs (see Fig. 7). [file 1471-2164-12-S3-S18-S4.zip › Figure S3/Centroid/CAGUGAGC_rank-6_34529621.jpg]

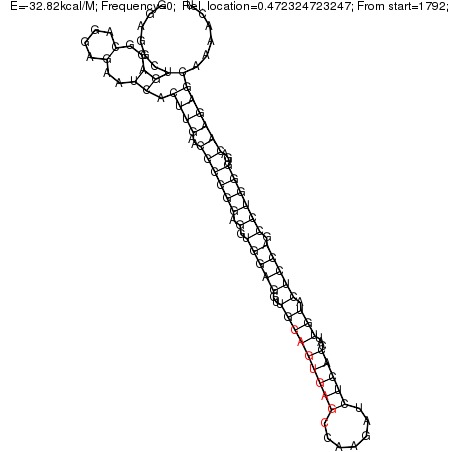

Supplement: Additional file 4 — Figure S3. Secondary structures for sequence region 0.3 to 0.7 of full length for the selected 32 eRNAs (see Fig. 7). [file 1471-2164-12-S3-S18-S4.zip › Figure S3/Centroid/CAGUGAGC_rank-7_153252197.jpg]

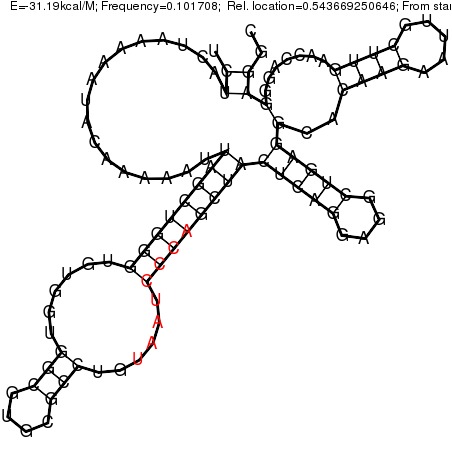

Supplement: Additional file 4 — Figure S3. Secondary structures for sequence region 0.3 to 0.7 of full length for the selected 32 eRNAs (see Fig. 7). [file 1471-2164-12-S3-S18-S4.zip › Figure S3/Centroid/UAAUCCCA_rank-10_6690226.jpg]

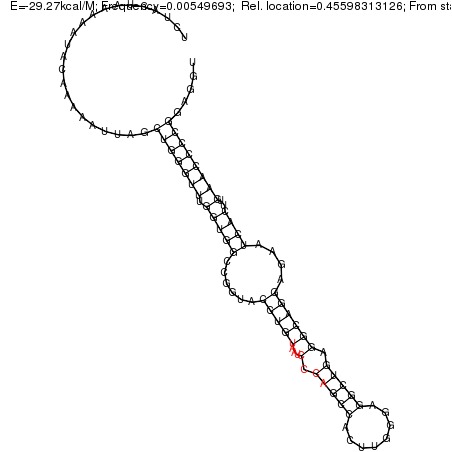

Supplement: Additional file 4 — Figure S3. Secondary structures for sequence region 0.3 to 0.7 of full length for the selected 32 eRNAs (see Fig. 7). [file 1471-2164-12-S3-S18-S4.zip › Figure S3/Centroid/UAAUCCCA_rank-11_153252197.jpg]

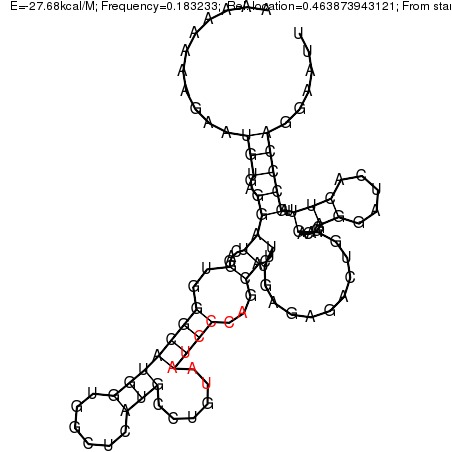

Supplement: Additional file 4 — Figure S3. Secondary structures for sequence region 0.3 to 0.7 of full length for the selected 32 eRNAs (see Fig. 7). [file 1471-2164-12-S3-S18-S4.zip › Figure S3/Centroid/UAAUCCCA_rank-12_16550670.jpg]

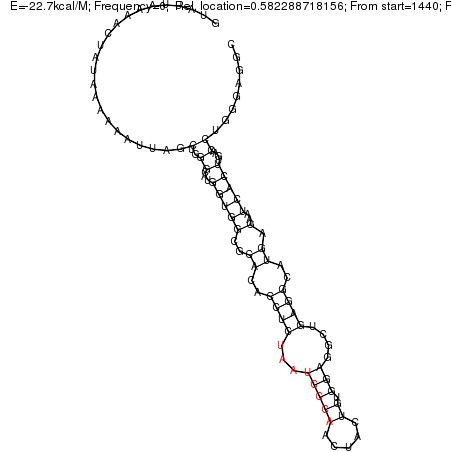

Supplement: Additional file 4 — Figure S3. Secondary structures for sequence region 0.3 to 0.7 of full length for the selected 32 eRNAs (see Fig. 7). [file 1471-2164-12-S3-S18-S4.zip › Figure S3/Centroid/UAAUCCCA_rank-13_10432841.jpg]

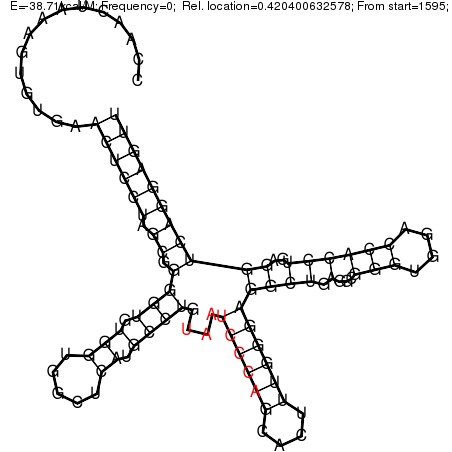

Supplement: Additional file 4 — Figure S3. Secondary structures for sequence region 0.3 to 0.7 of full length for the selected 32 eRNAs (see Fig. 7). [file 1471-2164-12-S3-S18-S4.zip › Figure S3/Centroid/UAAUCCCA_rank-1_153252197.jpg]

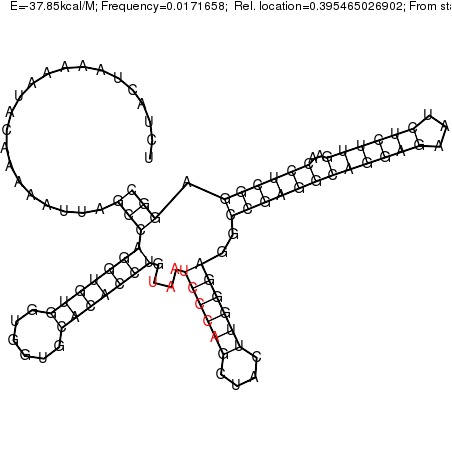

Supplement: Additional file 4 — Figure S3. Secondary structures for sequence region 0.3 to 0.7 of full length for the selected 32 eRNAs (see Fig. 7). [file 1471-2164-12-S3-S18-S4.zip › Figure S3/Centroid/UAAUCCCA_rank-2_16550670.jpg]

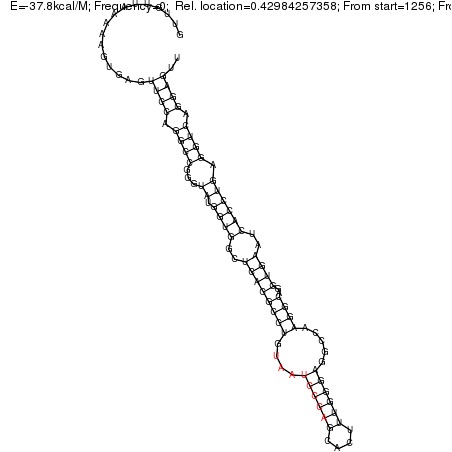

Supplement: Additional file 4 — Figure S3. Secondary structures for sequence region 0.3 to 0.7 of full length for the selected 32 eRNAs (see Fig. 7). [file 1471-2164-12-S3-S18-S4.zip › Figure S3/Centroid/UAAUCCCA_rank-3_34531080.jpg]

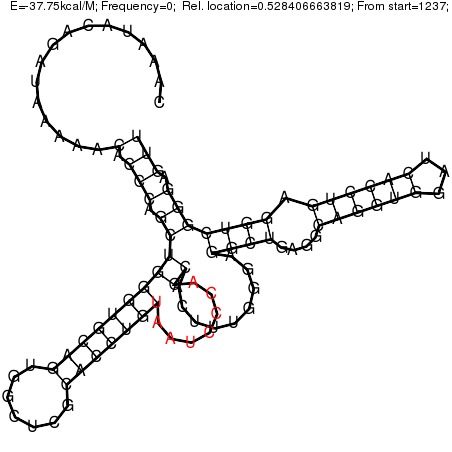

Supplement: Additional file 4 — Figure S3. Secondary structures for sequence region 0.3 to 0.7 of full length for the selected 32 eRNAs (see Fig. 7). [file 1471-2164-12-S3-S18-S4.zip › Figure S3/Centroid/UAAUCCCA_rank-4_7023439.jpg]

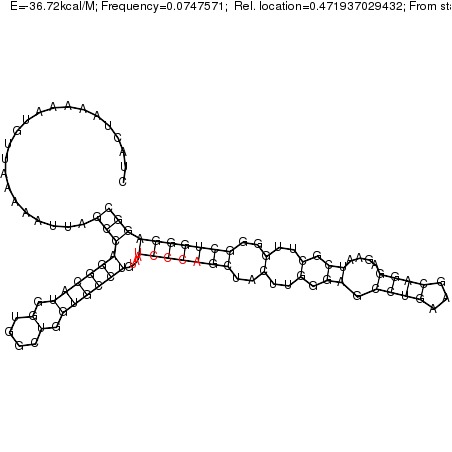

Supplement: Additional file 4 — Figure S3. Secondary structures for sequence region 0.3 to 0.7 of full length for the selected 32 eRNAs (see Fig. 7). [file 1471-2164-12-S3-S18-S4.zip › Figure S3/Centroid/UAAUCCCA_rank-5_34531080.jpg]

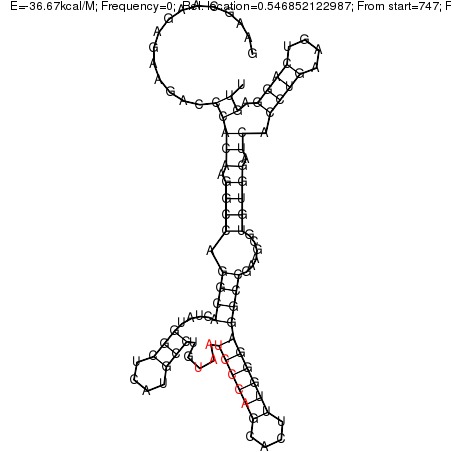

Supplement: Additional file 4 — Figure S3. Secondary structures for sequence region 0.3 to 0.7 of full length for the selected 32 eRNAs (see Fig. 7). [file 1471-2164-12-S3-S18-S4.zip › Figure S3/Centroid/UAAUCCCA_rank-6_10439148.jpg]

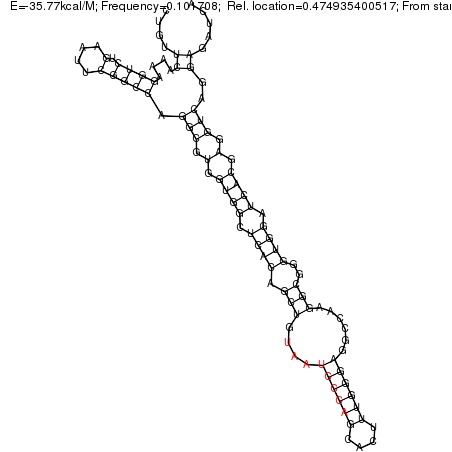

Supplement: Additional file 4 — Figure S3. Secondary structures for sequence region 0.3 to 0.7 of full length for the selected 32 eRNAs (see Fig. 7). [file 1471-2164-12-S3-S18-S4.zip › Figure S3/Centroid/UAAUCCCA_rank-7_6690226.jpg]

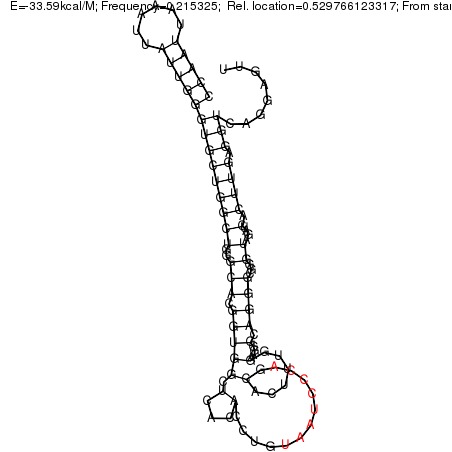

Supplement: Additional file 4 — Figure S3. Secondary structures for sequence region 0.3 to 0.7 of full length for the selected 32 eRNAs (see Fig. 7). [file 1471-2164-12-S3-S18-S4.zip › Figure S3/Centroid/UAAUCCCA_rank-8_21758081.jpg]

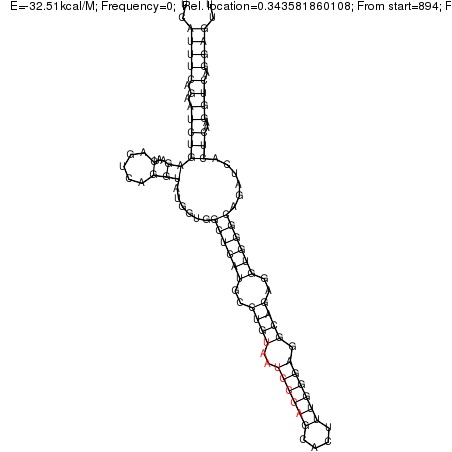

Supplement: Additional file 4 — Figure S3. Secondary structures for sequence region 0.3 to 0.7 of full length for the selected 32 eRNAs (see Fig. 7). [file 1471-2164-12-S3-S18-S4.zip › Figure S3/Centroid/UAAUCCCA_rank-9_16550670.jpg]

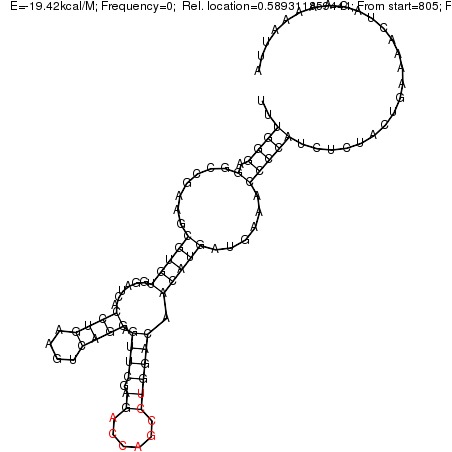

Supplement: Additional file 4 — Figure S3. Secondary structures for sequence region 0.3 to 0.7 of full length for the selected 32 eRNAs (see Fig. 7). [file 1471-2164-12-S3-S18-S4.zip › Figure S3/MFE/ACCAGCCU_rank-10_10439148.jpg]

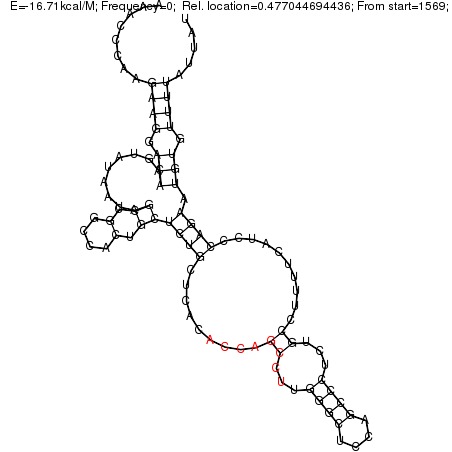

Supplement: Additional file 4 — Figure S3. Secondary structures for sequence region 0.3 to 0.7 of full length for the selected 32 eRNAs (see Fig. 7). [file 1471-2164-12-S3-S18-S4.zip › Figure S3/MFE/ACCAGCCU_rank-11_92859582.jpg]

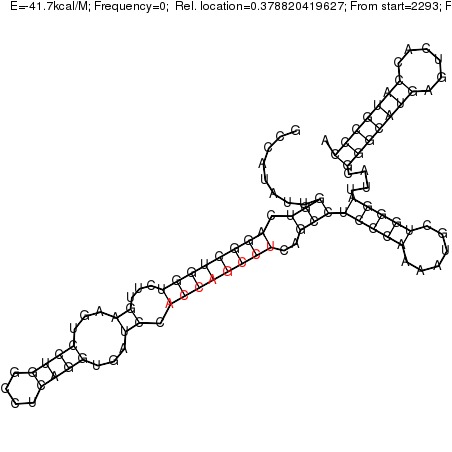

Supplement: Additional file 4 — Figure S3. Secondary structures for sequence region 0.3 to 0.7 of full length for the selected 32 eRNAs (see Fig. 7). [file 1471-2164-12-S3-S18-S4.zip › Figure S3/MFE/ACCAGCCU_rank-1_10435879.jpg]

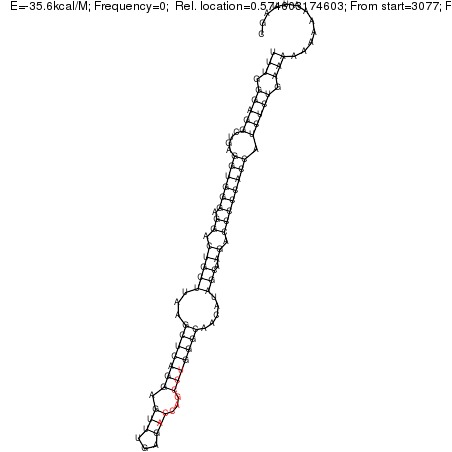

Supplement: Additional file 4 — Figure S3. Secondary structures for sequence region 0.3 to 0.7 of full length for the selected 32 eRNAs (see Fig. 7). [file 1471-2164-12-S3-S18-S4.zip › Figure S3/MFE/ACCAGCCU_rank-2_149363691.jpg]

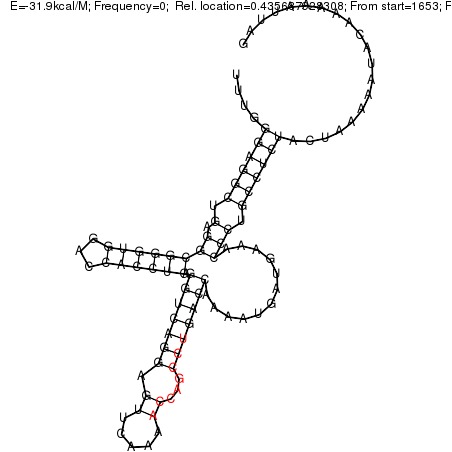

Supplement: Additional file 4 — Figure S3. Secondary structures for sequence region 0.3 to 0.7 of full length for the selected 32 eRNAs (see Fig. 7). [file 1471-2164-12-S3-S18-S4.zip › Figure S3/MFE/ACCAGCCU_rank-3_153252197.jpg]

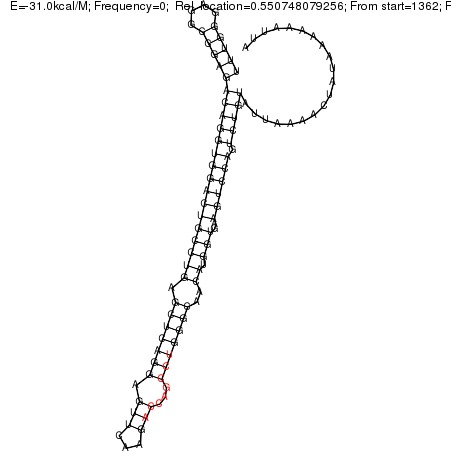

Supplement: Additional file 4 — Figure S3. Secondary structures for sequence region 0.3 to 0.7 of full length for the selected 32 eRNAs (see Fig. 7). [file 1471-2164-12-S3-S18-S4.zip › Figure S3/MFE/ACCAGCCU_rank-4_10432841.jpg]

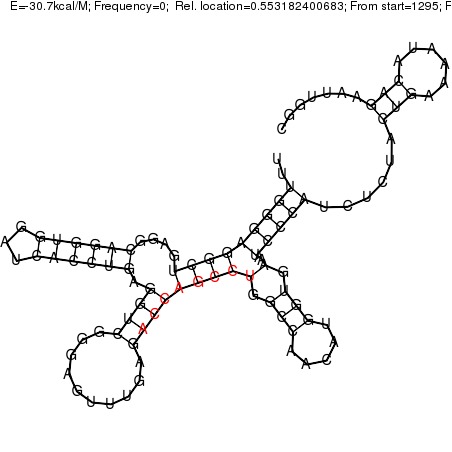

Supplement: Additional file 4 — Figure S3. Secondary structures for sequence region 0.3 to 0.7 of full length for the selected 32 eRNAs (see Fig. 7). [file 1471-2164-12-S3-S18-S4.zip › Figure S3/MFE/ACCAGCCU_rank-5_7023439.jpg]

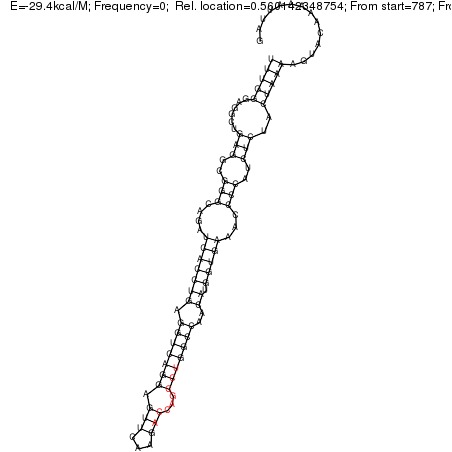

Supplement: Additional file 4 — Figure S3. Secondary structures for sequence region 0.3 to 0.7 of full length for the selected 32 eRNAs (see Fig. 7). [file 1471-2164-12-S3-S18-S4.zip › Figure S3/MFE/ACCAGCCU_rank-6_10436915.jpg]

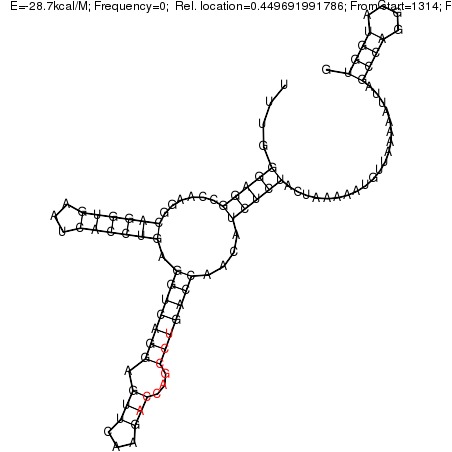

Supplement: Additional file 4 — Figure S3. Secondary structures for sequence region 0.3 to 0.7 of full length for the selected 32 eRNAs (see Fig. 7). [file 1471-2164-12-S3-S18-S4.zip › Figure S3/MFE/ACCAGCCU_rank-7_34531080.jpg]

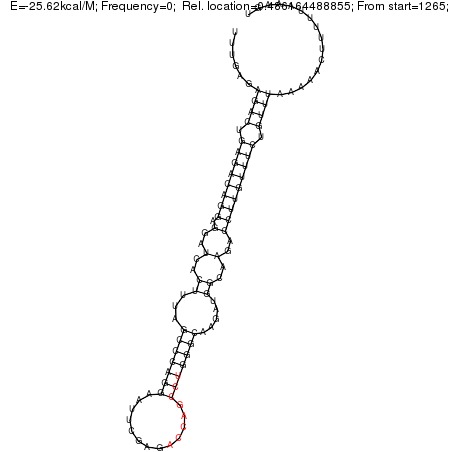

Supplement: Additional file 4 — Figure S3. Secondary structures for sequence region 0.3 to 0.7 of full length for the selected 32 eRNAs (see Fig. 7). [file 1471-2164-12-S3-S18-S4.zip › Figure S3/MFE/ACCAGCCU_rank-8_16550670.jpg]

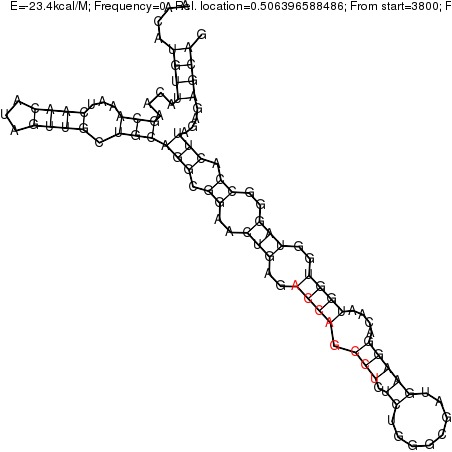

Supplement: Additional file 4 — Figure S3. Secondary structures for sequence region 0.3 to 0.7 of full length for the selected 32 eRNAs (see Fig. 7). [file 1471-2164-12-S3-S18-S4.zip › Figure S3/MFE/ACCAGCCU_rank-9_156447020.jpg]

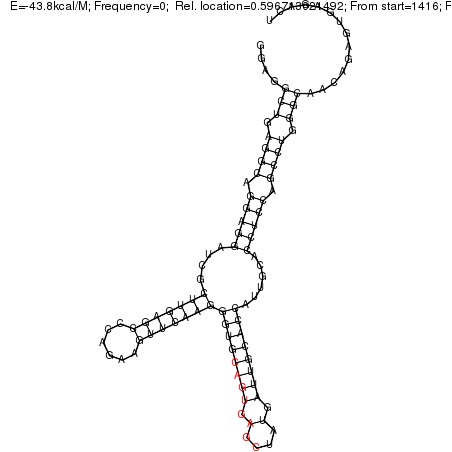

Supplement: Additional file 4 — Figure S3. Secondary structures for sequence region 0.3 to 0.7 of full length for the selected 32 eRNAs (see Fig. 7). [file 1471-2164-12-S3-S18-S4.zip › Figure S3/MFE/CAGUGAGC_rank-1_10440286.jpg]

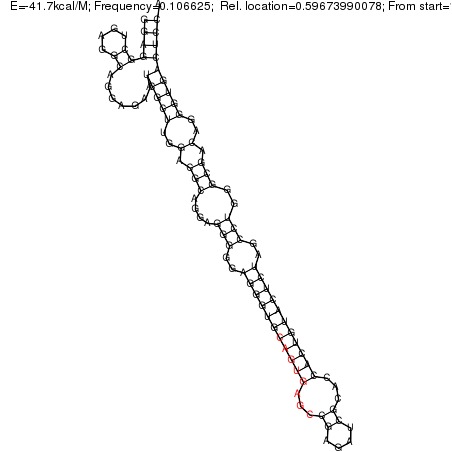

Supplement: Additional file 4 — Figure S3. Secondary structures for sequence region 0.3 to 0.7 of full length for the selected 32 eRNAs (see Fig. 7). [file 1471-2164-12-S3-S18-S4.zip › Figure S3/MFE/CAGUGAGC_rank-2_21758081.jpg]

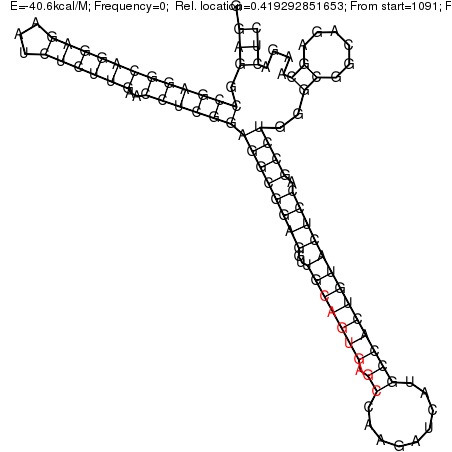

Supplement: Additional file 4 — Figure S3. Secondary structures for sequence region 0.3 to 0.7 of full length for the selected 32 eRNAs (see Fig. 7). [file 1471-2164-12-S3-S18-S4.zip › Figure S3/MFE/CAGUGAGC_rank-3_16550670.jpg]

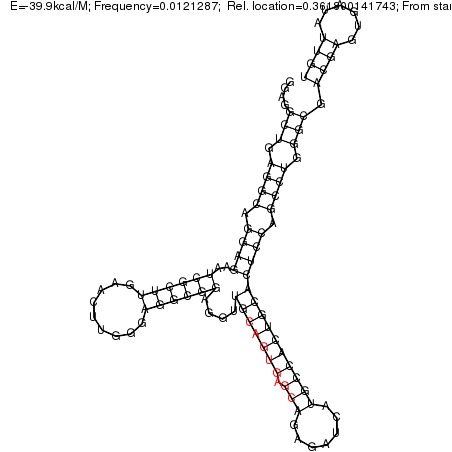

Supplement: Additional file 4 — Figure S3. Secondary structures for sequence region 0.3 to 0.7 of full length for the selected 32 eRNAs (see Fig. 7). [file 1471-2164-12-S3-S18-S4.zip › Figure S3/MFE/CAGUGAGC_rank-4_21758081.jpg]

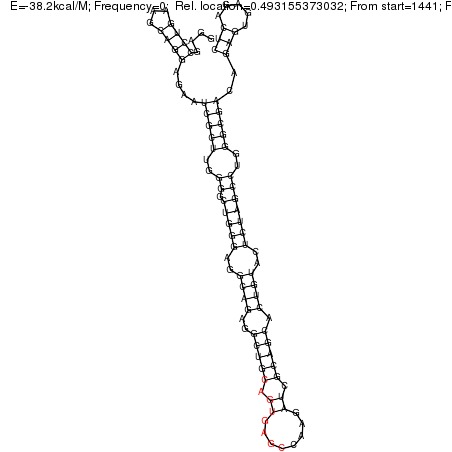

Supplement: Additional file 4 — Figure S3. Secondary structures for sequence region 0.3 to 0.7 of full length for the selected 32 eRNAs (see Fig. 7). [file 1471-2164-12-S3-S18-S4.zip › Figure S3/MFE/CAGUGAGC_rank-5_34531080.jpg]

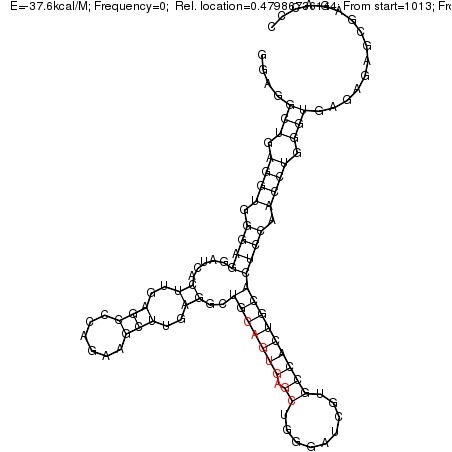

Supplement: Additional file 4 — Figure S3. Secondary structures for sequence region 0.3 to 0.7 of full length for the selected 32 eRNAs (see Fig. 7). [file 1471-2164-12-S3-S18-S4.zip › Figure S3/MFE/CAGUGAGC_rank-6_34529621.jpg]

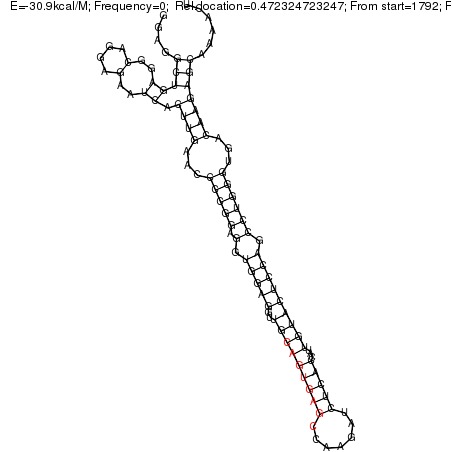

Supplement: Additional file 4 — Figure S3. Secondary structures for sequence region 0.3 to 0.7 of full length for the selected 32 eRNAs (see Fig. 7). [file 1471-2164-12-S3-S18-S4.zip › Figure S3/MFE/CAGUGAGC_rank-7_153252197.jpg]

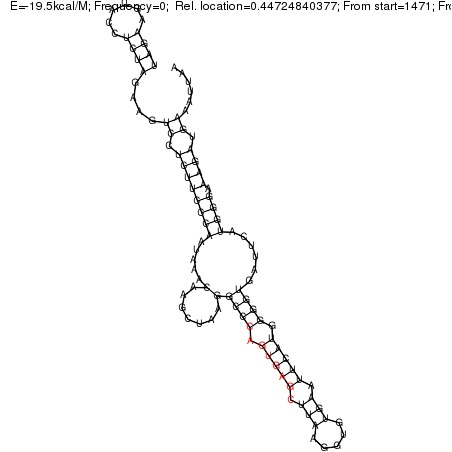

Supplement: Additional file 4 — Figure S3. Secondary structures for sequence region 0.3 to 0.7 of full length for the selected 32 eRNAs (see Fig. 7). [file 1471-2164-12-S3-S18-S4.zip › Figure S3/MFE/CAGUGAGC_rank-8_92859582.jpg]

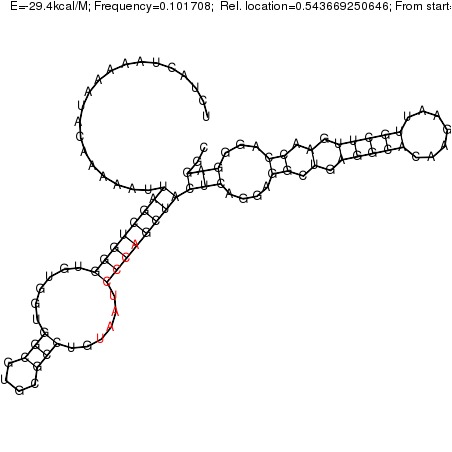

Supplement: Additional file 4 — Figure S3. Secondary structures for sequence region 0.3 to 0.7 of full length for the selected 32 eRNAs (see Fig. 7). [file 1471-2164-12-S3-S18-S4.zip › Figure S3/MFE/UAAUCCCA_rank-10_6690226.jpg]

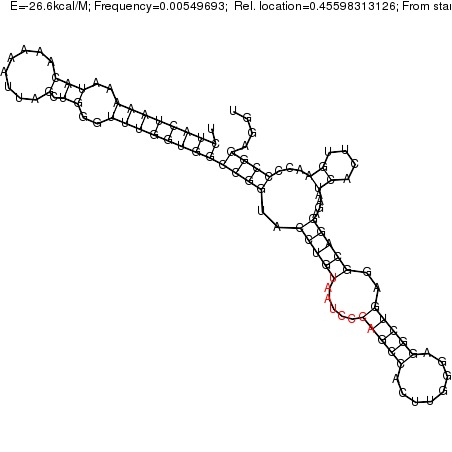

Supplement: Additional file 4 — Figure S3. Secondary structures for sequence region 0.3 to 0.7 of full length for the selected 32 eRNAs (see Fig. 7). [file 1471-2164-12-S3-S18-S4.zip › Figure S3/MFE/UAAUCCCA_rank-11_153252197.jpg]

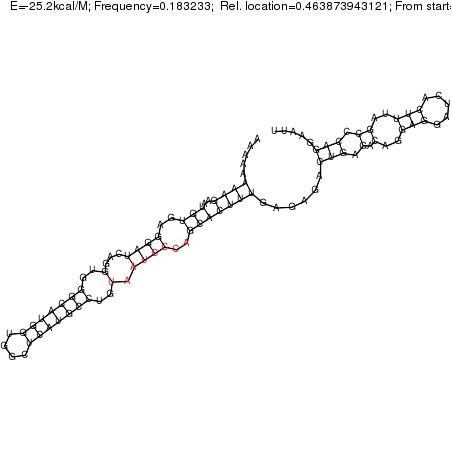

Supplement: Additional file 4 — Figure S3. Secondary structures for sequence region 0.3 to 0.7 of full length for the selected 32 eRNAs (see Fig. 7). [file 1471-2164-12-S3-S18-S4.zip › Figure S3/MFE/UAAUCCCA_rank-12_16550670.jpg]

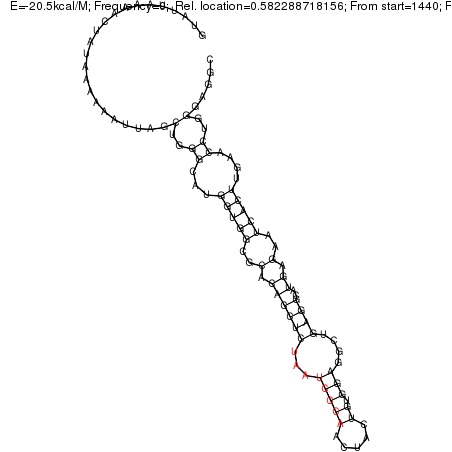

Supplement: Additional file 4 — Figure S3. Secondary structures for sequence region 0.3 to 0.7 of full length for the selected 32 eRNAs (see Fig. 7). [file 1471-2164-12-S3-S18-S4.zip › Figure S3/MFE/UAAUCCCA_rank-13_10432841.jpg]

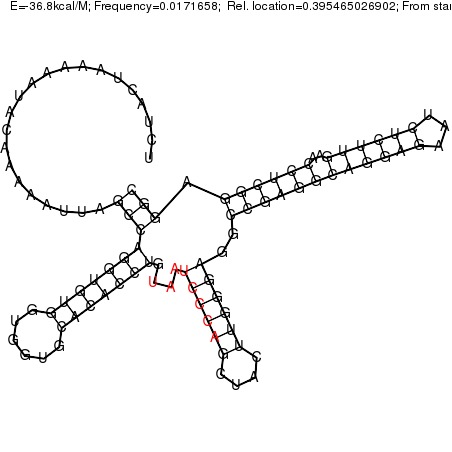

Supplement: Additional file 4 — Figure S3. Secondary structures for sequence region 0.3 to 0.7 of full length for the selected 32 eRNAs (see Fig. 7). [file 1471-2164-12-S3-S18-S4.zip › Figure S3/MFE/UAAUCCCA_rank-1_16550670.jpg]

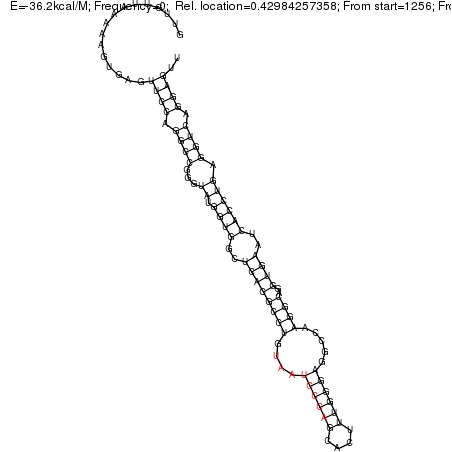

Supplement: Additional file 4 — Figure S3. Secondary structures for sequence region 0.3 to 0.7 of full length for the selected 32 eRNAs (see Fig. 7). [file 1471-2164-12-S3-S18-S4.zip › Figure S3/MFE/UAAUCCCA_rank-2_34531080.jpg]

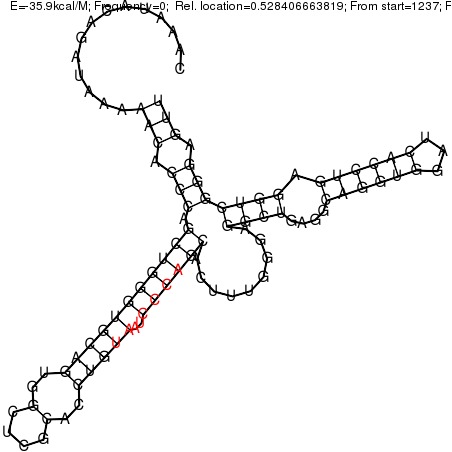

Supplement: Additional file 4 — Figure S3. Secondary structures for sequence region 0.3 to 0.7 of full length for the selected 32 eRNAs (see Fig. 7). [file 1471-2164-12-S3-S18-S4.zip › Figure S3/MFE/UAAUCCCA_rank-3_7023439.jpg]

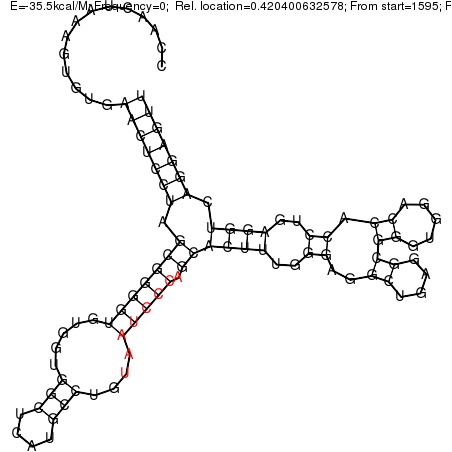

Supplement: Additional file 4 — Figure S3. Secondary structures for sequence region 0.3 to 0.7 of full length for the selected 32 eRNAs (see Fig. 7). [file 1471-2164-12-S3-S18-S4.zip › Figure S3/MFE/UAAUCCCA_rank-4_153252197.jpg]

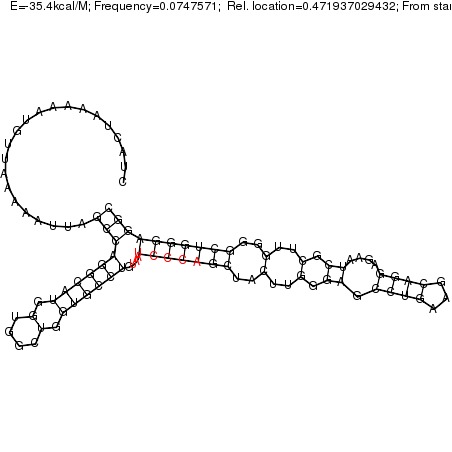

Supplement: Additional file 4 — Figure S3. Secondary structures for sequence region 0.3 to 0.7 of full length for the selected 32 eRNAs (see Fig. 7). [file 1471-2164-12-S3-S18-S4.zip › Figure S3/MFE/UAAUCCCA_rank-5_34531080.jpg]

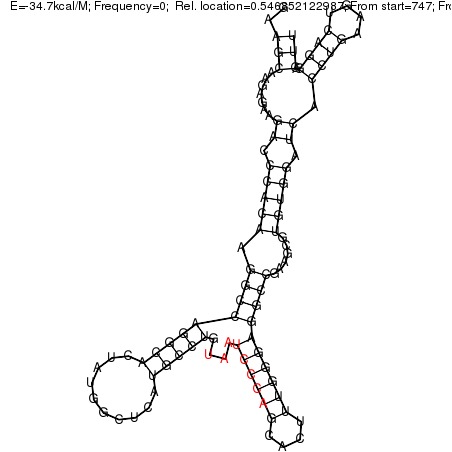

Supplement: Additional file 4 — Figure S3. Secondary structures for sequence region 0.3 to 0.7 of full length for the selected 32 eRNAs (see Fig. 7). [file 1471-2164-12-S3-S18-S4.zip › Figure S3/MFE/UAAUCCCA_rank-6_10439148.jpg]

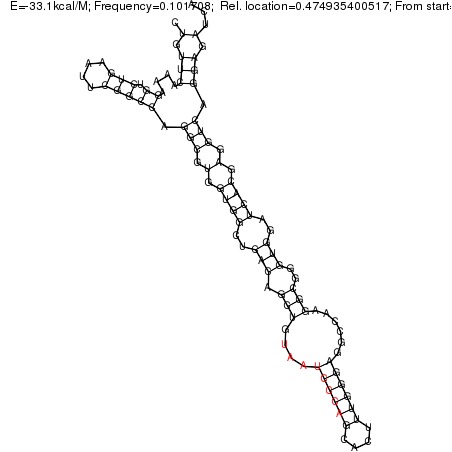

Supplement: Additional file 4 — Figure S3. Secondary structures for sequence region 0.3 to 0.7 of full length for the selected 32 eRNAs (see Fig. 7). [file 1471-2164-12-S3-S18-S4.zip › Figure S3/MFE/UAAUCCCA_rank-7_6690226.jpg]

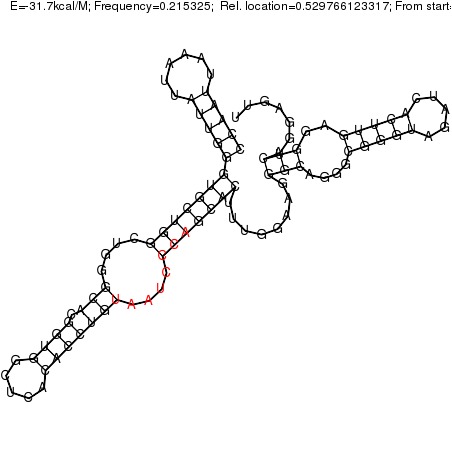

Supplement: Additional file 4 — Figure S3. Secondary structures for sequence region 0.3 to 0.7 of full length for the selected 32 eRNAs (see Fig. 7). [file 1471-2164-12-S3-S18-S4.zip › Figure S3/MFE/UAAUCCCA_rank-8_21758081.jpg]

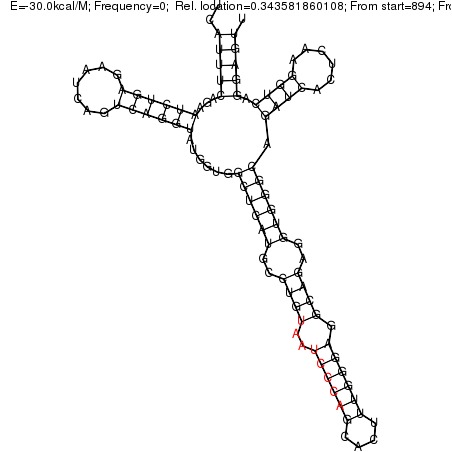

Supplement: Additional file 4 — Figure S3. Secondary structures for sequence region 0.3 to 0.7 of full length for the selected 32 eRNAs (see Fig. 7). [file 1471-2164-12-S3-S18-S4.zip › Figure S3/MFE/UAAUCCCA_rank-9_16550670.jpg]

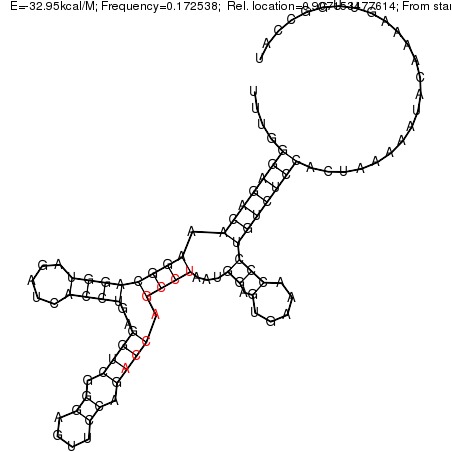

Supplement: Additional file 5 — Figure S4. Secondary structures for sequence region 0.7 to 1.0 of full length for the selected 32 eRNAs (see Fig. 7). [file 1471-2164-12-S3-S18-S5.zip › Figure S4/Centroid/ACCAGCCU_rank-10_10435879.jpg]

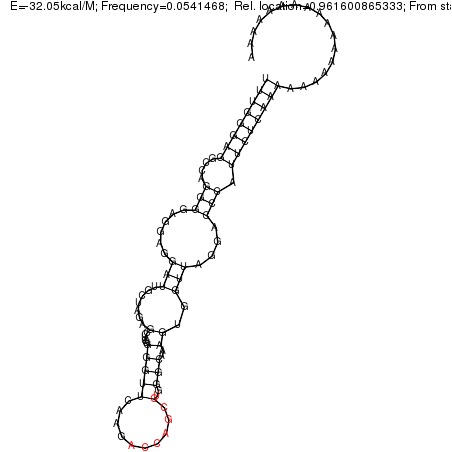

Supplement: Additional file 5 — Figure S4. Secondary structures for sequence region 0.7 to 1.0 of full length for the selected 32 eRNAs (see Fig. 7). [file 1471-2164-12-S3-S18-S5.zip › Figure S4/Centroid/ACCAGCCU_rank-11_10436389.jpg]

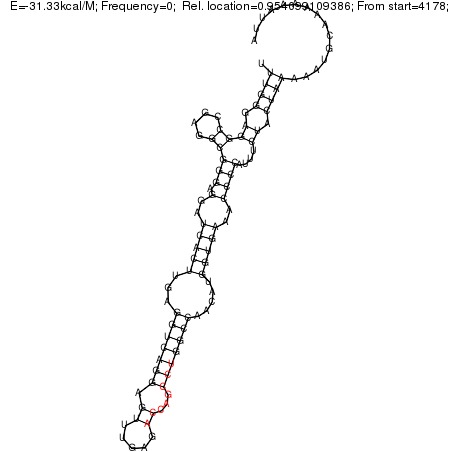

Supplement: Additional file 5 — Figure S4. Secondary structures for sequence region 0.7 to 1.0 of full length for the selected 32 eRNAs (see Fig. 7). [file 1471-2164-12-S3-S18-S5.zip › Figure S4/Centroid/ACCAGCCU_rank-12_12698046.jpg]

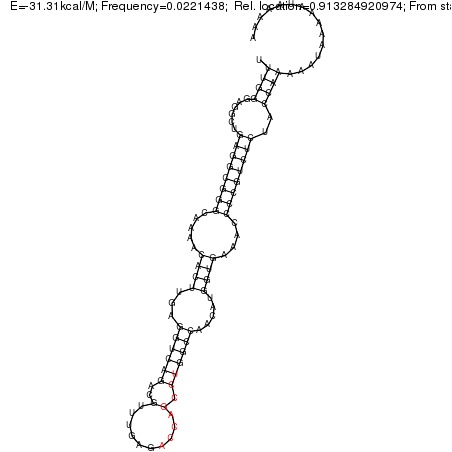

Supplement: Additional file 5 — Figure S4. Secondary structures for sequence region 0.7 to 1.0 of full length for the selected 32 eRNAs (see Fig. 7). [file 1471-2164-12-S3-S18-S5.zip › Figure S4/Centroid/ACCAGCCU_rank-13_7023439.jpg]

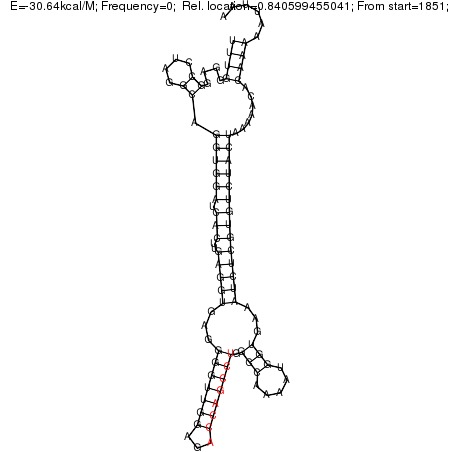

Supplement: Additional file 5 — Figure S4. Secondary structures for sequence region 0.7 to 1.0 of full length for the selected 32 eRNAs (see Fig. 7). [file 1471-2164-12-S3-S18-S5.zip › Figure S4/Centroid/ACCAGCCU_rank-14_37547428.jpg]

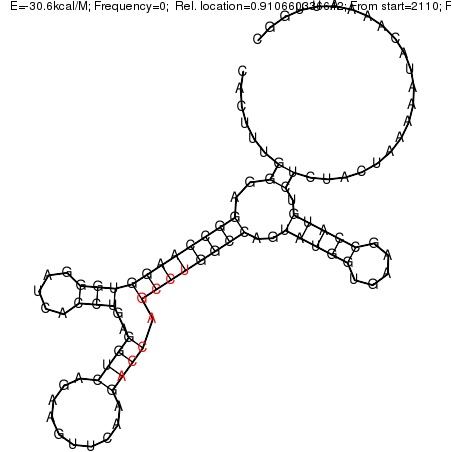

Supplement: Additional file 5 — Figure S4. Secondary structures for sequence region 0.7 to 1.0 of full length for the selected 32 eRNAs (see Fig. 7). [file 1471-2164-12-S3-S18-S5.zip › Figure S4/Centroid/ACCAGCCU_rank-15_30410976.jpg]

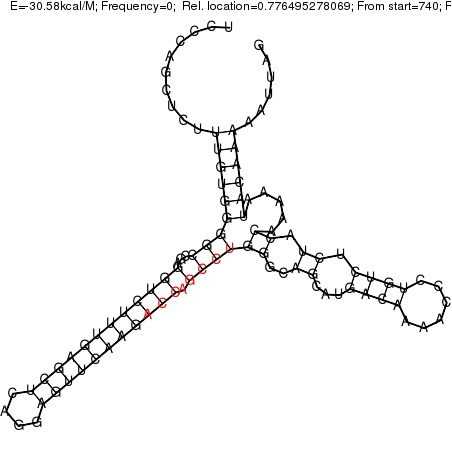

Supplement: Additional file 5 — Figure S4. Secondary structures for sequence region 0.7 to 1.0 of full length for the selected 32 eRNAs (see Fig. 7). [file 1471-2164-12-S3-S18-S5.zip › Figure S4/Centroid/ACCAGCCU_rank-16_4504692.jpg]

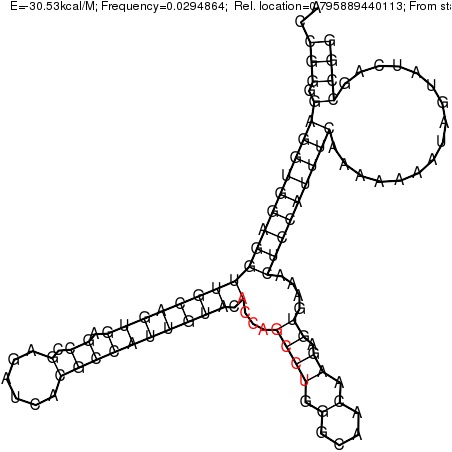

Supplement: Additional file 5 — Figure S4. Secondary structures for sequence region 0.7 to 1.0 of full length for the selected 32 eRNAs (see Fig. 7). [file 1471-2164-12-S3-S18-S5.zip › Figure S4/Centroid/ACCAGCCU_rank-17_21758081.jpg]

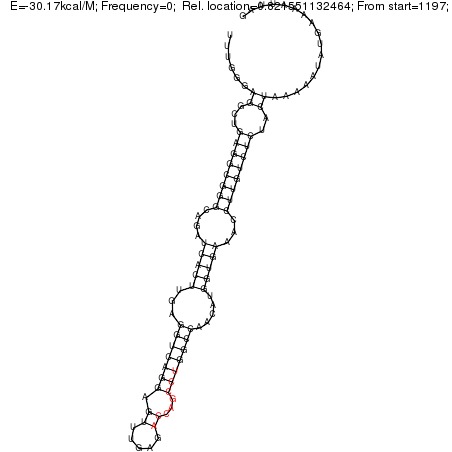

Supplement: Additional file 5 — Figure S4. Secondary structures for sequence region 0.7 to 1.0 of full length for the selected 32 eRNAs (see Fig. 7). [file 1471-2164-12-S3-S18-S5.zip › Figure S4/Centroid/ACCAGCCU_rank-18_32481159.jpg]

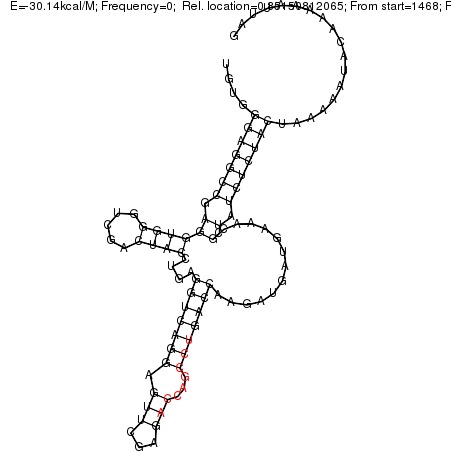

Supplement: Additional file 5 — Figure S4. Secondary structures for sequence region 0.7 to 1.0 of full length for the selected 32 eRNAs (see Fig. 7). [file 1471-2164-12-S3-S18-S5.zip › Figure S4/Centroid/ACCAGCCU_rank-19_45710101.jpg]

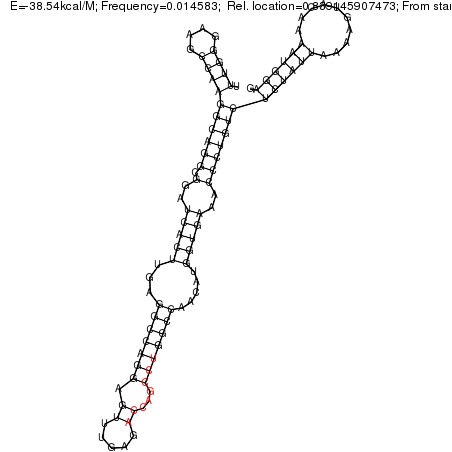

Supplement: Additional file 5 — Figure S4. Secondary structures for sequence region 0.7 to 1.0 of full length for the selected 32 eRNAs (see Fig. 7). [file 1471-2164-12-S3-S18-S5.zip › Figure S4/Centroid/ACCAGCCU_rank-1_10436915.jpg]
