# Supplementary figures and images for: Identification of nucleotide patterns enriched in secreted RNAs as putative cis-acting elements targeting them to exosome nano-vesicles (part 2 of 3)
Source: BMC Genomics. 2011 Nov 30;12(Suppl 3):S18. doi: 10.1186/1471-2164-12-S3-S18 (PMC3333177; doi:10.1186/1471-2164-12-S3-S18)

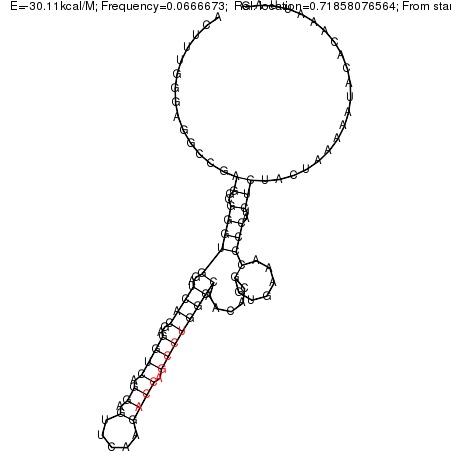

Supplement: Additional file 5 — Figure S4. Secondary structures for sequence region 0.7 to 1.0 of full length for the selected 32 eRNAs (see Fig. 7). [file 1471-2164-12-S3-S18-S5.zip › Figure S4/Centroid/ACCAGCCU_rank-20_149363691.jpg]

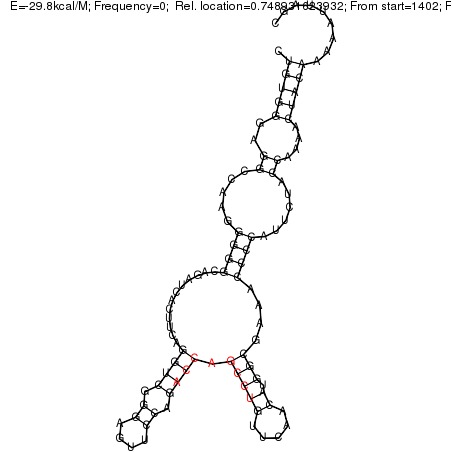

Supplement: Additional file 5 — Figure S4. Secondary structures for sequence region 0.7 to 1.0 of full length for the selected 32 eRNAs (see Fig. 7). [file 1471-2164-12-S3-S18-S5.zip › Figure S4/Centroid/ACCAGCCU_rank-21_7020962.jpg]

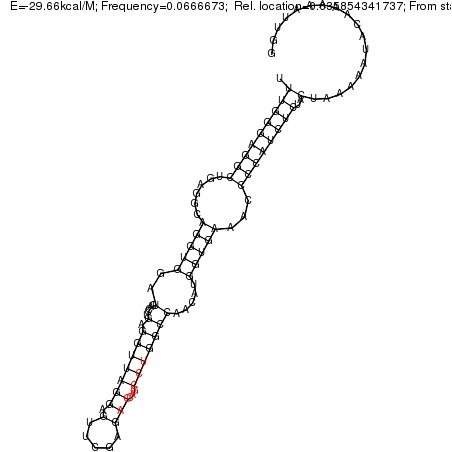

Supplement: Additional file 5 — Figure S4. Secondary structures for sequence region 0.7 to 1.0 of full length for the selected 32 eRNAs (see Fig. 7). [file 1471-2164-12-S3-S18-S5.zip › Figure S4/Centroid/ACCAGCCU_rank-22_149363691.jpg]

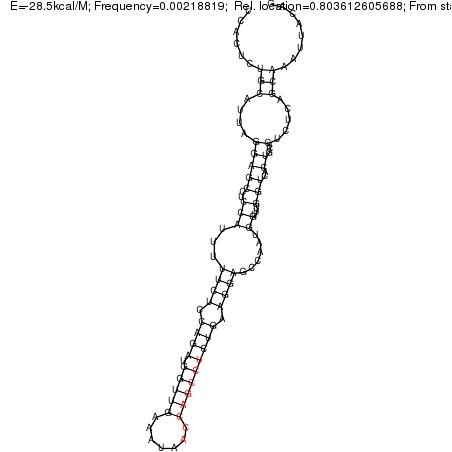

Supplement: Additional file 5 — Figure S4. Secondary structures for sequence region 0.7 to 1.0 of full length for the selected 32 eRNAs (see Fig. 7). [file 1471-2164-12-S3-S18-S5.zip › Figure S4/Centroid/ACCAGCCU_rank-23_16550670.jpg]

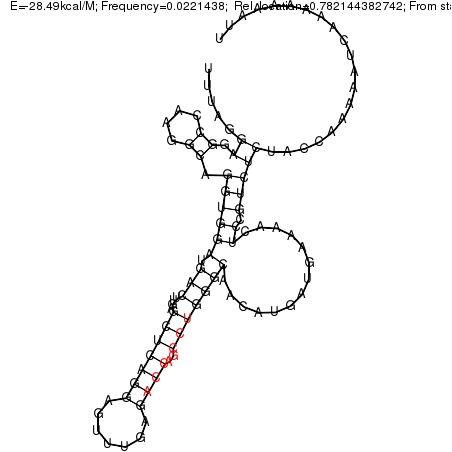

Supplement: Additional file 5 — Figure S4. Secondary structures for sequence region 0.7 to 1.0 of full length for the selected 32 eRNAs (see Fig. 7). [file 1471-2164-12-S3-S18-S5.zip › Figure S4/Centroid/ACCAGCCU_rank-24_7023439.jpg]

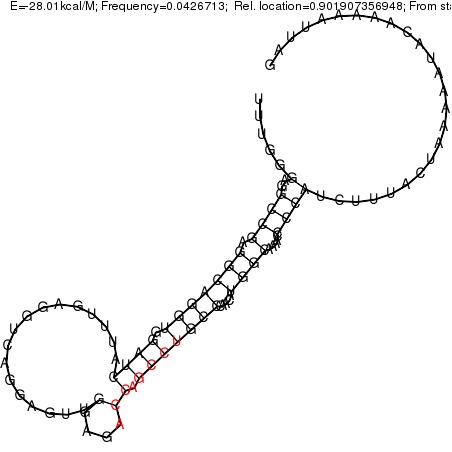

Supplement: Additional file 5 — Figure S4. Secondary structures for sequence region 0.7 to 1.0 of full length for the selected 32 eRNAs (see Fig. 7). [file 1471-2164-12-S3-S18-S5.zip › Figure S4/Centroid/ACCAGCCU_rank-25_37547428.jpg]

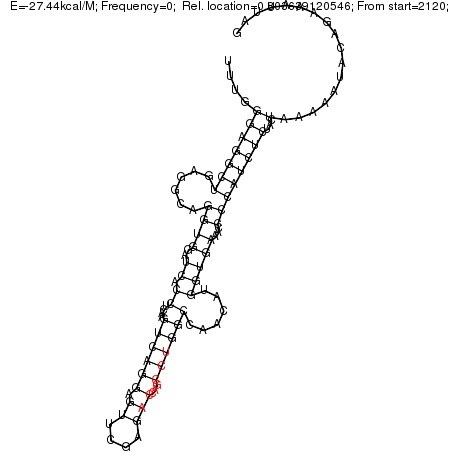

Supplement: Additional file 5 — Figure S4. Secondary structures for sequence region 0.7 to 1.0 of full length for the selected 32 eRNAs (see Fig. 7). [file 1471-2164-12-S3-S18-S5.zip › Figure S4/Centroid/ACCAGCCU_rank-26_85362714.jpg]

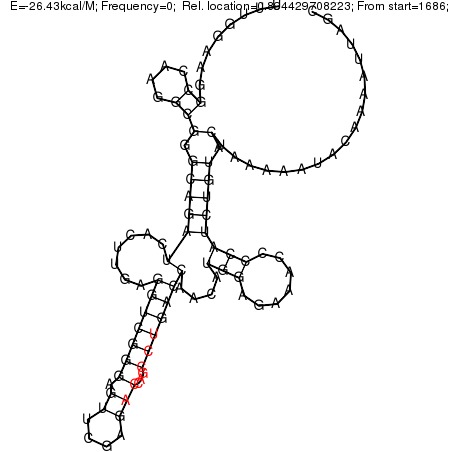

Supplement: Additional file 5 — Figure S4. Secondary structures for sequence region 0.7 to 1.0 of full length for the selected 32 eRNAs (see Fig. 7). [file 1471-2164-12-S3-S18-S5.zip › Figure S4/Centroid/ACCAGCCU_rank-27_14042003.jpg]

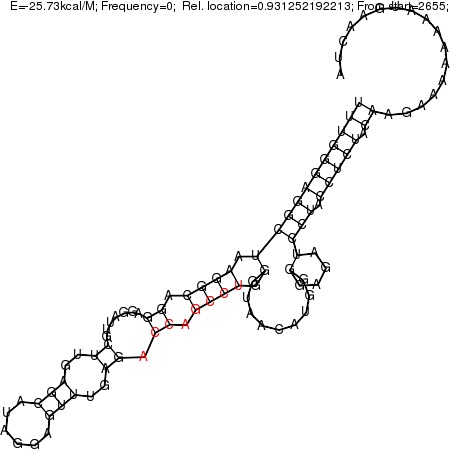

Supplement: Additional file 5 — Figure S4. Secondary structures for sequence region 0.7 to 1.0 of full length for the selected 32 eRNAs (see Fig. 7). [file 1471-2164-12-S3-S18-S5.zip › Figure S4/Centroid/ACCAGCCU_rank-28_18698598.jpg]

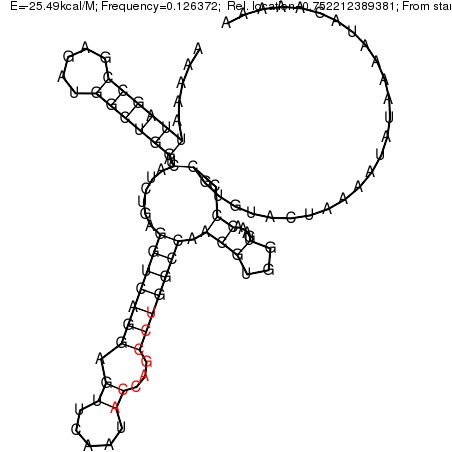

Supplement: Additional file 5 — Figure S4. Secondary structures for sequence region 0.7 to 1.0 of full length for the selected 32 eRNAs (see Fig. 7). [file 1471-2164-12-S3-S18-S5.zip › Figure S4/Centroid/ACCAGCCU_rank-29_10440286.jpg]

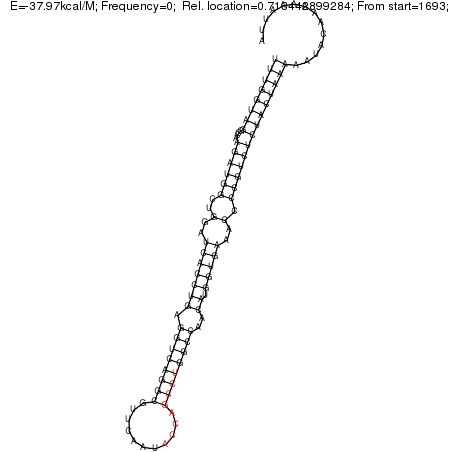

Supplement: Additional file 5 — Figure S4. Secondary structures for sequence region 0.7 to 1.0 of full length for the selected 32 eRNAs (see Fig. 7). [file 1471-2164-12-S3-S18-S5.zip › Figure S4/Centroid/ACCAGCCU_rank-2_10440286.jpg]

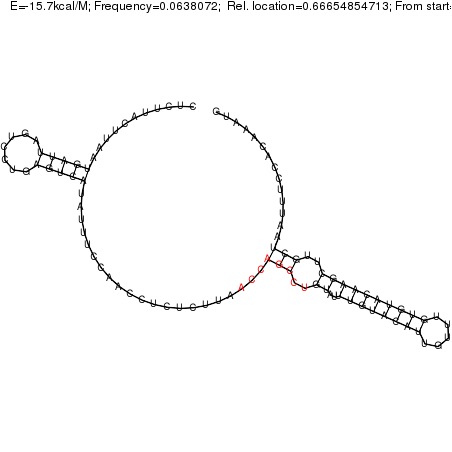

Supplement: Additional file 5 — Figure S4. Secondary structures for sequence region 0.7 to 1.0 of full length for the selected 32 eRNAs (see Fig. 7). [file 1471-2164-12-S3-S18-S5.zip › Figure S4/Centroid/ACCAGCCU_rank-30_21758081.jpg]

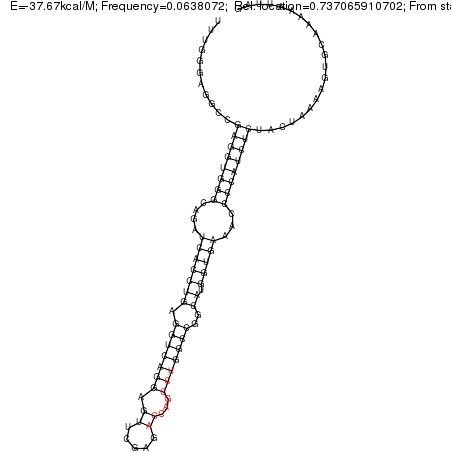

Supplement: Additional file 5 — Figure S4. Secondary structures for sequence region 0.7 to 1.0 of full length for the selected 32 eRNAs (see Fig. 7). [file 1471-2164-12-S3-S18-S5.zip › Figure S4/Centroid/ACCAGCCU_rank-3_21758081.jpg]

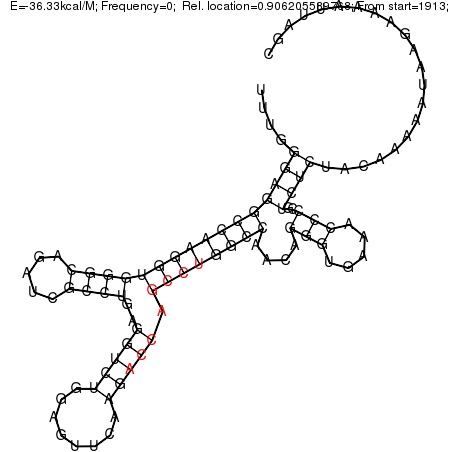

Supplement: Additional file 5 — Figure S4. Secondary structures for sequence region 0.7 to 1.0 of full length for the selected 32 eRNAs (see Fig. 7). [file 1471-2164-12-S3-S18-S5.zip › Figure S4/Centroid/ACCAGCCU_rank-4_34529621.jpg]

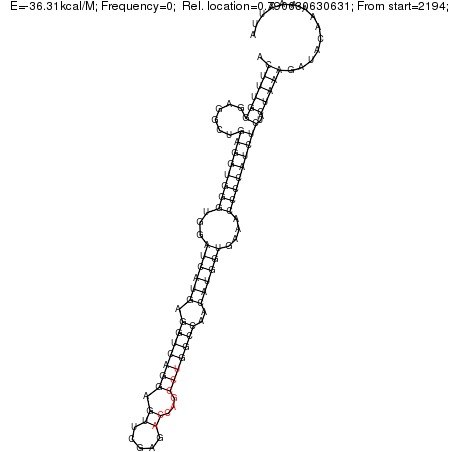

Supplement: Additional file 5 — Figure S4. Secondary structures for sequence region 0.7 to 1.0 of full length for the selected 32 eRNAs (see Fig. 7). [file 1471-2164-12-S3-S18-S5.zip › Figure S4/Centroid/ACCAGCCU_rank-5_21752136.jpg]

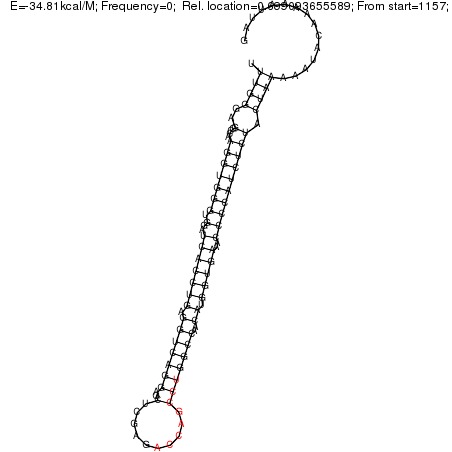

Supplement: Additional file 5 — Figure S4. Secondary structures for sequence region 0.7 to 1.0 of full length for the selected 32 eRNAs (see Fig. 7). [file 1471-2164-12-S3-S18-S5.zip › Figure S4/Centroid/ACCAGCCU_rank-6_32454755.jpg]

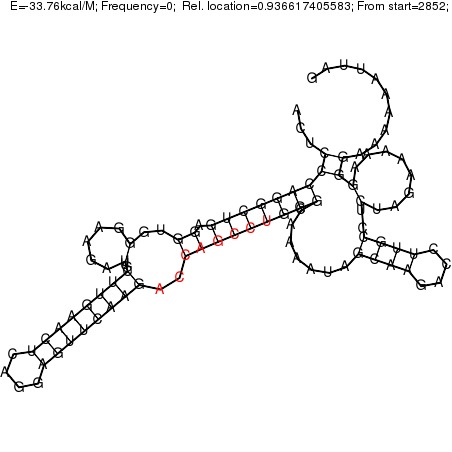

Supplement: Additional file 5 — Figure S4. Secondary structures for sequence region 0.7 to 1.0 of full length for the selected 32 eRNAs (see Fig. 7). [file 1471-2164-12-S3-S18-S5.zip › Figure S4/Centroid/ACCAGCCU_rank-7_61175255.jpg]

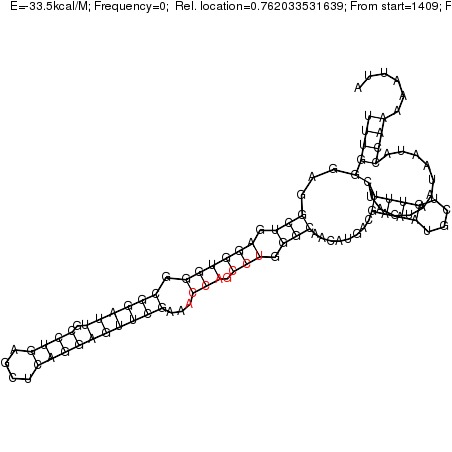

Supplement: Additional file 5 — Figure S4. Secondary structures for sequence region 0.7 to 1.0 of full length for the selected 32 eRNAs (see Fig. 7). [file 1471-2164-12-S3-S18-S5.zip › Figure S4/Centroid/ACCAGCCU_rank-8_10436389.jpg]

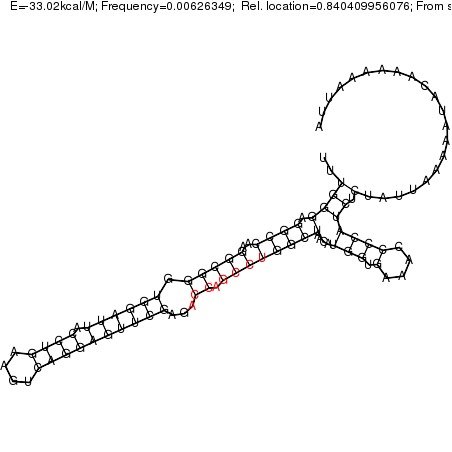

Supplement: Additional file 5 — Figure S4. Secondary structures for sequence region 0.7 to 1.0 of full length for the selected 32 eRNAs (see Fig. 7). [file 1471-2164-12-S3-S18-S5.zip › Figure S4/Centroid/ACCAGCCU_rank-9_10439148.jpg]

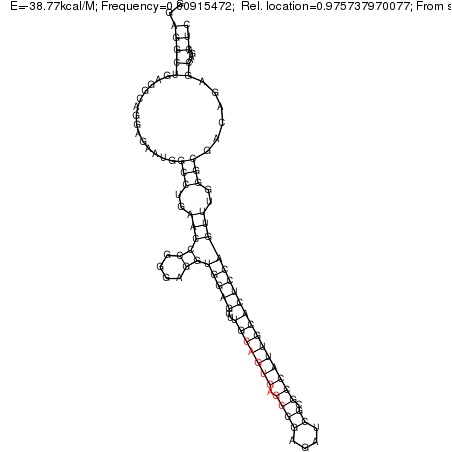

Supplement: Additional file 5 — Figure S4. Secondary structures for sequence region 0.7 to 1.0 of full length for the selected 32 eRNAs (see Fig. 7). [file 1471-2164-12-S3-S18-S5.zip › Figure S4/Centroid/CAGUGAGC_rank-10_10432841.jpg]

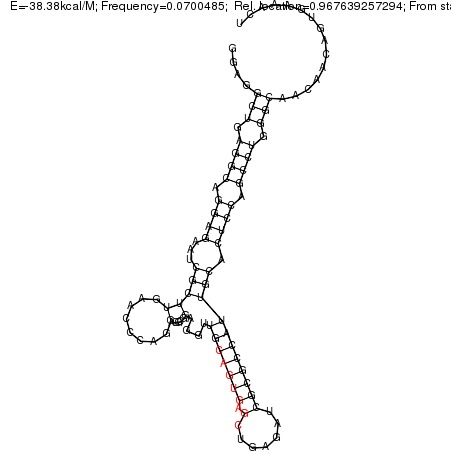

Supplement: Additional file 5 — Figure S4. Secondary structures for sequence region 0.7 to 1.0 of full length for the selected 32 eRNAs (see Fig. 7). [file 1471-2164-12-S3-S18-S5.zip › Figure S4/Centroid/CAGUGAGC_rank-11_14042003.jpg]

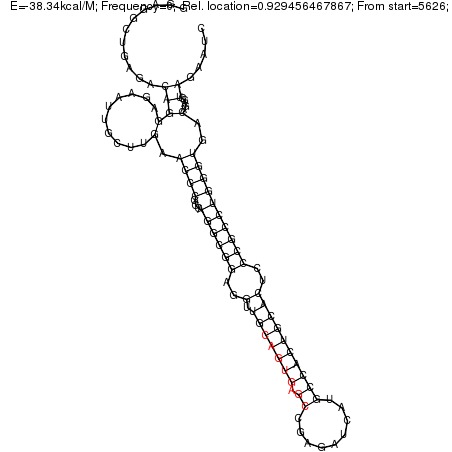

Supplement: Additional file 5 — Figure S4. Secondary structures for sequence region 0.7 to 1.0 of full length for the selected 32 eRNAs (see Fig. 7). [file 1471-2164-12-S3-S18-S5.zip › Figure S4/Centroid/CAGUGAGC_rank-12_10435879.jpg]

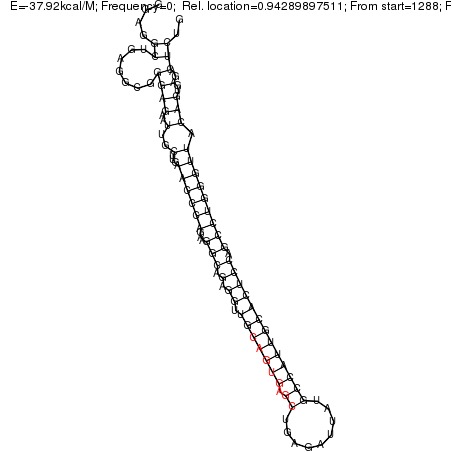

Supplement: Additional file 5 — Figure S4. Secondary structures for sequence region 0.7 to 1.0 of full length for the selected 32 eRNAs (see Fig. 7). [file 1471-2164-12-S3-S18-S5.zip › Figure S4/Centroid/CAGUGAGC_rank-13_10439148.jpg]

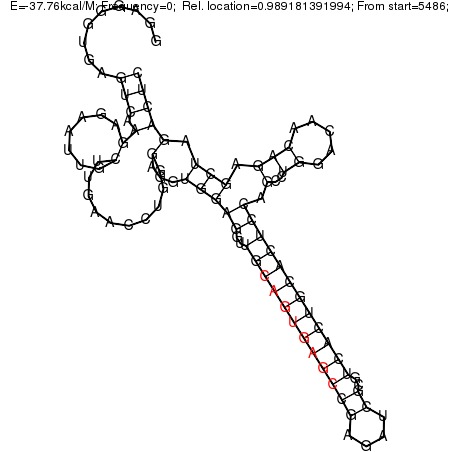

Supplement: Additional file 5 — Figure S4. Secondary structures for sequence region 0.7 to 1.0 of full length for the selected 32 eRNAs (see Fig. 7). [file 1471-2164-12-S3-S18-S5.zip › Figure S4/Centroid/CAGUGAGC_rank-14_10436764.jpg]

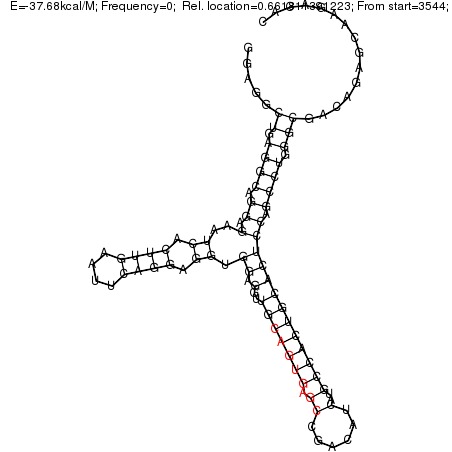

Supplement: Additional file 5 — Figure S4. Secondary structures for sequence region 0.7 to 1.0 of full length for the selected 32 eRNAs (see Fig. 7). [file 1471-2164-12-S3-S18-S5.zip › Figure S4/Centroid/CAGUGAGC_rank-15_149363691.jpg]

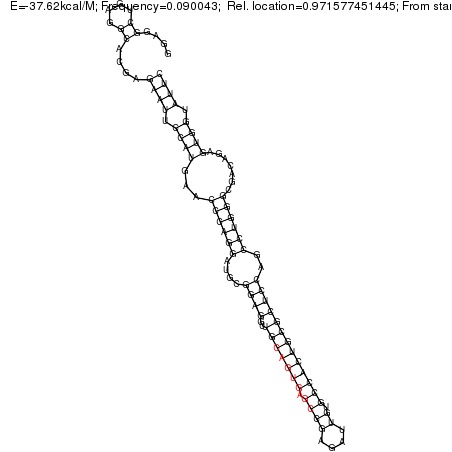

Supplement: Additional file 5 — Figure S4. Secondary structures for sequence region 0.7 to 1.0 of full length for the selected 32 eRNAs (see Fig. 7). [file 1471-2164-12-S3-S18-S5.zip › Figure S4/Centroid/CAGUGAGC_rank-16_34529621.jpg]

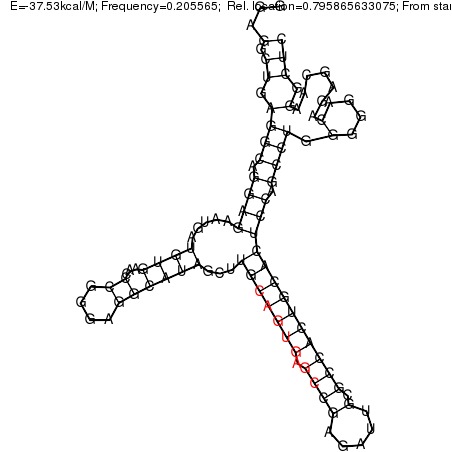

Supplement: Additional file 5 — Figure S4. Secondary structures for sequence region 0.7 to 1.0 of full length for the selected 32 eRNAs (see Fig. 7). [file 1471-2164-12-S3-S18-S5.zip › Figure S4/Centroid/CAGUGAGC_rank-17_6690226.jpg]

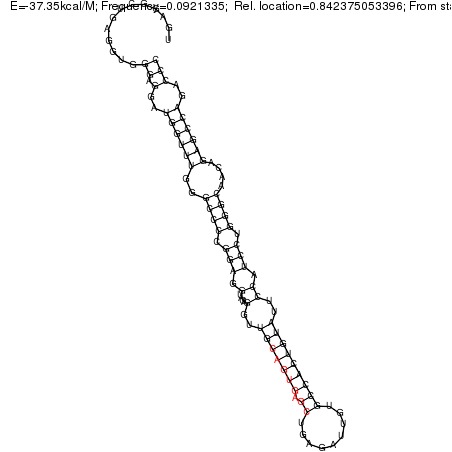

Supplement: Additional file 5 — Figure S4. Secondary structures for sequence region 0.7 to 1.0 of full length for the selected 32 eRNAs (see Fig. 7). [file 1471-2164-12-S3-S18-S5.zip › Figure S4/Centroid/CAGUGAGC_rank-18_7023439.jpg]

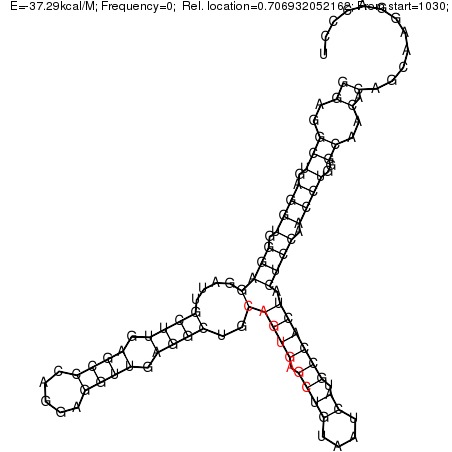

Supplement: Additional file 5 — Figure S4. Secondary structures for sequence region 0.7 to 1.0 of full length for the selected 32 eRNAs (see Fig. 7). [file 1471-2164-12-S3-S18-S5.zip › Figure S4/Centroid/CAGUGAGC_rank-19_32481159.jpg]

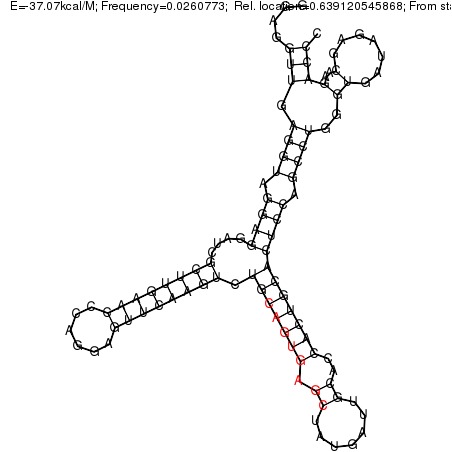

Supplement: Additional file 5 — Figure S4. Secondary structures for sequence region 0.7 to 1.0 of full length for the selected 32 eRNAs (see Fig. 7). [file 1471-2164-12-S3-S18-S5.zip › Figure S4/Centroid/CAGUGAGC_rank-20_85362714.jpg]

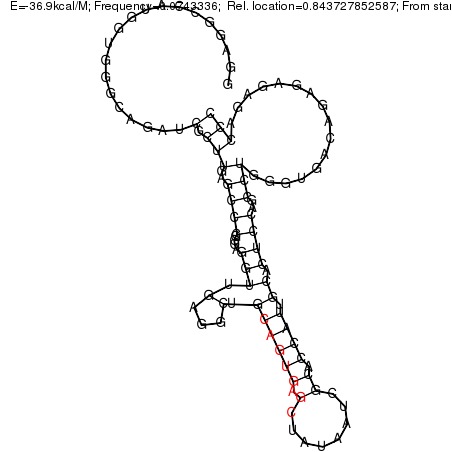

Supplement: Additional file 5 — Figure S4. Secondary structures for sequence region 0.7 to 1.0 of full length for the selected 32 eRNAs (see Fig. 7). [file 1471-2164-12-S3-S18-S5.zip › Figure S4/Centroid/CAGUGAGC_rank-21_21758081.jpg]

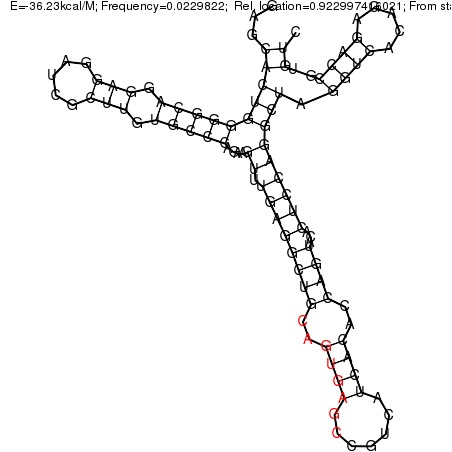

Supplement: Additional file 5 — Figure S4. Secondary structures for sequence region 0.7 to 1.0 of full length for the selected 32 eRNAs (see Fig. 7). [file 1471-2164-12-S3-S18-S5.zip › Figure S4/Centroid/CAGUGAGC_rank-22_6690226.jpg]

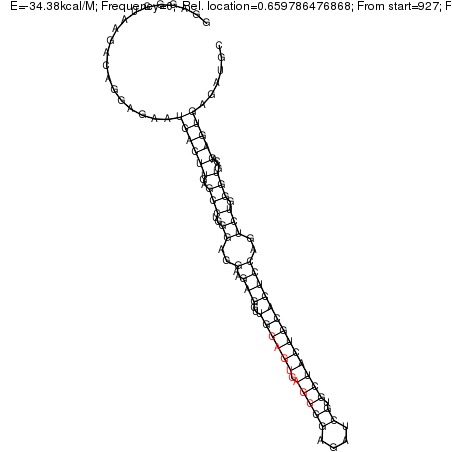

Supplement: Additional file 5 — Figure S4. Secondary structures for sequence region 0.7 to 1.0 of full length for the selected 32 eRNAs (see Fig. 7). [file 1471-2164-12-S3-S18-S5.zip › Figure S4/Centroid/CAGUGAGC_rank-25_10436915.jpg]

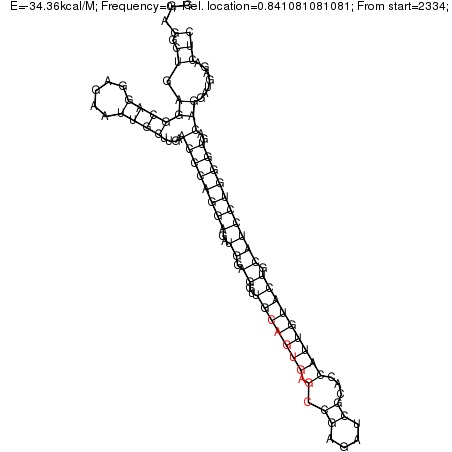

Supplement: Additional file 5 — Figure S4. Secondary structures for sequence region 0.7 to 1.0 of full length for the selected 32 eRNAs (see Fig. 7). [file 1471-2164-12-S3-S18-S5.zip › Figure S4/Centroid/CAGUGAGC_rank-26_21752136.jpg]

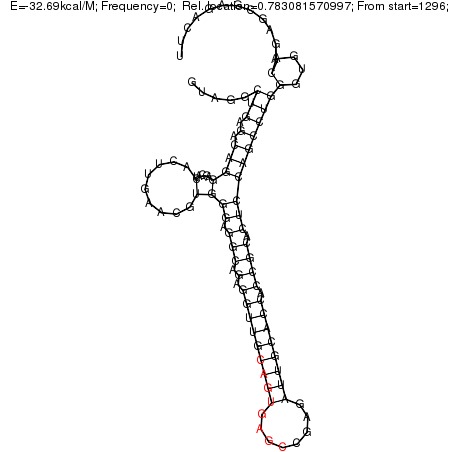

Supplement: Additional file 5 — Figure S4. Secondary structures for sequence region 0.7 to 1.0 of full length for the selected 32 eRNAs (see Fig. 7). [file 1471-2164-12-S3-S18-S5.zip › Figure S4/Centroid/CAGUGAGC_rank-27_32454755.jpg]

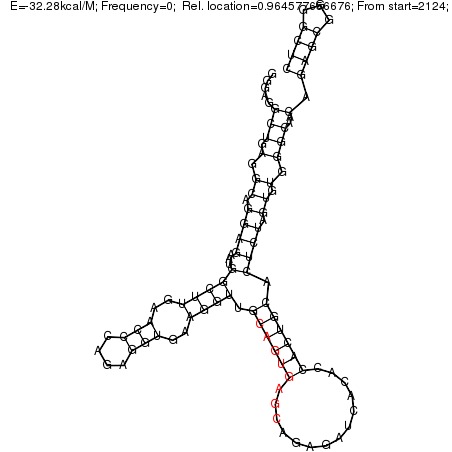

Supplement: Additional file 5 — Figure S4. Secondary structures for sequence region 0.7 to 1.0 of full length for the selected 32 eRNAs (see Fig. 7). [file 1471-2164-12-S3-S18-S5.zip › Figure S4/Centroid/CAGUGAGC_rank-28_37547428.jpg]

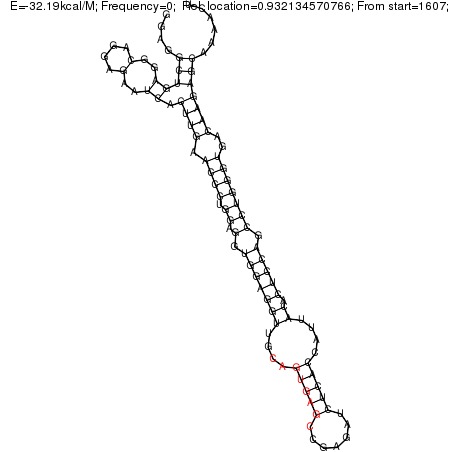

Supplement: Additional file 5 — Figure S4. Secondary structures for sequence region 0.7 to 1.0 of full length for the selected 32 eRNAs (see Fig. 7). [file 1471-2164-12-S3-S18-S5.zip › Figure S4/Centroid/CAGUGAGC_rank-29_45710101.jpg]

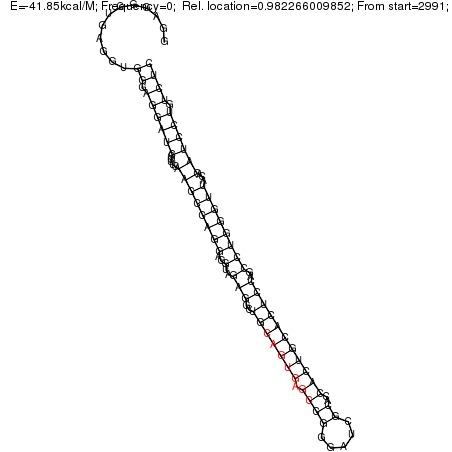

Supplement: Additional file 5 — Figure S4. Secondary structures for sequence region 0.7 to 1.0 of full length for the selected 32 eRNAs (see Fig. 7). [file 1471-2164-12-S3-S18-S5.zip › Figure S4/Centroid/CAGUGAGC_rank-2_61175255.jpg]

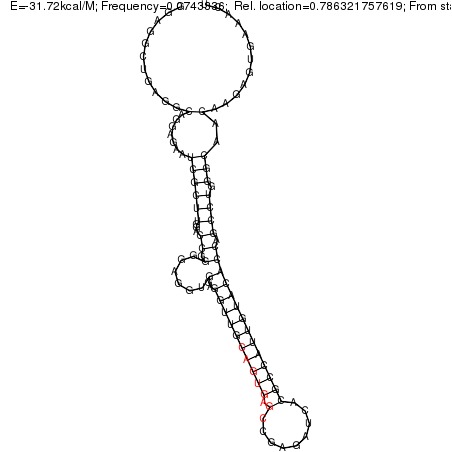

Supplement: Additional file 5 — Figure S4. Secondary structures for sequence region 0.7 to 1.0 of full length for the selected 32 eRNAs (see Fig. 7). [file 1471-2164-12-S3-S18-S5.zip › Figure S4/Centroid/CAGUGAGC_rank-30_21758081.jpg]

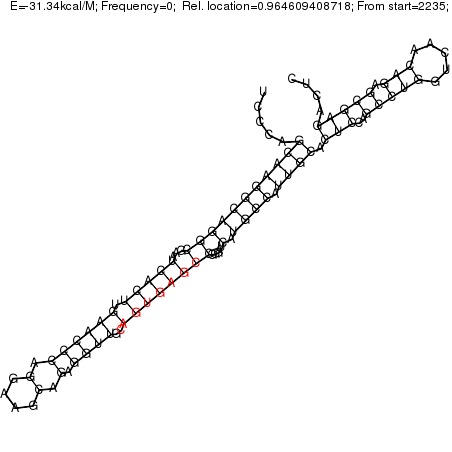

Supplement: Additional file 5 — Figure S4. Secondary structures for sequence region 0.7 to 1.0 of full length for the selected 32 eRNAs (see Fig. 7). [file 1471-2164-12-S3-S18-S5.zip › Figure S4/Centroid/CAGUGAGC_rank-31_30410976.jpg]

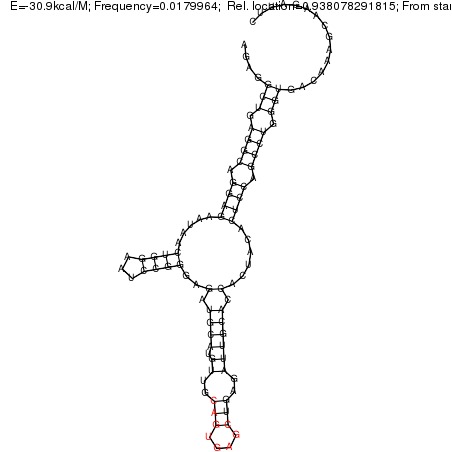

Supplement: Additional file 5 — Figure S4. Secondary structures for sequence region 0.7 to 1.0 of full length for the selected 32 eRNAs (see Fig. 7). [file 1471-2164-12-S3-S18-S5.zip › Figure S4/Centroid/CAGUGAGC_rank-32_10436915.jpg]

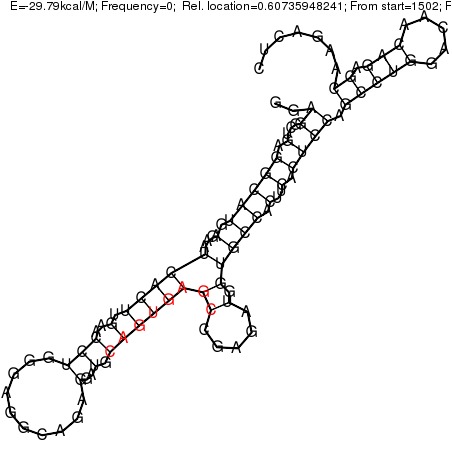

Supplement: Additional file 5 — Figure S4. Secondary structures for sequence region 0.7 to 1.0 of full length for the selected 32 eRNAs (see Fig. 7). [file 1471-2164-12-S3-S18-S5.zip › Figure S4/Centroid/CAGUGAGC_rank-33_10432841.jpg]

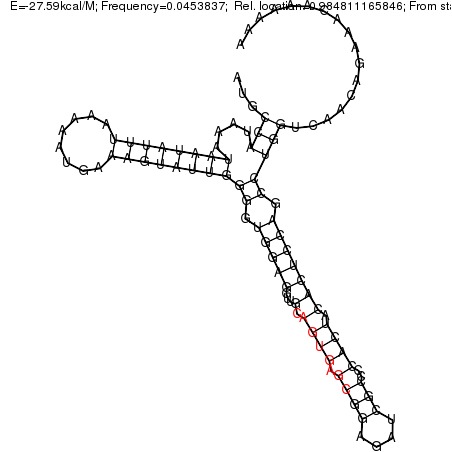

Supplement: Additional file 5 — Figure S4. Secondary structures for sequence region 0.7 to 1.0 of full length for the selected 32 eRNAs (see Fig. 7). [file 1471-2164-12-S3-S18-S5.zip › Figure S4/Centroid/CAGUGAGC_rank-35_71480116.jpg]

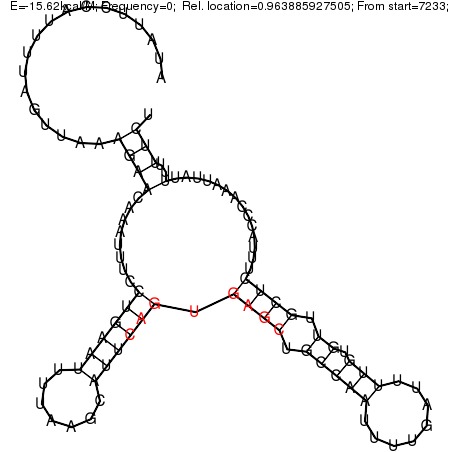

Supplement: Additional file 5 — Figure S4. Secondary structures for sequence region 0.7 to 1.0 of full length for the selected 32 eRNAs (see Fig. 7). [file 1471-2164-12-S3-S18-S5.zip › Figure S4/Centroid/CAGUGAGC_rank-36_156447020.jpg]

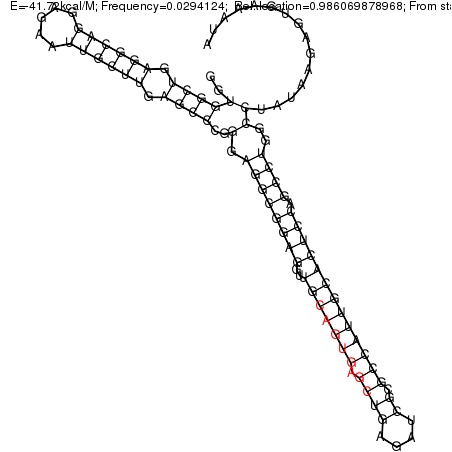

Supplement: Additional file 5 — Figure S4. Secondary structures for sequence region 0.7 to 1.0 of full length for the selected 32 eRNAs (see Fig. 7). [file 1471-2164-12-S3-S18-S5.zip › Figure S4/Centroid/CAGUGAGC_rank-3_12698046.jpg]

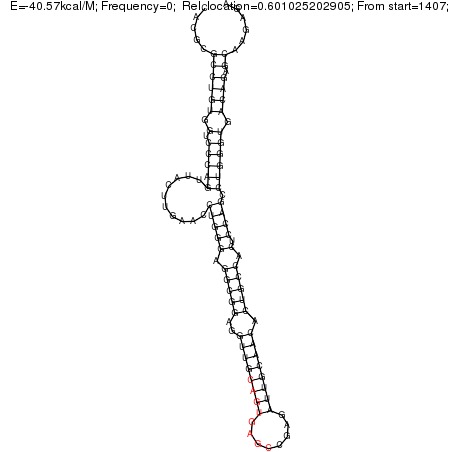

Supplement: Additional file 5 — Figure S4. Secondary structures for sequence region 0.7 to 1.0 of full length for the selected 32 eRNAs (see Fig. 7). [file 1471-2164-12-S3-S18-S5.zip › Figure S4/Centroid/CAGUGAGC_rank-4_7023439.jpg]

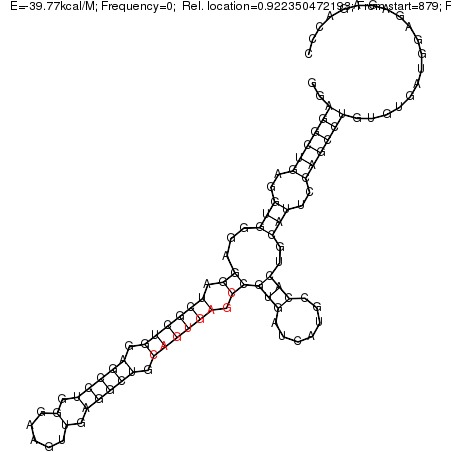

Supplement: Additional file 5 — Figure S4. Secondary structures for sequence region 0.7 to 1.0 of full length for the selected 32 eRNAs (see Fig. 7). [file 1471-2164-12-S3-S18-S5.zip › Figure S4/Centroid/CAGUGAGC_rank-5_4504692.jpg]

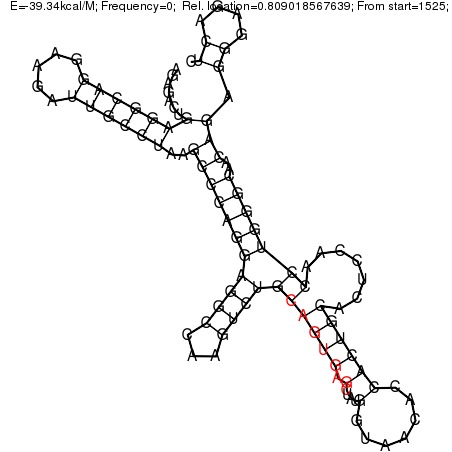

Supplement: Additional file 5 — Figure S4. Secondary structures for sequence region 0.7 to 1.0 of full length for the selected 32 eRNAs (see Fig. 7). [file 1471-2164-12-S3-S18-S5.zip › Figure S4/Centroid/CAGUGAGC_rank-7_14042003.jpg]

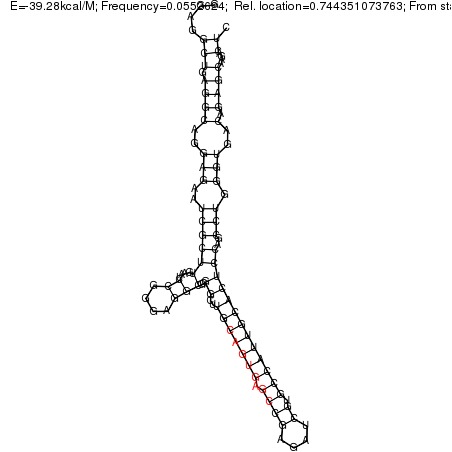

Supplement: Additional file 5 — Figure S4. Secondary structures for sequence region 0.7 to 1.0 of full length for the selected 32 eRNAs (see Fig. 7). [file 1471-2164-12-S3-S18-S5.zip › Figure S4/Centroid/CAGUGAGC_rank-8_149363691.jpg]

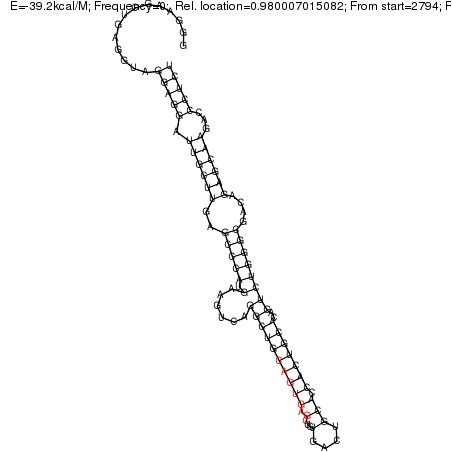

Supplement: Additional file 5 — Figure S4. Secondary structures for sequence region 0.7 to 1.0 of full length for the selected 32 eRNAs (see Fig. 7). [file 1471-2164-12-S3-S18-S5.zip › Figure S4/Centroid/CAGUGAGC_rank-9_18698598.jpg]

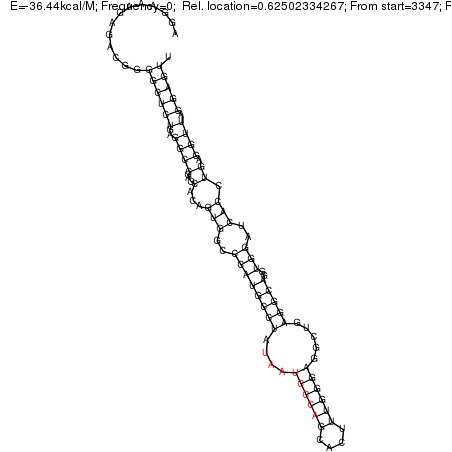

Supplement: Additional file 5 — Figure S4. Secondary structures for sequence region 0.7 to 1.0 of full length for the selected 32 eRNAs (see Fig. 7). [file 1471-2164-12-S3-S18-S5.zip › Figure S4/Centroid/UAAUCCCA_rank-10_149363691.jpg]

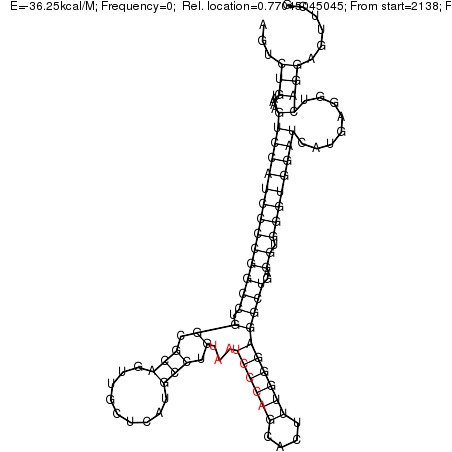

Supplement: Additional file 5 — Figure S4. Secondary structures for sequence region 0.7 to 1.0 of full length for the selected 32 eRNAs (see Fig. 7). [file 1471-2164-12-S3-S18-S5.zip › Figure S4/Centroid/UAAUCCCA_rank-11_21752136.jpg]

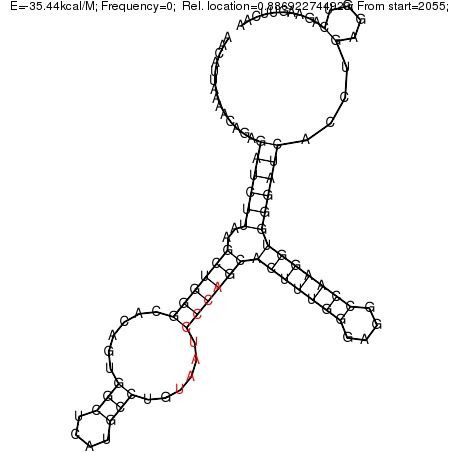

Supplement: Additional file 5 — Figure S4. Secondary structures for sequence region 0.7 to 1.0 of full length for the selected 32 eRNAs (see Fig. 7). [file 1471-2164-12-S3-S18-S5.zip › Figure S4/Centroid/UAAUCCCA_rank-13_30410976.jpg]

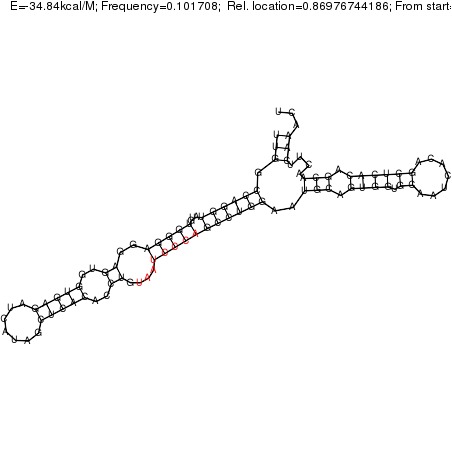

Supplement: Additional file 5 — Figure S4. Secondary structures for sequence region 0.7 to 1.0 of full length for the selected 32 eRNAs (see Fig. 7). [file 1471-2164-12-S3-S18-S5.zip › Figure S4/Centroid/UAAUCCCA_rank-14_6690226.jpg]

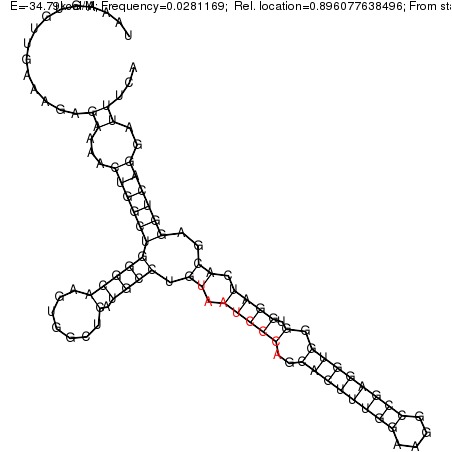

Supplement: Additional file 5 — Figure S4. Secondary structures for sequence region 0.7 to 1.0 of full length for the selected 32 eRNAs (see Fig. 7). [file 1471-2164-12-S3-S18-S5.zip › Figure S4/Centroid/UAAUCCCA_rank-15_10432841.jpg]

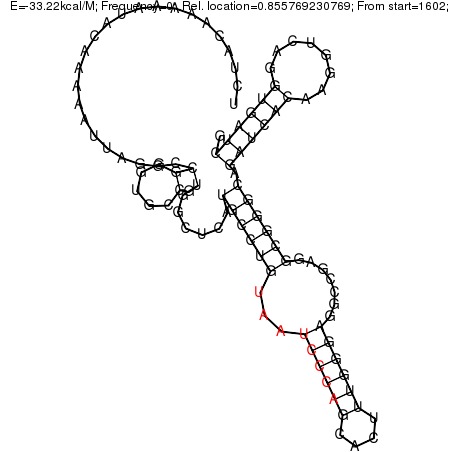

Supplement: Additional file 5 — Figure S4. Secondary structures for sequence region 0.7 to 1.0 of full length for the selected 32 eRNAs (see Fig. 7). [file 1471-2164-12-S3-S18-S5.zip › Figure S4/Centroid/UAAUCCCA_rank-19_7020962.jpg]

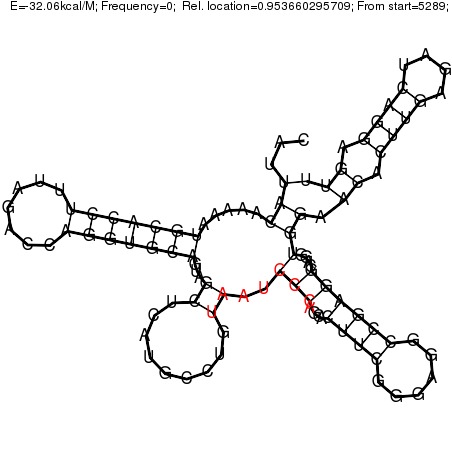

Supplement: Additional file 5 — Figure S4. Secondary structures for sequence region 0.7 to 1.0 of full length for the selected 32 eRNAs (see Fig. 7). [file 1471-2164-12-S3-S18-S5.zip › Figure S4/Centroid/UAAUCCCA_rank-21_10436764.jpg]

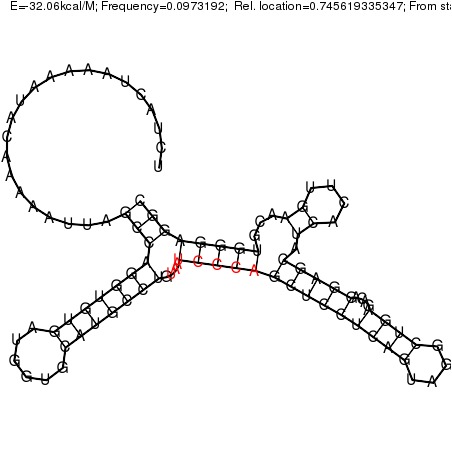

Supplement: Additional file 5 — Figure S4. Secondary structures for sequence region 0.7 to 1.0 of full length for the selected 32 eRNAs (see Fig. 7). [file 1471-2164-12-S3-S18-S5.zip › Figure S4/Centroid/UAAUCCCA_rank-22_32454755.jpg]

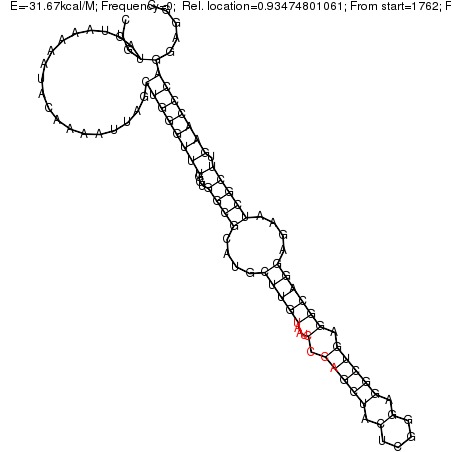

Supplement: Additional file 5 — Figure S4. Secondary structures for sequence region 0.7 to 1.0 of full length for the selected 32 eRNAs (see Fig. 7). [file 1471-2164-12-S3-S18-S5.zip › Figure S4/Centroid/UAAUCCCA_rank-23_14042003.jpg]

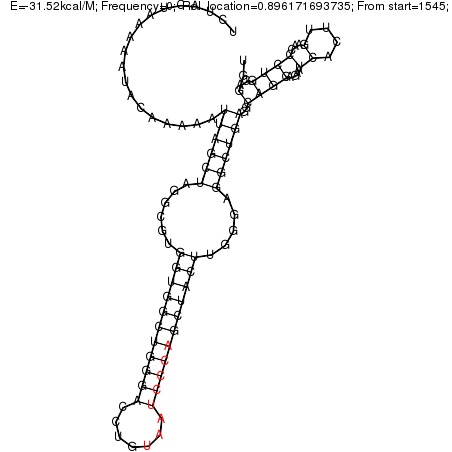

Supplement: Additional file 5 — Figure S4. Secondary structures for sequence region 0.7 to 1.0 of full length for the selected 32 eRNAs (see Fig. 7). [file 1471-2164-12-S3-S18-S5.zip › Figure S4/Centroid/UAAUCCCA_rank-24_45710101.jpg]

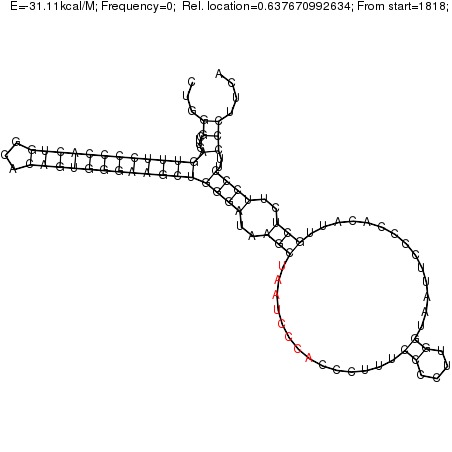

Supplement: Additional file 5 — Figure S4. Secondary structures for sequence region 0.7 to 1.0 of full length for the selected 32 eRNAs (see Fig. 7). [file 1471-2164-12-S3-S18-S5.zip › Figure S4/Centroid/UAAUCCCA_rank-26_18698598.jpg]

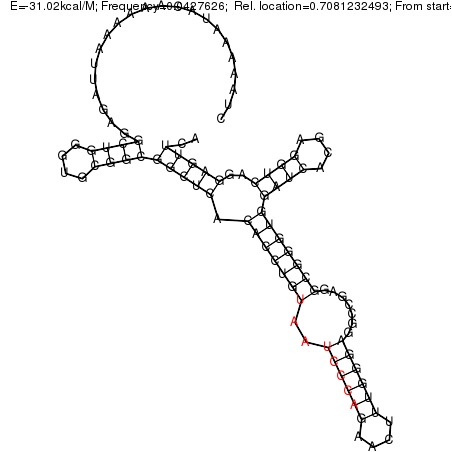

Supplement: Additional file 5 — Figure S4. Secondary structures for sequence region 0.7 to 1.0 of full length for the selected 32 eRNAs (see Fig. 7). [file 1471-2164-12-S3-S18-S5.zip › Figure S4/Centroid/UAAUCCCA_rank-27_149363691.jpg]

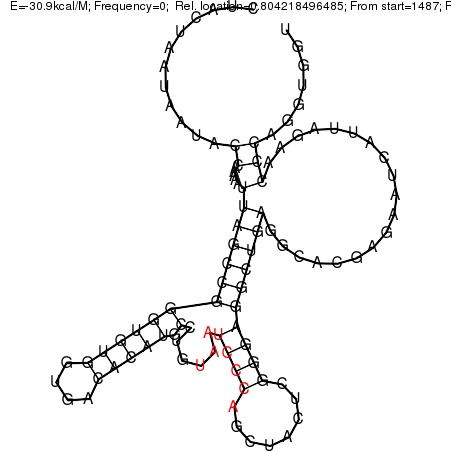

Supplement: Additional file 5 — Figure S4. Secondary structures for sequence region 0.7 to 1.0 of full length for the selected 32 eRNAs (see Fig. 7). [file 1471-2164-12-S3-S18-S5.zip › Figure S4/Centroid/UAAUCCCA_rank-28_10436389.jpg]

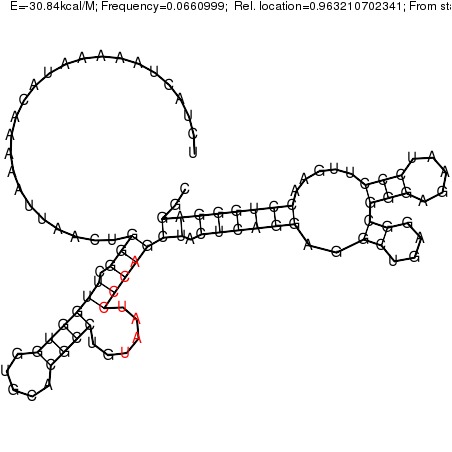

Supplement: Additional file 5 — Figure S4. Secondary structures for sequence region 0.7 to 1.0 of full length for the selected 32 eRNAs (see Fig. 7). [file 1471-2164-12-S3-S18-S5.zip › Figure S4/Centroid/UAAUCCCA_rank-29_92859582.jpg]

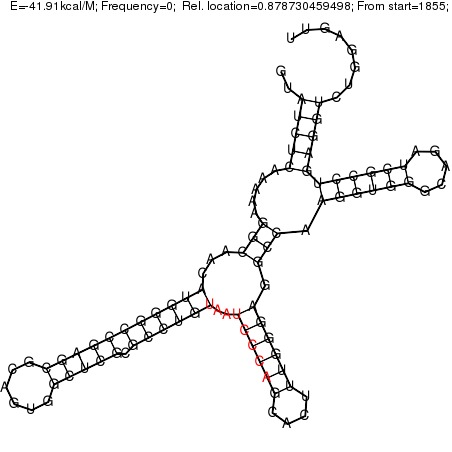

Supplement: Additional file 5 — Figure S4. Secondary structures for sequence region 0.7 to 1.0 of full length for the selected 32 eRNAs (see Fig. 7). [file 1471-2164-12-S3-S18-S5.zip › Figure S4/Centroid/UAAUCCCA_rank-2_34529621.jpg]

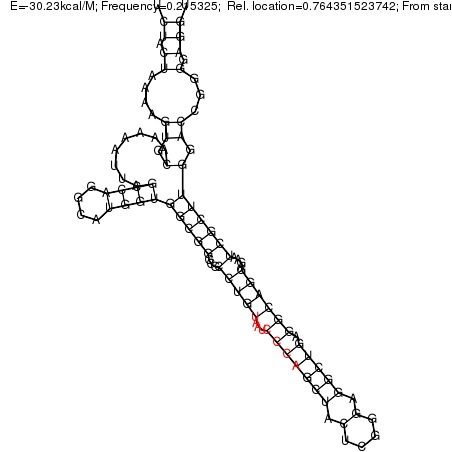

Supplement: Additional file 5 — Figure S4. Secondary structures for sequence region 0.7 to 1.0 of full length for the selected 32 eRNAs (see Fig. 7). [file 1471-2164-12-S3-S18-S5.zip › Figure S4/Centroid/UAAUCCCA_rank-30_21758081.jpg]

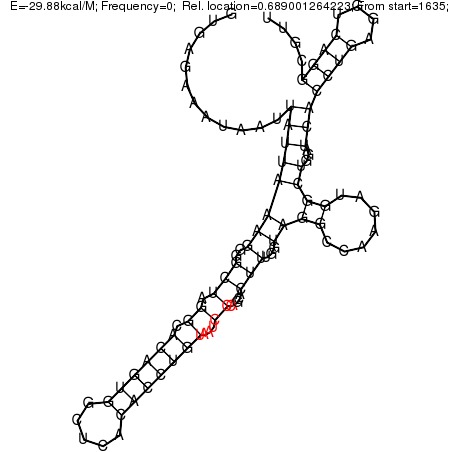

Supplement: Additional file 5 — Figure S4. Secondary structures for sequence region 0.7 to 1.0 of full length for the selected 32 eRNAs (see Fig. 7). [file 1471-2164-12-S3-S18-S5.zip › Figure S4/Centroid/UAAUCCCA_rank-31_10440286.jpg]

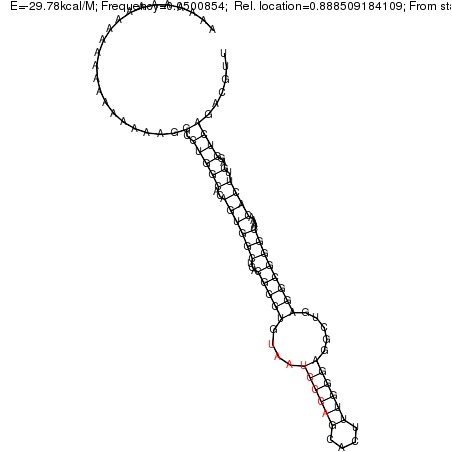

Supplement: Additional file 5 — Figure S4. Secondary structures for sequence region 0.7 to 1.0 of full length for the selected 32 eRNAs (see Fig. 7). [file 1471-2164-12-S3-S18-S5.zip › Figure S4/Centroid/UAAUCCCA_rank-32_7023439.jpg]

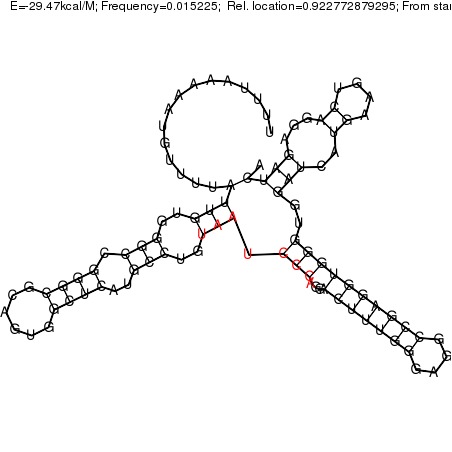

Supplement: Additional file 5 — Figure S4. Secondary structures for sequence region 0.7 to 1.0 of full length for the selected 32 eRNAs (see Fig. 7). [file 1471-2164-12-S3-S18-S5.zip › Figure S4/Centroid/UAAUCCCA_rank-33_92859582.jpg]

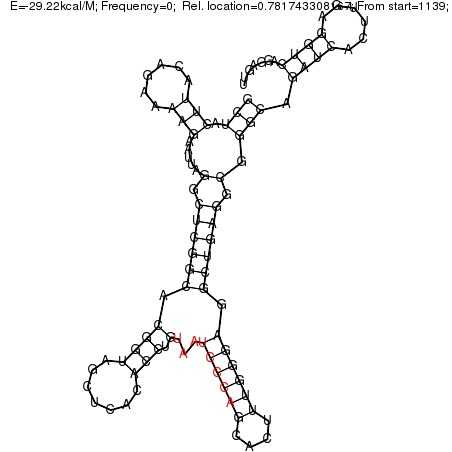

Supplement: Additional file 5 — Figure S4. Secondary structures for sequence region 0.7 to 1.0 of full length for the selected 32 eRNAs (see Fig. 7). [file 1471-2164-12-S3-S18-S5.zip › Figure S4/Centroid/UAAUCCCA_rank-34_32481159.jpg]

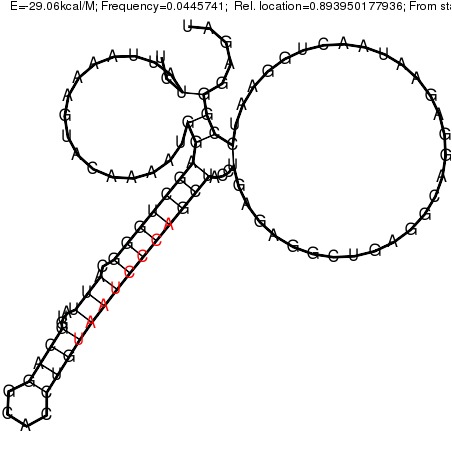

Supplement: Additional file 5 — Figure S4. Secondary structures for sequence region 0.7 to 1.0 of full length for the selected 32 eRNAs (see Fig. 7). [file 1471-2164-12-S3-S18-S5.zip › Figure S4/Centroid/UAAUCCCA_rank-35_10436915.jpg]

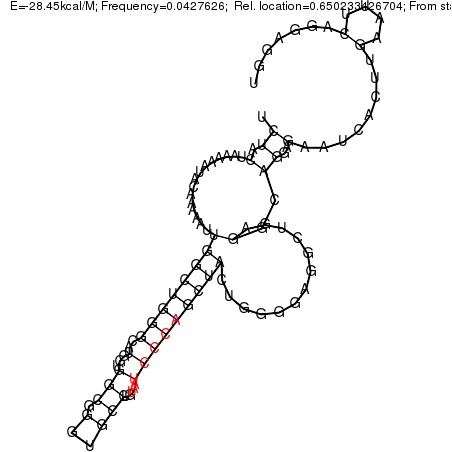

Supplement: Additional file 5 — Figure S4. Secondary structures for sequence region 0.7 to 1.0 of full length for the selected 32 eRNAs (see Fig. 7). [file 1471-2164-12-S3-S18-S5.zip › Figure S4/Centroid/UAAUCCCA_rank-36_149363691.jpg]

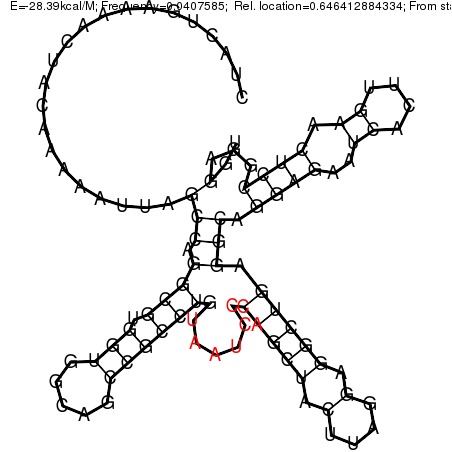

Supplement: Additional file 5 — Figure S4. Secondary structures for sequence region 0.7 to 1.0 of full length for the selected 32 eRNAs (see Fig. 7). [file 1471-2164-12-S3-S18-S5.zip › Figure S4/Centroid/UAAUCCCA_rank-37_10439148.jpg]

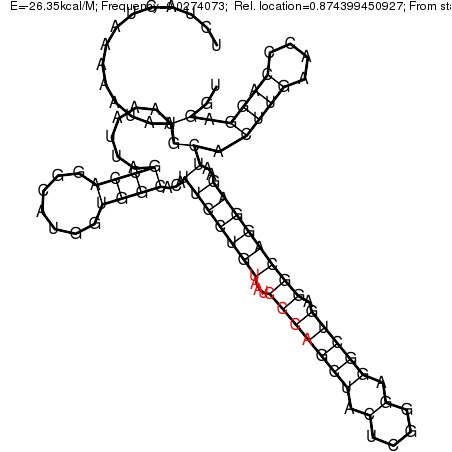

Supplement: Additional file 5 — Figure S4. Secondary structures for sequence region 0.7 to 1.0 of full length for the selected 32 eRNAs (see Fig. 7). [file 1471-2164-12-S3-S18-S5.zip › Figure S4/Centroid/UAAUCCCA_rank-39_32481159.jpg]

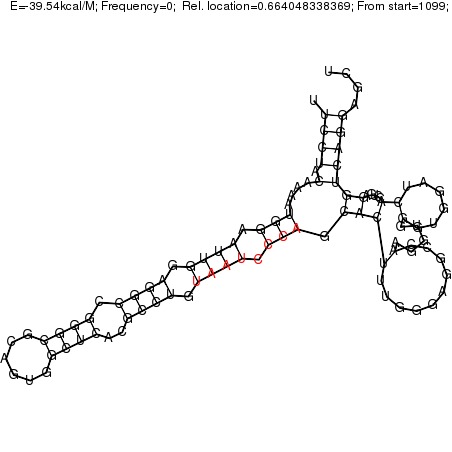

Supplement: Additional file 5 — Figure S4. Secondary structures for sequence region 0.7 to 1.0 of full length for the selected 32 eRNAs (see Fig. 7). [file 1471-2164-12-S3-S18-S5.zip › Figure S4/Centroid/UAAUCCCA_rank-3_32454755.jpg]

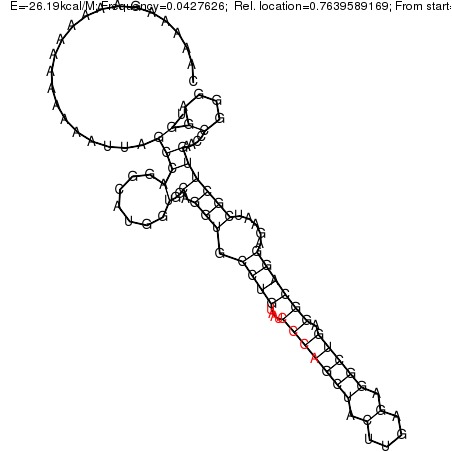

Supplement: Additional file 5 — Figure S4. Secondary structures for sequence region 0.7 to 1.0 of full length for the selected 32 eRNAs (see Fig. 7). [file 1471-2164-12-S3-S18-S5.zip › Figure S4/Centroid/UAAUCCCA_rank-40_149363691.jpg]

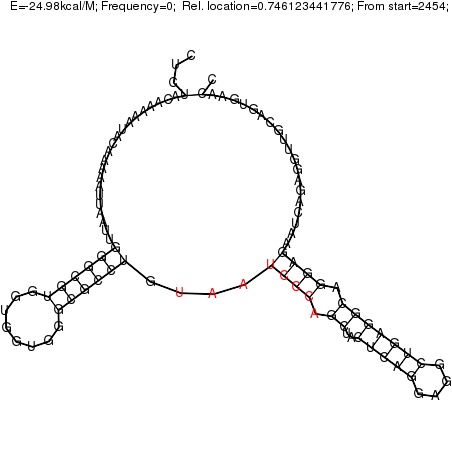

Supplement: Additional file 5 — Figure S4. Secondary structures for sequence region 0.7 to 1.0 of full length for the selected 32 eRNAs (see Fig. 7). [file 1471-2164-12-S3-S18-S5.zip › Figure S4/Centroid/UAAUCCCA_rank-41_92859582.jpg]

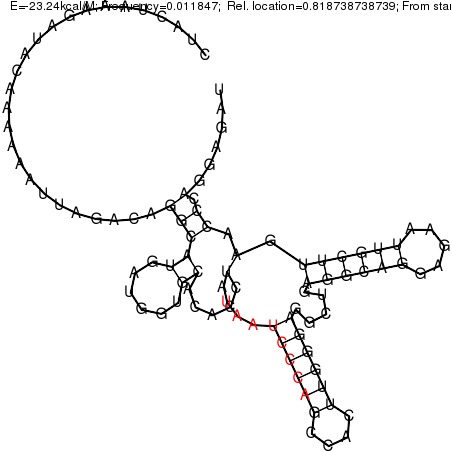

Supplement: Additional file 5 — Figure S4. Secondary structures for sequence region 0.7 to 1.0 of full length for the selected 32 eRNAs (see Fig. 7). [file 1471-2164-12-S3-S18-S5.zip › Figure S4/Centroid/UAAUCCCA_rank-42_21752136.jpg]

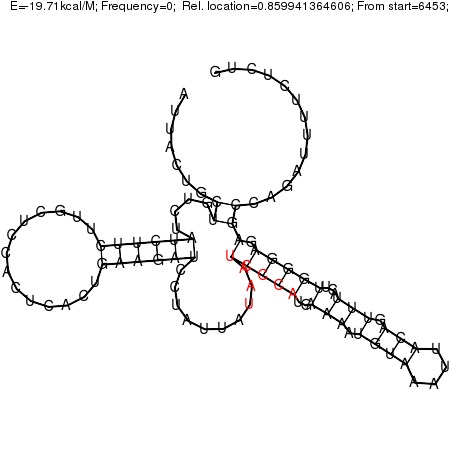

Supplement: Additional file 5 — Figure S4. Secondary structures for sequence region 0.7 to 1.0 of full length for the selected 32 eRNAs (see Fig. 7). [file 1471-2164-12-S3-S18-S5.zip › Figure S4/Centroid/UAAUCCCA_rank-43_156447020.jpg]

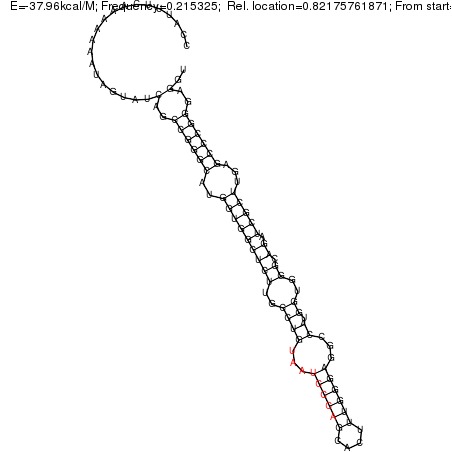

Supplement: Additional file 5 — Figure S4. Secondary structures for sequence region 0.7 to 1.0 of full length for the selected 32 eRNAs (see Fig. 7). [file 1471-2164-12-S3-S18-S5.zip › Figure S4/Centroid/UAAUCCCA_rank-5_21758081.jpg]

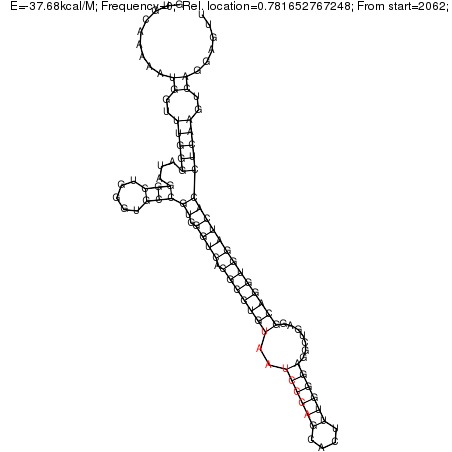

Supplement: Additional file 5 — Figure S4. Secondary structures for sequence region 0.7 to 1.0 of full length for the selected 32 eRNAs (see Fig. 7). [file 1471-2164-12-S3-S18-S5.zip › Figure S4/Centroid/UAAUCCCA_rank-6_85362714.jpg]

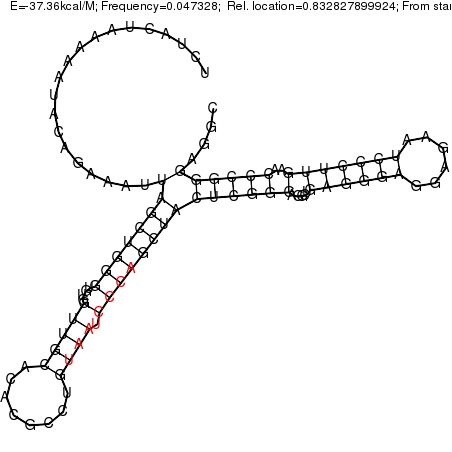

Supplement: Additional file 5 — Figure S4. Secondary structures for sequence region 0.7 to 1.0 of full length for the selected 32 eRNAs (see Fig. 7). [file 1471-2164-12-S3-S18-S5.zip › Figure S4/Centroid/UAAUCCCA_rank-7_85362714.jpg]

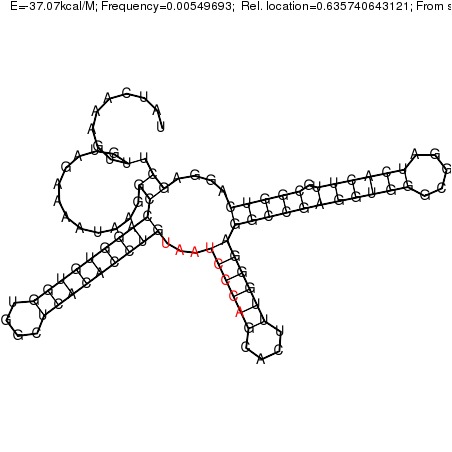

Supplement: Additional file 5 — Figure S4. Secondary structures for sequence region 0.7 to 1.0 of full length for the selected 32 eRNAs (see Fig. 7). [file 1471-2164-12-S3-S18-S5.zip › Figure S4/Centroid/UAAUCCCA_rank-8_153252197.jpg]

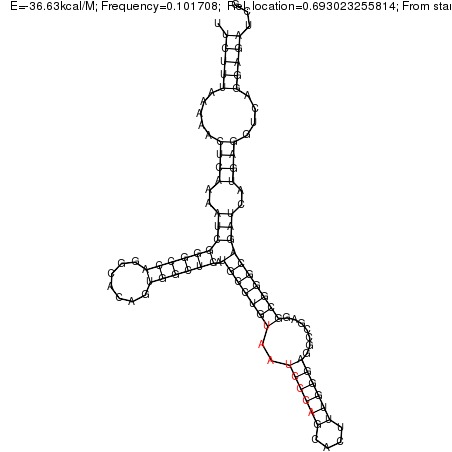

Supplement: Additional file 5 — Figure S4. Secondary structures for sequence region 0.7 to 1.0 of full length for the selected 32 eRNAs (see Fig. 7). [file 1471-2164-12-S3-S18-S5.zip › Figure S4/Centroid/UAAUCCCA_rank-9_6690226.jpg]

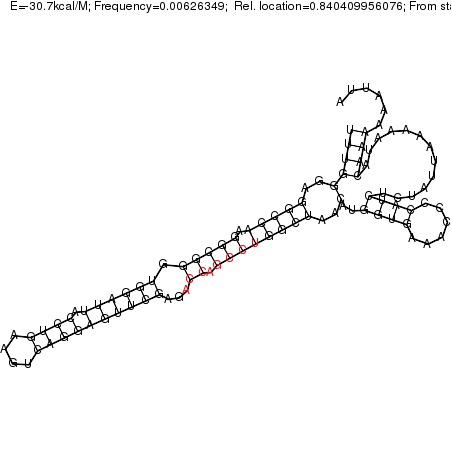

Supplement: Additional file 5 — Figure S4. Secondary structures for sequence region 0.7 to 1.0 of full length for the selected 32 eRNAs (see Fig. 7). [file 1471-2164-12-S3-S18-S5.zip › Figure S4/MFE/ACCAGCCU_rank-10_10439148.jpg]

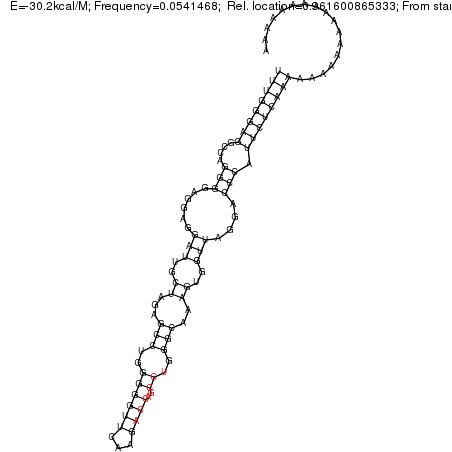

Supplement: Additional file 5 — Figure S4. Secondary structures for sequence region 0.7 to 1.0 of full length for the selected 32 eRNAs (see Fig. 7). [file 1471-2164-12-S3-S18-S5.zip › Figure S4/MFE/ACCAGCCU_rank-11_10436389.jpg]

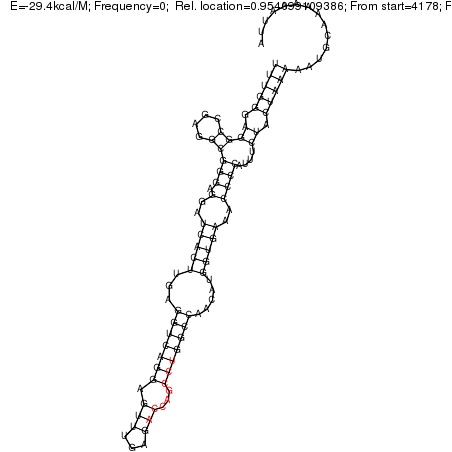

Supplement: Additional file 5 — Figure S4. Secondary structures for sequence region 0.7 to 1.0 of full length for the selected 32 eRNAs (see Fig. 7). [file 1471-2164-12-S3-S18-S5.zip › Figure S4/MFE/ACCAGCCU_rank-12_12698046.jpg]

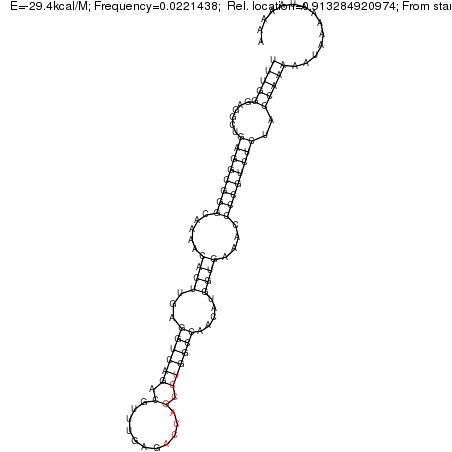

Supplement: Additional file 5 — Figure S4. Secondary structures for sequence region 0.7 to 1.0 of full length for the selected 32 eRNAs (see Fig. 7). [file 1471-2164-12-S3-S18-S5.zip › Figure S4/MFE/ACCAGCCU_rank-13_7023439.jpg]

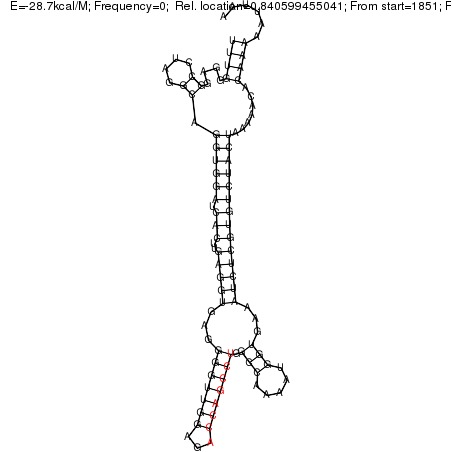

Supplement: Additional file 5 — Figure S4. Secondary structures for sequence region 0.7 to 1.0 of full length for the selected 32 eRNAs (see Fig. 7). [file 1471-2164-12-S3-S18-S5.zip › Figure S4/MFE/ACCAGCCU_rank-14_37547428.jpg]

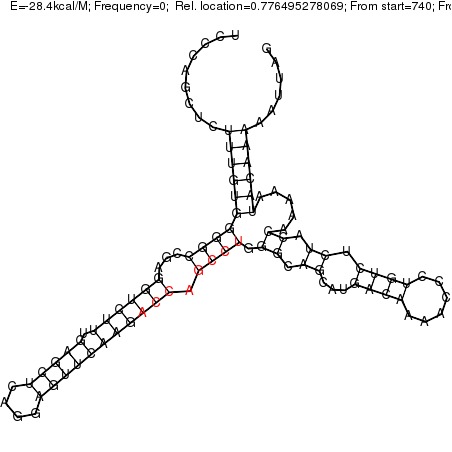

Supplement: Additional file 5 — Figure S4. Secondary structures for sequence region 0.7 to 1.0 of full length for the selected 32 eRNAs (see Fig. 7). [file 1471-2164-12-S3-S18-S5.zip › Figure S4/MFE/ACCAGCCU_rank-15_4504692.jpg]

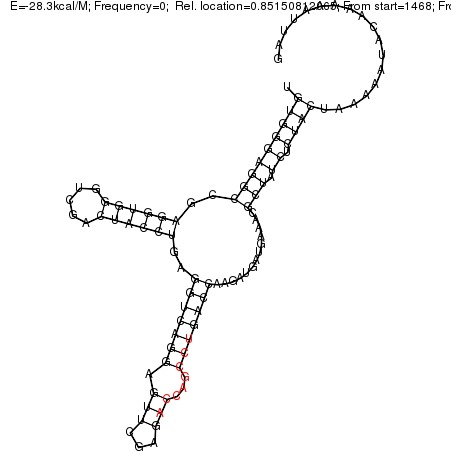

Supplement: Additional file 5 — Figure S4. Secondary structures for sequence region 0.7 to 1.0 of full length for the selected 32 eRNAs (see Fig. 7). [file 1471-2164-12-S3-S18-S5.zip › Figure S4/MFE/ACCAGCCU_rank-16_45710101.jpg]

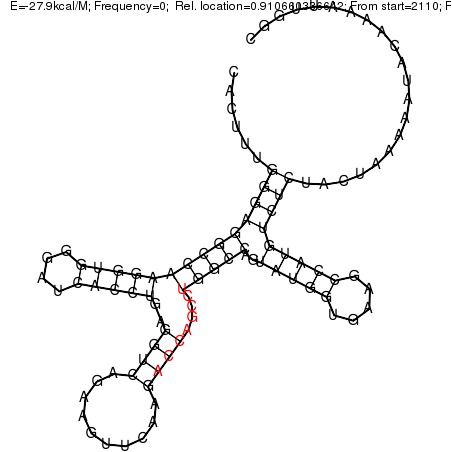

Supplement: Additional file 5 — Figure S4. Secondary structures for sequence region 0.7 to 1.0 of full length for the selected 32 eRNAs (see Fig. 7). [file 1471-2164-12-S3-S18-S5.zip › Figure S4/MFE/ACCAGCCU_rank-17_30410976.jpg]

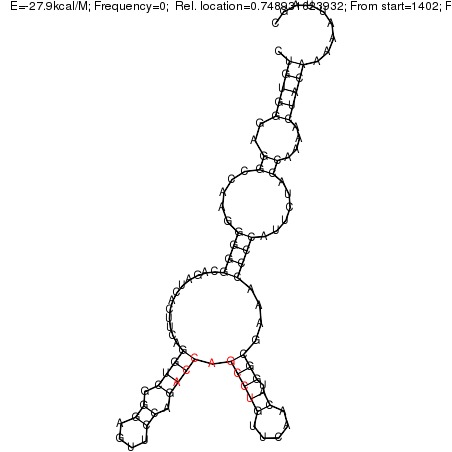

Supplement: Additional file 5 — Figure S4. Secondary structures for sequence region 0.7 to 1.0 of full length for the selected 32 eRNAs (see Fig. 7). [file 1471-2164-12-S3-S18-S5.zip › Figure S4/MFE/ACCAGCCU_rank-18_7020962.jpg]

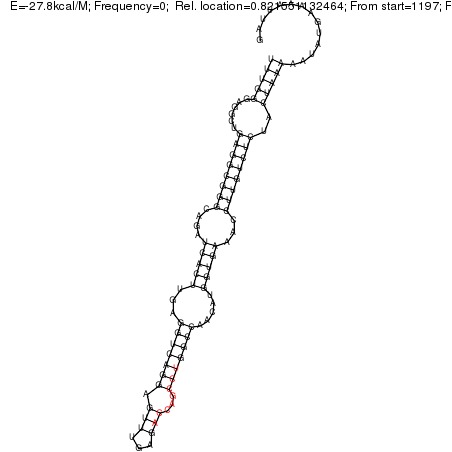

Supplement: Additional file 5 — Figure S4. Secondary structures for sequence region 0.7 to 1.0 of full length for the selected 32 eRNAs (see Fig. 7). [file 1471-2164-12-S3-S18-S5.zip › Figure S4/MFE/ACCAGCCU_rank-19_32481159.jpg]

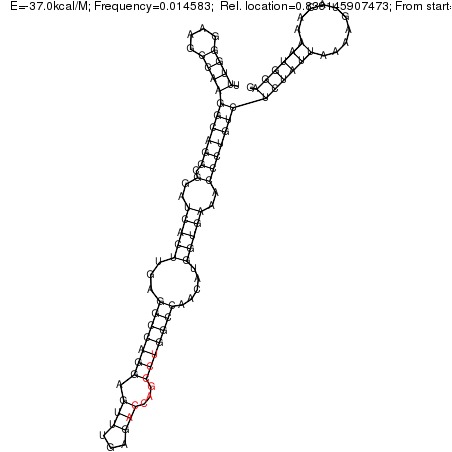

Supplement: Additional file 5 — Figure S4. Secondary structures for sequence region 0.7 to 1.0 of full length for the selected 32 eRNAs (see Fig. 7). [file 1471-2164-12-S3-S18-S5.zip › Figure S4/MFE/ACCAGCCU_rank-1_10436915.jpg]

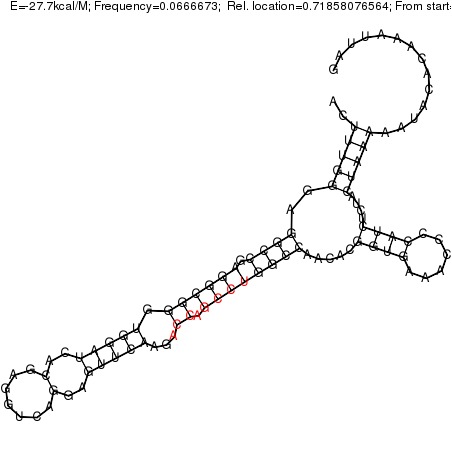

Supplement: Additional file 5 — Figure S4. Secondary structures for sequence region 0.7 to 1.0 of full length for the selected 32 eRNAs (see Fig. 7). [file 1471-2164-12-S3-S18-S5.zip › Figure S4/MFE/ACCAGCCU_rank-20_149363691.jpg]

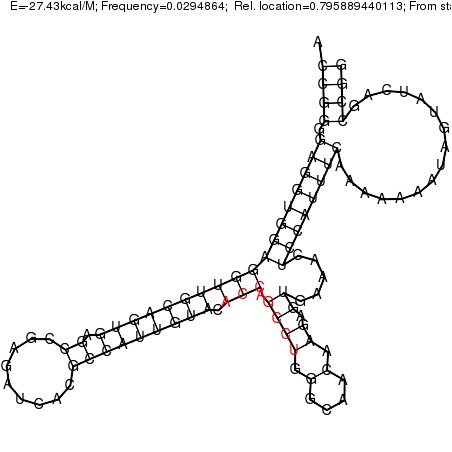

Supplement: Additional file 5 — Figure S4. Secondary structures for sequence region 0.7 to 1.0 of full length for the selected 32 eRNAs (see Fig. 7). [file 1471-2164-12-S3-S18-S5.zip › Figure S4/MFE/ACCAGCCU_rank-21_21758081.jpg]

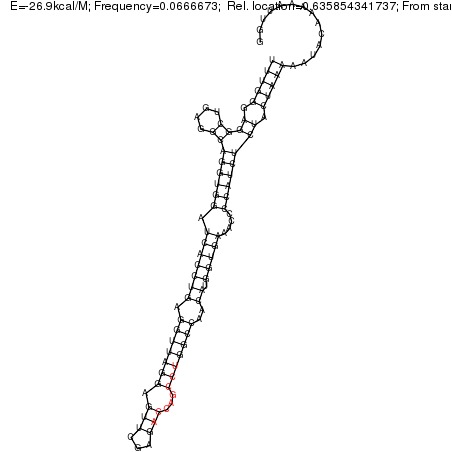

Supplement: Additional file 5 — Figure S4. Secondary structures for sequence region 0.7 to 1.0 of full length for the selected 32 eRNAs (see Fig. 7). [file 1471-2164-12-S3-S18-S5.zip › Figure S4/MFE/ACCAGCCU_rank-22_149363691.jpg]

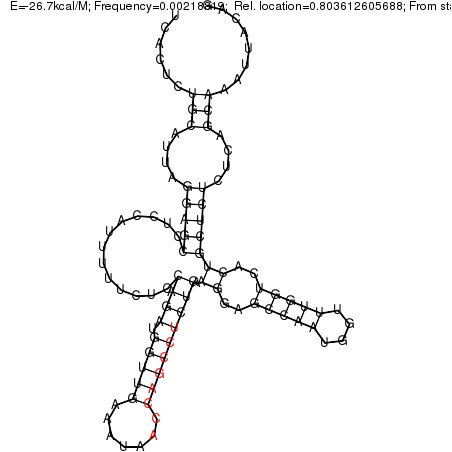

Supplement: Additional file 5 — Figure S4. Secondary structures for sequence region 0.7 to 1.0 of full length for the selected 32 eRNAs (see Fig. 7). [file 1471-2164-12-S3-S18-S5.zip › Figure S4/MFE/ACCAGCCU_rank-23_16550670.jpg]

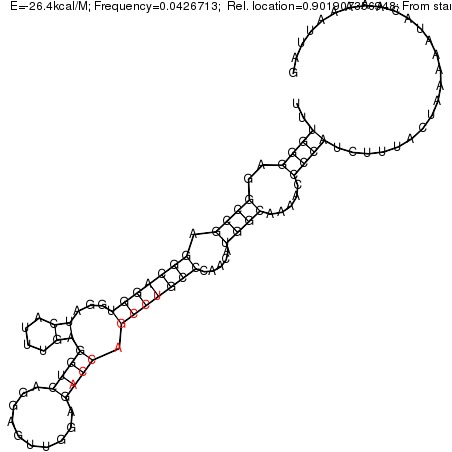

Supplement: Additional file 5 — Figure S4. Secondary structures for sequence region 0.7 to 1.0 of full length for the selected 32 eRNAs (see Fig. 7). [file 1471-2164-12-S3-S18-S5.zip › Figure S4/MFE/ACCAGCCU_rank-24_37547428.jpg]
